# Earthworm Is a Versatile and Sustainable Biocatalyst for Organic Synthesis

**DOI:** 10.1371/journal.pone.0105284

**Published:** 2014-08-22

**Authors:** Zhi Guan, Yan-Li Chen, Yi Yuan, Jian Song, Da-Cheng Yang, Yang Xue, Yan-Hong He

**Affiliations:** School of Chemistry and Chemical Engineering, Southwest University, Chongqing, P. R. China; University of Sydney, Australia

## Abstract

A crude extract of earthworms was used as an eco-friendly, environmentally benign, and easily accessible biocatalyst for various organic synthesis including the asymmetric direct aldol and Mannich reactions, Henry and Biginelli reactions, direct three-component aza-Diels-Alder reactions for the synthesis of isoquinuclidines, and domino reactions for the synthesis of coumarins. Most of these reactions have never before seen in nature, and moderate to good enantioselectivities in aldol and Mannich reactions were obtained with this earthworm catalyst. The products can be obtained in preparatively useful yields, and the procedure does not require any additional cofactors or special equipment. This work provides an example of a practical way to use sustainable catalysts from nature.

## Introduction

The exploitation of new catalysts in an environmentally benign manner has become crucially important in recent years [1]. Nature is an extraordinary chemist who evolves its catalysts over millions of years [2]. Such catalysts are models of energy-efficient, environmentally benign chemical agents, as virtually all do their work under mild conditions and generate few waste products [3]. However, the development and use of these natural catalysts in organic chemistry are very limited. To date, the most common biocatalytic methods use either whole-cells or isolated enzymes for a given chemical transformation. Each method has advantages and challenges for both laboratory-scale and industrial-scale chemistry [4]. The use of whole-cell systems benefits from the ability to use low-cost and renewable feedstocks, and co-factor addition and regeneration are not necessary. However, whole-cell systems require expensive equipment and tedious work-up because of large volumes, and have low productivity. More importantly, the accumulation of products and by-products may be toxic to the cell. Besides, the cell membrane may act as a mass transport barrier between the substrates and the enzymes [5]. Compared to whole cells, isolated enzymes offer several benefits, including simpler reaction apparatus, higher productivity owing to higher catalyst concentration, and simpler product purification [6]. Thus, isolated enzymes are usually more efficient in bioconversion than whole cells. However, enzyme purification involves expensive strategies, usually employing costly and polluting chemicals, being also laborious and time-consuming, which restricts the use of isolated enzymes, making it difficult to scale-up [7]. Therefore, it is obligatory to find easy methods to supply the gaps.

Using crude extracts of organisms as biocatalysts may be the right solution for they are environmentally-benign, inexpensive and simple in preparation. Moreover, generally, a relatively crude preparation is far more stable than a highly purified enzyme [8]. Some efforts have been made to use crude extracts of organisms as biocatalysts for organic synthesis. For example, a crude extract of the button mushroom (*Agaricus bisporus*) was employed to catalyse the domino reaction between phenol and cyclic 1,3-dicarbonyls using atmospheric oxygen as the oxidizing agent and yielding annulated benzofuranes [9]. A crude horse liver homogenate was used as a biocatalyst to catalyse the acylglucuronide of mycophenolic acid [10]. Crude preparations of various plants were used to catalyse bioreduction of carbonyl compounds and alcohol biooxidation [11]. However, the reported reaction types catalysed by crude extracts of organisms are very limited.

Earthworms are harmless creatures that live in the soil. They are eco-friendly playing the significant role in decomposing organic wastes, enhancing soil fertility, and improving soil drainage. Earthworms have been used from ancient times in oriental countries as drugs for prevention and treatment of various diseases, and have found applications such as intracystic calculus-contraction and releasing-stimulating agent, anti-choloplania agent, parturifacient, hair growth tonic, antifebrile, spasm-treating agent, hemiplegia-treating agent, urination improving agent, anti-bronchial asthma agent, anti-hypertension agent, therapeutic medicament for thrombosis and others [12].

The most thoroughly studied enzymes from earthworms are proteases that are secreted by alimentary tract of earthworm [13]. For example, an earthworm, *Lumbricus rubellus*, produces alkaline serine proteases that show higher activity and stability than trypsins. These enzymes are stable at temperature below 60°C over a wide range of pH 2–11, and are strongly resistant to organic solvents and detergents. Moreover, they retain full activity for long years at room temperature. They act on various proteins, such as elastin as well as fibrin, and some peptides, such as β-amyloid 1–40 and solubilized actual fibrin clots of whole blood in a rat's vena cava [14].

**Figure 1:**
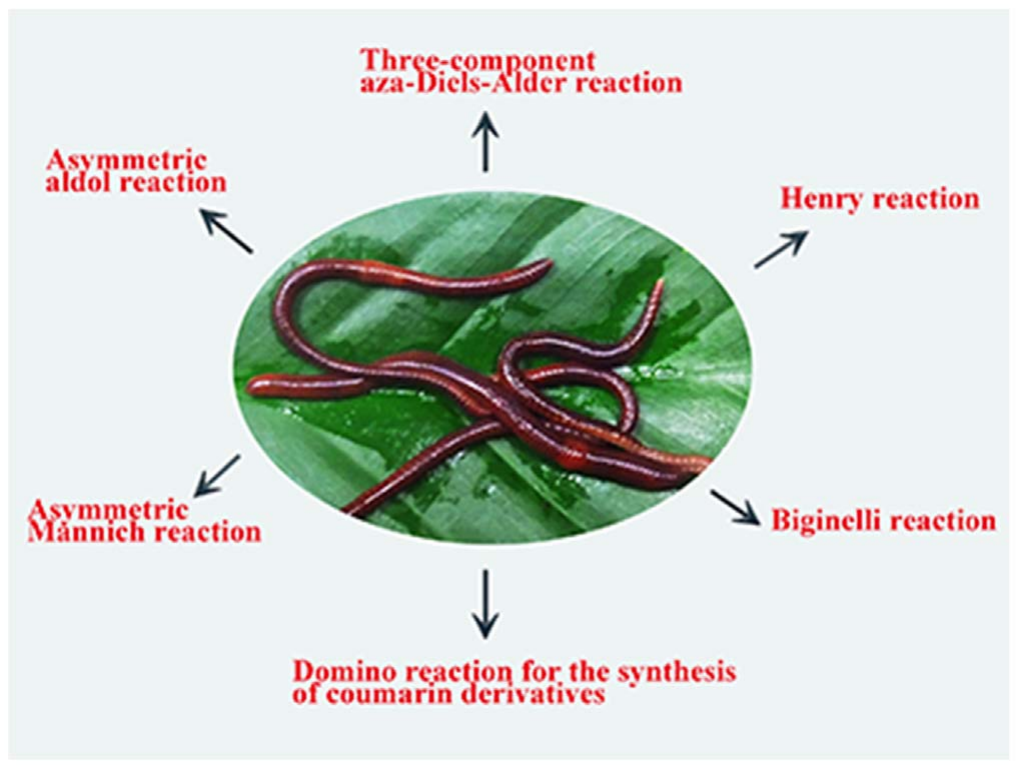
The earthworm-catalysed organic reactions.

However, the use of earthworms as a source of biocatalysts for biotransformation is very limited. Nakajima et al. reported that one of the earthworm serine protease acts on the hydrolysis of triacylglycerols [15]. They also reported the stereoselective reduction of carbonyl compounds using the cell-free extract from earthworms (*Lumbricus rubellus*) in the presence of NADH or NADPH as a coenzyme [16]. Recently, Santos et al. describe β-carboline imine reductions in high yields and enantiomeric excesses employing the cell-free extract from earthworms (*Eisenia foetida*) in the presence of NADPH [17]. Herein, we report that a crude extract of earthworms is a versatile biocatalyst for the asymmetric direct aldol and Mannich reactions, Henry and Biginelli reactions, direct three-component aza-Diels-Alder reactions for the synthesis of isoquinuclidines, and domino reactions for the synthesis of coumarins (**Fig. 1**). The earthworm species we used is *Eisenia foetida* (Annelida, Oligochaeta), known as red worm.

## Results and Discussion

First, we developed a very simple procedure for the preparation of a crude extract from earthworms. The fresh earthworms were homogenized with deionized water. The supernatant obtained after centrifugation was concentrated by dialysis against solid sucrose, dried, and ground to get an extract powder, which was used directly to catalyse the different reactions.

### The crude earthworm extract catalysed direct asymmetric aldol reactions

**Figure 2:**
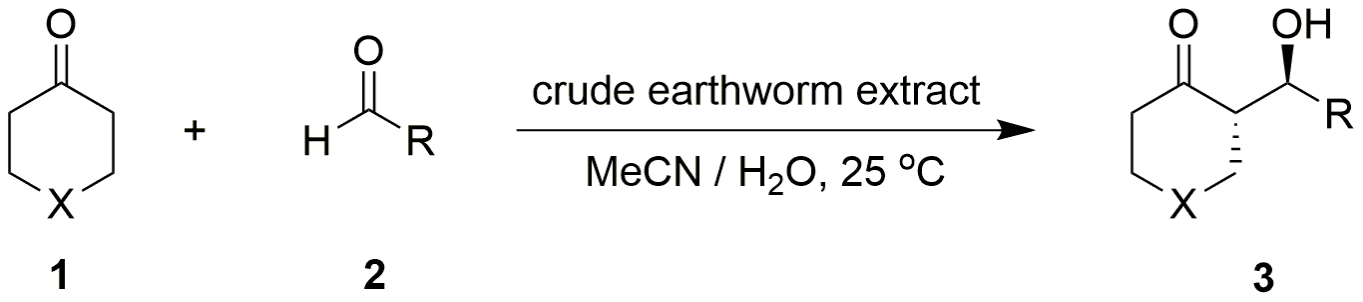
The crude earthworm extract catalysed direct asymmetric aldol reactions.

The catalytic asymmetric aldol reaction is one of the most powerful methods for the construction of chiral β-hydroxy carbonyl compounds. The crude earthworm extract could catalyse the direct asymmetric aldol reactions of aromatic aldehydes with cyclic and heterocyclic ketones in MeCN in the presence of a small amount of water (**Fig. 2**). The yields of 53–96%, enantioselectivities of 80–91% ee (for *anti* isomers), and diastereoselectivities of 81∶19->99∶1 (*anti/syn*) were achieved under the optimized conditions (**Table 1**, entries 1–8). The reaction showed good substrate adaptability to different aromatic aldehydes with electron-withdrawing and electron-donating groups, as well as heterocyclic ketones containing oxygen and sulfur. Moreover, in the absence of the crude earthworm extract, only a trace amount of product was observed in the model aldol reaction of 4-cyanobenzaldehyde and cyclohexanone even after 120 h (**Table 1**, entry 10), indicating that the crude earthworm extract indeed catalysed the aldol reaction in asymmetric manner.

**Table 1:** The crude earthworm extract catalysed direct asymmetric aldol reactions^a^.

Entry	X	R	Product	Time (h)	Yield (%)^b^	dr (*anti*∶*syn*)^c^	ee (*anti*) (%)^c^
1	CH_2_	3-CNC_6_H_4_	**3a**	144	96	91∶9	91
2	CH_2_	4-ClC_6_H_4_	**3b**	139	60	92∶8	91
3	CH_2_	4-CF_3_C_6_H_4_	**3c**	120	84	81∶19	90
4	CH_2_	2,4-Cl_2_C_6_H_3_	**3d**	192	82	>99∶1	90
5	CH_2_	3-MeOC_6_H_4_	**3e**	192	53	88∶12	91
6	O	4-NO_2_C_6_H_4_	**3f**	103	75	83∶17	80
7	S	4-CNC_6_H_4_	**3g**	117	65	93∶7	90
8	CH_2_	4-CNC_6_H_4_	**3h**	120	92	87∶13	88
9 (Un-optimized conditions)^d^	CH_2_	4-CNC_6_H_4_	**3h**	120	78	78∶22	82
10 (No catalyst)^e^	CH_2_	4-CNC_6_H_4_	**3h**	120	Trace	—	—
11 (100°C pretreated crude earthworm extract)^f^	CH_2_	4-CNC_6_H_4_	**3h**	120	39	80∶20	85
12 (Cu^2+^pretreated crude earthworm extract)^g^	CH_2_	4-CNC_6_H_4_	**3h**	120	11	78∶22	86
13 (Cu^2+^ as catalyst)^h^	CH_2_	4-CNC_6_H_4_	**3h**	120	trace	—	—

a Optimized reaction conditions: aldehyde (0.50 mmol), ketone (7.50 mmol), the crude earthworm extract (75 mg), deionized water (0.05 mL), and MeCN (0.95 mL) at 25°C.

b Yield of the isolated product after silica gel chromatography.

c Determined by chiral HPLC analysis performed using Chiralpak AD-H, AS-H, or Chiralcel OD-H column, by comparing the retention time with those of known compounds (For details, please see the Supplementary material).

d Un-optimized conditions: 4-cyanobenzaldehyde (0.50 mmol), cyclohexanone (2.50 mmol), the crude earthworm extract (50 mg), deionized water (0.05 mL), and MeCN (0.95 mL) at 30°C.

e The reaction was conducted in the absence of the crude earthworm extract, otherwise under the same conditions as entry 9.

f The crude earthworm extract (50 mg) in deionized water (3 mL) was stirred at 100°C for 24 h, and then water was removed under reduced pressure before use. The reaction was conducted under the same conditions as entry 9.

g The crude earthworm extract (50 mg) in Cu^2+^ solution (0.25 M) [anhydrous CuSO_4_ (120 mg) in deionized water (3 mL)] was stirred at 30°C for 24 h, and then water was removed under reduced pressure before use. The reaction was conducted under the same conditions as entry 9.

h The reaction was conducted using anhydrous CuSO_4_ (120 mg) as a catalyst instead of the crude earthworm extract, otherwise under the same conditions as entry 9.

To verify whether the enzyme(s) in the earthworm extract catalysed the aldol reaction, some control experiments were performed (**Table 1**, entries 11–13). Firstly, the high temperature (at 100°C for 24 h) pretreated earthworm extract was used to catalyse the model reaction, which gave the product in a low yield of 39% with 80∶20 dr and 85% ee (**Table 1**, entry 11), demonstrating that high temperature treatment caused a decrease of the catalytic ability of the earthworm extract on the aldol reaction. Secondly, the Cu^2+^ pretreated crude earthworm extract was also used to catalyse the model aldol reaction, and only a low yield of 11% was obtained with78∶22 dr and 86% ee (**Table 1**, entry 12). Meanwhile, to exclude the effect of Cu^2+^ on the reaction, Cu^2+^ alone was used to catalyse the model aldol reaction, and only a trace amount of product was observed on TLC (**Table 1**, entry 13), proving that Cu^2+^ did not catalyse this transformation. The above control experiments showed that high temperature and metal ion Cu^2+^, as deactivation factors of enzyme, could greatly decrease the catalytic ability of the crude earthworm extract on the model aldol reaction. Thus, it can be inferred that the enzyme(s) in the crude earthworm extract catalysed the aldol reaction.

### The crude earthworm extract catalysed direct asymmetric Mannich reactions

**Figure 3:**
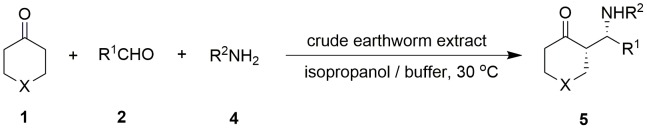
The crude earthworm extract catalysed direct asymmetric Mannich reactions.

The asymmetric Mannich reaction is a powerful synthetic strategy to prepare chiral β-amino ketones and aldehydes with perfect atom economy through the loss of a molecule of water, and the reaction products are versatile intermediates in the synthesis of chiral amines [18]. The crude earthworm extract could catalyse the direct asymmetric Mannich reactions in isopropanol/buffer (**Fig. 3**). Various substituted aromatic aldehydes, arylamines and cyclic or heterocyclic ketones participated in the reaction smoothly. Mannich products were obtained in yields of 42–83% with diastereoselectivities of 43∶57–87∶13 (*syn*/*anti*) and enantioselectivities of 44–76% ee (for *syn* isomers) under the optimized conditions (**Table 2**, entries 1–6). In the absence of the crude earthworm extract, the product was obtained only in 29% yield with 38∶62 dr (*syn*/*anti*) for the model Mannich reaction of 4-nitrobenzaldehyde, aniline and cyclohexanone after 72 h (**Table 2**, entry 8), indicating that the crude earthworm extract indeed catalysed the Mannich reaction in asymmetric manner.

**Table 2:** The crude earthworm extract catalysed direct asymmetric Mannich reactions^a^.

Entry	X	R^1^	R^2^	Product	Time (h)	Yield (%)^b^	dr (*syn*∶*anti*)^c^	ee (*syn*) (%)^c^
1	CH_2_	4-NO_2_C_6_H_4_	3-BrC_6_H_4_	**5a**	96	42	87∶13	76
2	CH_2_	4-NO_2_C_6_H_4_	4-ClC_6_H_4_	**5b**	71	66	84∶16	74
3	CH_2_	4-NO_2_C_6_H_4_	3-MeC_6_H_4_	**5c**	47	75	79∶21	73^d^
4	CH_2_	4-NO_2_C_6_H_4_	4-MeC_6_H_4_	**5d**	47	79	74∶26	71^d^
5	S	4-ClC_6_H_4_	C_6_H_5_	**5e**	96	70	43∶57	44
6	CH_2_	4-NO_2_C_6_H_4_	C_6_H_5_	**5f**	72	83	79∶21	75
7 (Un-optimized conditions)^e^	CH_2_	4-NO_2_C_6_H_4_	C_6_H_5_	**5f**	72	82	63∶37	62
8 (No catalyst)^f^	CH_2_	4-NO_2_C_6_H_4_	C_6_H_5_	**5f**	72	29	38∶62	0

a Optimized reaction conditions: aldehyde (0.50 mmol), arylamine (0.55 mmol), cyclohexanone (5.00 mmol) or tetrahydrothiopyran-4-one (1.00 mmol), buffer (NaH_2_PO_4_-Na_2_HPO_4_, 0.20 M, pH = 7.53, 0.05 mL), isopropanol (0.95 mL), and the crude earthworm extract (50 mg) at 30°C.

b Yield of the isolated product after silica gel chromatography.

c Determined by chiral HPLC analysis performed using Chiralpak AD-H or Chiralcel OD-H column, by comparing the retention time with those of known compounds (For details, please see the Supplementary material).

d The chiral HPLC did not show baseline separation of peaks.

e Un-optimized conditions: 4-nitrobenzaldehyde (0.50 mmol), aniline (0.55 mmol), cyclohexanone (5.00 mmol), deionized water (0.10 mL), isopropanol (0.90 mL) and the crude earthworm extract (50 mg) at 30°C.

f The reaction was carried out in the absence of the crude earthworm extract, otherwise under the same conditions as entry 7.

### The crude earthworm extract catalysed Henry reactions

**Figure 4:**
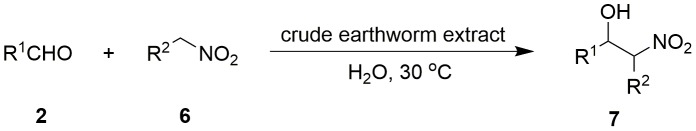
The crude earthworm extract catalysed Henry reactions.

The Henry or nitroaldol reaction is an important atom-economical methodology to furnish a β-nitro alcohol. The crude earthworm extract could catalyse Henry reactions in water (**Fig. 4**). Various aromatic aldehydes participated in the reactions with nitromethane, nitroethane and nitropropane giving corresponding products in yields of 41–92% (**Table 3**, entries 1–6). In the absence of the crude earthworm extract, only a trace amount of product was observed (**Table 3**, entry 7), indicating that the crude earthworm extract indeed catalysed the Henry reaction. Unfortunately, there was no obvious enantiomeric excess of the products observed by the chiral HPLC analysis.

**Table 3:** The crude earthworm extract catalysed Henry reactions^a^.

Entry	R^1^	R^2^	Product.	Time (h)	Yield (%)^b^	dr (*syn*∶*anti*)^c^
1	3-CNC_6_H_4_	H	**7a**	160	85	—
2	4-NO_2_C_6_H_4_	H	**7b**	93	88	—
3	2-NO_2_C_6_H_4_	H	**7c**	93	85	—
4	4-CNC_6_H_4_	Me	**7d**	118	92	65∶35
5	4-NO_2_C_6_H_4_	Et	**7e**	118	41	66∶34
6	4-CNC_6_H_4_	H	**7f**	120	88	—
7 (No catalyst)	4-CNC_6_H_4_	H	**7f**	104	Trace	—

a Reaction conditions: aldehyde (0.50 mmol), nitroalkane (2.50 mmol), the crude earthworm extract (50 mg), deionized water (1.00 mL) at 30°C.

b Yield of the isolated product after silica gel chromatography.

c Determined by ^1^HNMR in comparison with those of known compounds (For details, please see the Supplementary material).

### The crude earthworm extract catalysed Biginelli reactions

**Figure 5:**
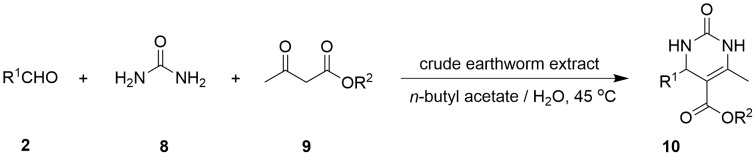
The crude earthworm extract catalysed Biginelli reactions.

The Biginelli reaction is an important three-component reaction for construction of dihydropyrimidinones that are widely used in the pharmaceutical industry. The crude earthworm extract could catalyse Biginelli reactions in *n*-butyl acetate/water (**Fig. 5**). Various substituted aromatic aldehydes reacted with urea and acetoacetate giving corresponding products in yields of 14–76% with enantioselectivities of 0–57% ee (**Table 4**, entries 1–7). In the absence of the crude earthworm extract, the product was only obtained in 6% yield (**Table 4**, entry 9), indicating that the crude earthworm extract indeed catalysed the Biginelli reaction.

**Table 4:** The crude earthworm extract catalysed Biginelli reactions^a^.

Entry	R^1^	R^2^	Product	Time (h)	Yield (%)^b^	ee (%)^c^
1	4-MeOC_6_H_4_	Et	**10a**	169	44	0
2	3-NO_2_C_6_H_4_	Et	**10b**	118	76	29 (R)
3	4-FC_6_H_4_	Et	**10c**	69	53	0
4	3-ClC_6_H_4_	Et	**10d**	119	49	10 (R)
5	2-ClC_6_H_4_	Me	**10e**	140	69	20^d^
6	3-NO_2_-C_6_H_4_	Me	**10f**	103	14	57^d^
7	C_6_H_5_	Et	**10g**	72	65	0
8 (Un-optimized conditions)^e^	C_6_H_5_	Et	**10g**	72	44	0
9 (No catalyst)^f^	C_6_H_5_	Et	**10g**	72	6	0

a Optimized reaction conditions: aldehyde (0.50 mmol), urea (1.00 mmol), acetoacetate (1.00 mmol), deionized water (0.30 mL), *n*-butyl acetate (0.70 mL) and the crude earthworm extract (75 mg) at 45°C.

b Yield of isolated product after silica gel chromatography.

c Determined by chiral HPLC analysis performed using Chiralpak AD-H column, by comparing the retention time with those of known compounds (For details, please see the Supplementary material).

d The absolute configuration was not determined.

e Un-optimized conditions: benzaldehyde (0.50 mmol), urea (1.00 mmol), ethyl acetoacetate (1.50 mmol), deionized water (0.05 mL), *n*-butyl acetate (0.95 mL) and the crude earthworm extract (100 mg) at 45°C.

f The reaction was carried out in the absence of the crude earthworm extract, otherwise under the same conditions as entry 8.

### The crude earthworm extract catalysed domino reactions for the synthesis of coumarin derivatives

**Figure 6:**
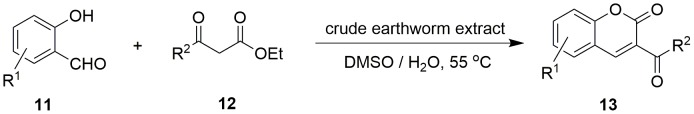
The crude earthworm extract catalysed domino reactions for the synthesis of coumarin derivatives.

Coumarin derivatives are widely used in pharmaceutical and commercial applications. The crude earthworm extract could catalyse the reactions of various salicylaldehyde derivates with different β-keto esters to afford a series of coumarin derivatives (**Fig. 6**). This reaction is a domino process that comprises Knoevenagel and intramolecular transesterification steps. Various coumarin derivatives were obtained in yields of 32–87% in DMSO/water under the optimized conditions (**Table 5**, entries 1–7). In the absence of the crude earthworm extract, only a trace amount of product was observed (**Table 5**, entry 9), indicating that the crude earthworm extract indeed catalysed the domino reaction.

**Table 5:** The crude earthworm extract catalysed domino reactions for the synthesis of coumarin derivatives^a^.

Entry	R^1^	R^2^	Product	Time (h)	Yield (%)^b^
1	5-NO_2_	Ph	**13a**	71	80
2	5-Cl	Ph	**13b**	72	86
3	5-OMe	Ph	**13c**	102	75
4	1-naphth	Ph	**13d**	96	87
5	H	Ph	**13e**	99	80
6	H	OEt	**13f**	127	32
7	H	Me	**13g**	127	51
8 (Un-optimized conditions)^c^	H	Me	**13g**	100	21
9 (No catalyst)^d^	H	Me	**13g**	100	Trace

a Optimized reaction conditions: **11** (4.00 mmol), 1**2** (1.00 mmol), the crude earthworm extract (150 mg), deionized water (0.15 mL) and DMSO (0.85 mL) at 55°C.

b Yield of the isolated product after silica gel chromatography.

c Un-optimized conditions: salicylaldehyde (3.00 mmol), ethyl acetoacetate (1.00 mmol), the crude earthworm extract (100 mg), deionized water (0.10 mL) and DMSO (0.90 mL) at 30°C.

d The reaction was carried out in the absence of the crude earthworm extract, otherwise under the same conditions as entry 8.

### The crude earthworm extract catalysed aza-Diels-Alder reactions for the synthesis of isoquinuclidine derivatives

**Figure 7:**
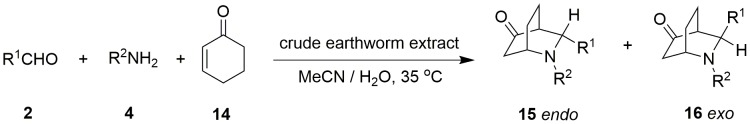
The crude earthworm extract catalysed aza-Diels-Alder reactions.

Isoquinuclidine (azabicyclo[2.2.2]octane) has received considerable attention due to their presence in numerous complex natural products and pharmaceuticals. The crude earthworm extract could catalyse three-component aza-Diels-Alder reactions for the synthesis of isoquinuclidine derivatives (**Fig. 7**). The reactions of aromatic aldehydes, aromatic amines and cyclohexenone in MeCN/H_2_O gave corresponding isoquinuclidines in excellent yields of 86–99%, and all reactions afforded the *endo* isomers as the major products with *endo*/*exo* ratio ranged from 68∶32 to 65∶35 (**Table 6**, entries 1–5). In the absence of the crude earthworm extract, only a trace amount of product was observed (**Table 6**, entry 6), indicating that the crude earthworm extract indeed catalysed the aza-Diels-Alder reaction. Unfortunately, there was no obvious enantiomeric excess of the products observed by the chiral HPLC analysis.

**Table 6:** The crude earthworm extract catalysed aza-Diels-Alder reactions^a^.

Entry	R^1^	R^2^	Product	Yield (15+16) (%)^b^	*endo* 15/*exo* 16^c^
1	3-FC_6_H_4_	4-MeOC_6_H_4_	**15a**, **16a**	97	68∶32
2	4-FC_6_H_4_	4-MeOC_6_H_4_	**15b**, **16b**	93	66∶34
3	4-FC_6_H_4_	C_6_H_5_	**15c**, **16c**	99	67∶33
4	3-ClC_6_H_4_	4-MeOC_6_H_4_	**15d**, **16d**	92	65∶35
5	4-ClC_6_H_4_	4-MeOC_6_H_4_	**15e**, **16e**	86	68∶32
6 (No catalyst)	4-ClC_6_H_4_	4-MeOC_6_H_4_	**15e**, **16e**	Trace	—

a Reaction conditions: aromatic aldehyde (0.50 mmol), aromatic amine (1.50 mmol), cyclohexenone (1.50 mmol), and the crude earthworm extract (100 mg) in MeCN (0.93 mL) and deionized water (0.07 mL) at 35°C for 96 h.

b Yield of the isolated products after silica gel chromatography.

c Calculated according to the isolated weights of **15** and **16**. The *endo* or *exo* was determined by ^1^HNMR in comparison with those of known compounds (For details, please see the Supplementary material).

In addition, we tested the stability of the crude earthworm extract powder by repeating the direct asymmetric aldol reaction of 4-cyanobenzaldehyde and cyclohexanone using the crude earthworm extract that had been stored at 4°C for 6 months. The activity and selectivity did not decrease, demonstrating that the crude earthworm extract is reasonably stable.

## Conclusion

Nature is a vital source of catalysts, but the use of natural catalysts in organic synthesis is very limited, mainly due to the difficulties in isolation and purification of enzymes. Herein we demonstrated that using crude extracts of organisms as biocatalysts is a promising choice for development of natural catalysts. The crude earthworm extract is applicable to unusually wide scope of reaction types and substrates, including the asymmetric direct aldol and Mannich reactions, Henry and Biginelli reactions, direct three-component aza-Diels-Alder reactions for the synthesis of isoquinuclidines, and domino reactions for the synthesis of coumarins. Most of these reactions have never before seen in nature. The products can be obtained in preparatively useful yields, and moderate to good enantioselectivities for aldol and Mannich reactions were obtained with this earthworm catalyst. The procedure does not require any additional cofactors or special equipment. The main advantages of using the crude earthworm extract as a catalyst are eco-friendly, environmentally benign, safe, cheap, easily accessible and stable. This work provides an example of a practical way to use sustainable catalysts from nature. It would have a transformative effect on streamlining the practice of organic synthesis.

## Methods

### Ethics statement

Live earthworms (*Eisenia foetida*, the common Chinese name: “Daping II”) were purchased from Tianjin Cheng Gong earthworm farm (Tianjin, China).

### Procedure for the preparation of the crude earthworm extract

The live earthworms were rinsed 5 times with tap water to let the internal dirt discharge as much as possible, and then rinsed twice with deionized water. The clean earthworms (200 mL, measured with a graduated beaker) were homogenized with equal volume ice-cold deionized water (200 mL). The homogenate was centrifuged (RCF = 4250 g, for 5 min). The collected supernatants were concentrated by dialysis against solid sucrose at 4°C. The concentrate was placed in a tray and blown with an electric fan at 17–25°C until it turned into a paste. The paste was further dried under vacuum, and then ground into powder with a porcelain mortar to give a gray powder (52.1 g). The extract powder was stored at 4°C for use.

### The reaction condition screening program

The reaction conditions for each type of chemical transformation were optimized using corresponding model reactions. These optimizations included: **the solvent optimization** (Different solvents such as CH_2_Cl_2_, CHCl_3_, methyl phenyl ether, toluene, *n*-butyl acetate, EtOAc, 1,4-dioxane, MTBE, THF, cyclohexane, DMSO, EtOH, MeOH, *i*-PrOH, H_2_O and solvent-free, were screened, respectively, for each type of reaction to find out the optimal solvent); **the water content optimization** [The water contents of 0%, 5%, 10%, 15%, 20%, 25%, 30%, 35%, 40%, 45%, 50% (v/v) in the reaction system were screened, respectively, for each type of reaction to find out the optimal water content]; **the substrate molar ratio optimization** (Different molar ratios of substrates were screened, respectively, for each type of reaction to find out the suitable molar ratio of substrates); **the crude earthworm extract loading optimization** (Different amounts of crude earthworm extract were screened, respectively, for each type of reaction to choose the optimal catalyst loading); **the phosphate buffer optimization** [NaH_2_PO_4_-Na_2_HPO_4_ buffer, 0.20 M, different pH from 5 to 11 were separately used to replace the optimized water content for each type of chemical transformation to find out the optimal phosphate buffer pH. For the direct asymmetric Mannich reaction, adding buffer (NaH_2_PO_4_-Na_2_HPO_4_, 0.20 M, pH = 7.53, 0.05 mL) could improve the yield and enantioselectivity, however, for other types of chemical transformation, addition of phosphate buffer did not lead to obvious improvement of the results. Thus, the reaction medium consisting of phosphate buffer (NaH_2_PO_4_-Na_2_HPO_4_, 0.20 M, pH = 7.53, 0.05 mL) and isopropanol (the optimal solvent) (0.95 mL) was used for the Mannich reaction, while deionized water/organic solvent was used for other reactions. For the Henry reaction, the optimal solvent was water]; **the temperature optimization** (The influence of different temperature on each type of reaction was also investigated to find out the optimal temperature).

The selected optimal reaction conditions for each type of chemical transformation were used in the following procedures.

### General procedure for the crude earthworm extract catalysed reactions

#### The aldol reactions

A round-bottom flask was charged with the crude earthworm extract (75 mg), aldehyde (0.50 mmol), ketone (7.50 mmol) and MeCN (0.95 mL), to which deionized water (0.05 mL) was introduced. The resultant mixture was stirred at 25°C for the specified reaction time and monitored by TLC. The reaction was terminated by filtration (with buchner funnel and qualitative filter paper), and ethyl acetate (20 mL) was employed to wash the filter cake. The filtrate was concentrated under reduced pressure. The residue was purified by silica gel flash column chromatography (petroleum ether/ethyl acetate) to give the product.

#### The Mannich reactions

The crude earthworm extract (50 mg) was added to a round-bottom flask containing an aromatic aldehyde (0.50 mmol), arylamine (0.55 mmol), cyclohexanone (5.00 mmol) or tetrahydrothiopyran-4-one (1.00 mmol), buffer (NaH_2_PO_4_-Na_2_HPO_4_, 0.20 M, pH = 7.53, 0.05 mL) and isopropanol (0.95 mL). The resultant mixture was stirred at 30°C for the specified reaction time and monitored by TLC. The reaction was terminated by filtration (with buchner funnel and qualitative filter paper), and ethyl acetate (10 mL) was employed to wash the filter cake. The filtrate was concentrated under reduced pressure. The residue was purified by silica gel flash column chromatography (petroleum ether/ethyl acetate) to give the product.

#### The Henry reactions

A round-bottom flask was charged with the crude earthworm extract (50 mg), aldehyde (0.50 mmol), nitroalkane (2.50 mmol), and deionized water (1.00 mL). The resultant mixture was stirred at 30°C and monitored by TLC. After a specified reaction time, saturated brine (15 mL) was added to the reaction, and the mixture was extracted four times with ethyl acetate (15 mL). The combined extracts were dried over anhydrous Na_2_SO_4_, and the solvents were then removed under reduced pressure. The residue was purified by silica gel flash column chromatography (petroleum ether/ethyl acetate) to give the product.

#### The Biginelli reactions

A round-bottom flask was charged with aldehyde (0.50 mmol), urea (1.00 mmol), acetoacetate (1.00 mmol), *n*-butyl acetate (0.70 mL) and the crude earthworm extract (75 mg), to which deionized water (0.30 mL) was introduced. The resultant mixture was stirred at 45°C and monitored by TLC. After a specified reaction time, ethanol (20 mL) was added to the reaction mixture to dissolve the crude products with stirring. The mixture was then filtrated (with buchner funnel and qualitative filter paper), and ethanol (20 mL) was employed to wash the filter cake. The filtrate was concentrated under reduced pressure. The residue was dissolved in ethyl acetate, and washed with saturated brine to remove the excess urea. The organic phase was dried over anhydrous Na_2_SO_4_, and the solvents were then removed under reduced pressure. The solid residue was recrystallized from ethyl acetate-petroleum ether to give the first part of the product. The mother liquor was concentrated under reduced pressure, and the residue was then purified by silica gel flash column chromatography (petroleum ether/ethyl acetate) to give the second part of the product. The reaction yield refers to the combination of the two parts of product above.

#### The domino reactions for the synthesis of coumarin derivatives

A round-bottom flask was charged with the crude earthworm extract (150 mg), salicylaldehyde derivative (4.00 mmol), β-keto ester (1.00 mmol), DMSO (0.85 mL) and deionized water (0.15 mL). The resultant mixture was stirred at 55°C for the specified reaction time and monitored by TLC. The reaction was terminated by filtration (with buchner funnel and qualitative filter paper), and ethyl acetate was employed to wash the filter cake. Saturated brine (15 mL) was then added to the filtrate, and the filtrate was extracted three times with ethyl acetate (15 mL). The combined extracts were dried over anhydrous Na_2_SO_4_, and the solvents were then removed under reduced pressure. The residue was purified by silica gel flash column chromatography (petroleum ether/ethyl acetate) to give the product.

#### The aza-Diels-Alder reactions

The crude earthworm extract (100 mg) was added to a round-bottom flask containing aldehyde (0.50 mmol), aromatic amine (1.50 mmol), cyclohexenone (1.50 mmol), MeCN (0.93 mL), and deionized water (0.07 mL). The resultant mixture was stirred at 35°C for the specified reaction time and monitored by TLC. The reaction was terminated by filtration (with buchner funnel and qualitative filter paper), and ethyl acetate (10 mL) was employed to wash the filter cake. The filtrate was then washed twice with water (10 mL), and the aqueous phase was back-extracted twice with ethyl acetate (10 mL). Combined organic phase was dried over anhydrous Na_2_SO_4_, and the solvents were then removed under reduced pressure. The residue was purified by silica gel flash column chromatography (petroleum ether/ethyl acetate) to give the product.

## Supporting Information

Table S1 List of the obvious difference between aldol products *syn*-3 and *anti*-3 on ^1^H NMR and chiral HPLC (Table 1, entries 1–8).
(DOC)

Table S2 List of the difference between Mannich products *syn*-5 and *anti*-5 on chiral HPLC (Table 2, entries 1–6).
(DOC)

Table S3 List of the obvious difference between Henry products *syn*-7 and *anti*-7 on ^1^H NMR (Table 3, entries 4 and 5).
(DOC)

Table S4 List of the obvious difference between Aza-Diels-Alder products *endo*-15 and *exo*-16 on ^1^H NMR (Table 6, entries 1–5).
(DOC)

Data S1 ^1^H NMR, ^13^C NMR and HPLC data of products.
(DOC)

Materials S1 The materials and general methods.
(DOC)

Spectra S1 ^1^H and ^13^C NMR-spectra and HPLC chart.
(DOC)
