# Supplementary material for: Earthworm Is a Versatile and Sustainable Biocatalyst for Organic Synthesis
Source: PLoS One. 2014 Aug 22;9(8):e105284. doi: 10.1371/journal.pone.0105284 (PMC4141794; doi:10.1371/journal.pone.0105284)
Supplement: Table S1 — List of the obvious difference between aldol products syn-3 and anti-3 on 1H NMR and chiral HPLC (Table 1, entries 1–8). (DOC) [file pone.0105284.s001.doc]

**Supporting Information Table S1**

Earthworm is a versatile and sustainable biocatalyst for organic synthesis

Zhi Guan, Yan-Li Chen, Yi Yuan, Jian Song, Da-Cheng Yang, Yang Xue, Yan-Hong He*

School of Chemistry and Chemical Engineering, Southwest University, Chongqing, 400715, P. R. China

Fax: (+86)23-68254091; Email: heyh@swu.edu.cn

**Table S1 List of the obvious difference between aldol products *syn*-3 and *anti*-3 on ^1^H NMR and chiral HPLC (Table 1, entries 1-8)**

| Ref. | Product | ^1^HNMR (CDCl_3_) -CHOH | | Chiral HPLC | | | | *syn* | *anti* |
| --- | --- | --- | --- | --- | --- | --- | --- | --- | --- |
|  |  | *syn* | *anti* | λ (nm) | column | heptane/  *i*PrOH | flow (mL/min) | t_Rmajor_/  t_Rminor_ (min) | t_Rmajor_/  t_Rminor_  (min) |
| [[1](#_ENREF_2)] | **3a** | 5.44 (brs) | 4.83 (d, *J* = 8.2 Hz) | 220 | AD-H | 90:10 | 1.0 | 23.9/  19.0 | 27.4/  35.2 |
| [[2](#_ENREF_3), 3] | **3b** | 5.34 (brs) | 4.76 (d, *J* = 8.5 Hz) | 254 | AD-H | 90:10 | 1.0 | 12.2/  10.5 | 19.2/  16.6 |
| [[3](#_ENREF_4)] | **3c** | 5.45 (brs) | 4.85 (d, *J* = 8.6 Hz) | 254 | AD-H | 90:10 | 1.0 | 8.1/  9.3 | 14.9/  12.1 |
| [[2](#_ENREF_3), [4](#_ENREF_5)] | **3d** | 5.65 (brs) | 5.29 (d, *J* = 7.6 Hz) | 220 | AS-H | 90:10 | 1.0 | 15.8/  12.4 | 23.2/  19.1 |
| [[5](#_ENREF_6)] | **3e** | 5.35 (brs) | 4.76 (d, *J* = 8.8 Hz) | 221 | AD-H | 90:10 | 0.5 | 30.7/  34.3 | 58.7/  54.2 |
| [[6](#_ENREF_7)] | **3f** | 5.55 (brs) | 4.99 (d, *J* = 8.1 Hz) | 254 | AD-H | 80:20 | 1.0 | 22.7/  25.9 | 39.2/  33.1 |
| [[6](#_ENREF_7)] | **3g** | 5.50 (brs) | 5.00 (d, *J* = 8.1 Hz) | 254 | OD-H | 92:8 | 1.0 | 32.2/  35.6 | 44.1/  67.2 |
| [[5](#_ENREF_6), [7](#_ENREF_8)] | **3h** | 5.43 (brs) | 4.85 (d, *J* = 8.0 Hz) | 254 | AD-H | 80:20 | 0.5 | 20.7/  18.2 | 29.8/  23.7 |

For references please see the Supporting Information Data S1.
