# Supplementary material for: Earthworm Is a Versatile and Sustainable Biocatalyst for Organic Synthesis
Source: PLoS One. 2014 Aug 22;9(8):e105284. doi: 10.1371/journal.pone.0105284 (PMC4141794; doi:10.1371/journal.pone.0105284)
Supplement: Table S2 — List of the difference between Mannich products syn-5 and anti-5 on chiral HPLC (Table 2, entries 1–6). (DOC) [file pone.0105284.s002.doc]

**Supporting Information Table S2**

Earthworm is a versatile and sustainable biocatalyst for organic synthesis

Zhi Guan, Yan-Li Chen, Yi Yuan, Jian Song, Da-Cheng Yang, Yang Xue, Yan-Hong He*

School of Chemistry and Chemical Engineering, Southwest University, Chongqing, 400715, P. R. China

Fax: (+86)23-68254091; Email: heyh@swu.edu.cn

**Table S2 List of the difference between Mannich products *syn*-5 and *anti*-5 on chiral HPLC (Table 2, entries 1-6)**

| Ref. | Product | Chiral HPLC | | | | *syn* | *anti* |
| --- | --- | --- | --- | --- | --- | --- | --- |
|  |  | λ (nm) | column | heptane/*i*PrOH | Flow (mL/min) | t_Rmajor_/t_Rminor_ (min) | t_Rmajor_/t_Rminor_ (min) |
| [8] | **5a** | 254 | AD-H | 85:15 | 1.0 | 24.5/16.7 | 18.6/13.1 |
| [8] | **5b** | 254 | AD-H | 85:15 | 1.0 | 28.9/26.8 | 21.3/21.8 |
| [8] | **5c** | 254 | AD-H | 85:15 | 1.0 | 21.5/ 18.4 | 17.7/14.3 |
| [8] | **5d** | 254 | AD-H | 85:15 | 1.0 | 22.4/21.5 | 20.2/18.1 |
| [9] | **5e** | 254 | OD-H | 95:5 | 1.0 | 26.8/50.1 | 15.8/14.8 |
| [9] | **5f** | 254 | AD-H | 80:20 | 1.0 | 17.7/13.6 | 16.4/11.2 |

For references please see the Supporting Information Data S1.
