# Supplementary material for: Earthworm Is a Versatile and Sustainable Biocatalyst for Organic Synthesis
Source: PLoS One. 2014 Aug 22;9(8):e105284. doi: 10.1371/journal.pone.0105284 (PMC4141794; doi:10.1371/journal.pone.0105284)
Supplement: Table S3 — List of the obvious difference between Henry products syn-7 and anti-7 on 1H NMR (Table 3, entries 4 and 5). (DOC) [file pone.0105284.s003.doc]

**Supporting Information Table S3**

Earthworm is a versatile and sustainable biocatalyst for organic synthesis

Zhi Guan, Yan-Li Chen, Yi Yuan, Jian Song, Da-Cheng Yang, Yang Xue, Yan-Hong He*

School of Chemistry and Chemical Engineering, Southwest University, Chongqing, 400715, P. R. China

Fax: (+86)23-68254091; Email: heyh@swu.edu.cn

**Table S3 List of the obvious difference between Henry products *syn*-7 and *anti*-7 on ^1^H NMR (Table 3, entries 4 and 5)**

| Ref. | Product | ^1^HNMR (CDCl_3_), -CHOH | |
| --- | --- | --- | --- |
|  |  | *syn* | *anti* |
| [[12]](#_ENREF_1) | **7d** | 5.12 (dd, *J_1_* = 8.6 Hz, *J_2_* = 3.8 Hz) | 5.50 (brs) |
| [[10]](#_ENREF_1) | **7e** | 5.19 (d, *J* = 8.4 Hz) | 5.34 (d, *J* = 4.4 Hz) |

For references please see the Supporting Information Data S1.
