# Supplementary material for: Earthworm Is a Versatile and Sustainable Biocatalyst for Organic Synthesis
Source: PLoS One. 2014 Aug 22;9(8):e105284. doi: 10.1371/journal.pone.0105284 (PMC4141794; doi:10.1371/journal.pone.0105284)
Supplement: Table S4 — List of the obvious difference between Aza-Diels-Alder products endo-15 and exo-16 on 1H NMR (Table 6, entries 1–5). (DOC) [file pone.0105284.s004.doc]

**Supporting Information Table S4**

Earthworm is a versatile and sustainable biocatalyst for organic synthesis

Zhi Guan, Yan-Li Chen, Yi Yuan, Jian Song, Da-Cheng Yang, Yang Xue, Yan-Hong He*

School of Chemistry and Chemical Engineering, Southwest University, Chongqing, 400715, P. R. China

Fax: (+86)23-68254091; Email: heyh@swu.edu.cn

**Table S4 List of the obvious difference between Aza-Diels-Alder products *endo*-15 and *exo*-16 on ^1^H NMR (Table 6, entries 1-5)**

| Ref. | Product | ^1^HNMR (CDCl_3_) | |
| --- | --- | --- | --- |
|  |  | *endo* | *exo* |
| [22] |  | 4.59 (brs, 1H) | 4.70 (brs, 1H) |
| [22] |  | 4.56 (brs, 1H) | 4.68 (brs, 1H) |
| [22] |  | 4.66 (brs, 1H) | 4.76 (brs, 1H) |
| [23] |  | 4.54 (brs, 1H) | 4.66 (brs, 1H) |
| [23] |  | 4.55 (d, *J* = 2.3 Hz ,1H) | 4.66 (d, *J* = 2.3 Hz, 1H) |

For references please see the Supporting Information Data S1.
