# Supplementary material for: Earthworm Is a Versatile and Sustainable Biocatalyst for Organic Synthesis
Source: PLoS One. 2014 Aug 22;9(8):e105284. doi: 10.1371/journal.pone.0105284 (PMC4141794; doi:10.1371/journal.pone.0105284)
Supplement: Data S1 — 1H NMR, 13C NMR and HPLC data of products. (DOC) [file pone.0105284.s005.doc]

**Supporting Information Data S1**

Earthworm is a versatile and sustainable biocatalyst for organic synthesis

Zhi Guan, Yan-Li Chen, Yi Yuan, Jian Song, Da-Cheng Yang, Yang Xue, Yan-Hong He*

School of Chemistry and Chemical Engineering, Southwest University, Chongqing, 400715, P. R. China

Fax: (+86)23-68254091; Email: heyh@swu.edu.cn

**Data S1 The ^1^H NMR, ^13^C NMR and HPLC data of products**

**Aldol products 3a-h (Table 1, entries 1-8)**

**2-(Hydroxy-(3-Cyanophenyl-phenyl)methyl) cyclohexan-1-one (****3a):** [[^1^](#_ENREF_2)]

^1^H NMR (300 MHz, CDCl_3_): *δ* = 7.46-7.65 (m, 4H), 4.83 (d, *J* = 8.2 Hz, 1H), 4.15 (d, *J* = 1.7 Hz, 1H), 2.55-2.64 (m, 1H), 2.34-2.51 (m, 2H), 2.08-2.14 (m, 1H), 1.81-1.85 (m, 1H), 1.53-1.70 (m, 3H), 1.28-1.40 (m, 1H) ppm. ^13^C NMR (75 MHz, CDCl_3_): *δ* = 214.8, 142.6, 131.5, 131.4, 130.6, 129.1, 118.7, 112.3, 69.7, 57.0, 42.5, 30.6, 27.6, 24.5 ppm. The enantiomeric excess was determined by HPLC (Daicel Chiralpak AD-H, hexane/isopropanol = 90:10, flow rate 1.0 mL/min, λ = 220 nm), *syn*-diastereomer: t_Rmajor_ = 23.899 min, t_Rminor_ = 19.002 min; *anti*-diastereomer: t_Rmajor_ = 27.384 min, t_Rminor_ = 35.186 min.

**2-(Hydroxy-(4-chlorophenyl-phenyl)methyl)cyclohexan-1-one (****3b):** [[^2^](#_ENREF_3)^, 3]^

^1^H NMR (300 MHz, CDCl_3_): *δ* = 7.30-7.32 (m, 2H), 7.24-7.26 (m, 2H), 4.74-4.77 (d, *J* = 8.5 Hz, 1H), 4.03 (br, 1H), 2.45-2.55 (m, 2H), 2.33-2.37 (m 1H), 2.06-2.09 (m, 1H), 1.51-1.70 (m, 3H), 1.26-1.29 ( m, 1H ) ppm. ^13^C NMR (75 MHz, CDCl_3_): *δ* = 215.2, 139.5, 133.4, 128.3, 74.0, 57.3, 42.6, 30.7, 27.7, 24.6 ppm. The enantiomeric excess was determined by HPLC (Daicel Chiralpak AD-H, hexane/isopropanol = 90:10, flow rate 1.0 mL/min, *λ* = 254 nm), *syn*-diastereomer: t_Rmajor_ = 12.198 min, t_Rminor_ = 10.549 min; *anti*-diastereomer: t_Rmajor_ = 19.172 min, t_Rminor_ = 16.640 min.

**2-(Hydroxy-(4-trifluoromethyl-phenyl)methyl)cyclohexan-1-one (3c):** [[^3^](#_ENREF_4)]

^1^H NMR (300 MHz, CDCl_3_): *δ* = 7.60-7.62 (m, 2H), 7.44-7.46 (m, 2H), 4.85 (d, *J* = 8.6 Hz, 1H), 4.10 (br, 1H), 2.56-2.65 (m, 1H), 2.47-2.52 (m, 1H), 2.33-2.42 (m, 1H), 2.07-2.13 (m, 1H), 1.80-1.84 (m, 1H), 1.53-1.69 (m 3H), 1.26-1.39 (m, 1H) ppm. ^13^C NMR (75 MHz, CDCl_3_): *δ* =215.1, 144.9, 130.5, 127.3, 126.0, 125.3, 70.1, 57.2, 42.6, 30.7, 27.7, 24.6 ppm. The enantiomeric excess was determined by HPLC (Daicel Chiralpak AD-H, hexane/isopropanol = 90:10, flow rate 1.0 mL/min, *λ* = 254 nm), *syn*-diastereomer: t_Rmajor_ = 8.121 min, t_Rminor_ = 9.313 min; *anti*-diastereomer: t_Rmajor_ = 14.894 min, t_Rminor_ = 12.138 min.

**2-(Hydroxy-(2,4-dichlorophenyl-phenyl)methyl)cyclohexan-1-one (****3d):** [[^2^](#_ENREF_3), [^4^](#_ENREF_5)]

^1^H NMR (300 MHz, CDCl_3_): *δ* = 7.48-7.51 (m, 1H), 7.27-7.35 (m, 2H), 5.28 (d, *J* = 7.6 Hz, 1H), 4.11 (br, 1H), 2.58-2.67 (m, 1H), 2.44-2.49 (m, 1H), 2.28-2.39 (m, 1H), 2.06-2.12 (m, 1H), 1.82-1.84 (m, 1H), 1.54-1.70 (m, 4H) ppm. ^13^C NMR (75 MHz, CDCl_3_): *δ* = 215.0, 137.8, 133.7, 129.2, 128.8, 127.6, 70.0, 57.5, 42.6, 30.3, 27.7, 24.8 ppm. The enantiomeric excess was determined by HPLC (Daicel Chiralpak AS-H, hexane/isopropanol = 90:10, flow rate 1.0 mL/min, *λ* = 220 nm), *syn*-diastereomer: t_Rmajor_ = 15.760 min, t_Rminor_ = 12.356 min; *anti*-diastereomer: t_Rmajor_ = 23.224 min, t_Rminor_ = 19.095 min.

**2-(Hydroxy-(3-methoxy-phenyl)-methyl)cyclohexan-1-one (****3e):** [[^5^](#_ENREF_6)]

^1^H NMR (300 MHz, CDCl_3_): *δ* = 7.22-7.28 (m, 1H), 6.81-6.90 (m, 3H), 4.76 (d, *J* = 8.8 Hz, 1H), 4.00 (s, 1H), 3.81 (s, 3H), 2.56-2.65 (m, 1H), 2.30-2.50 (m, 2H), 2.04-2.11 (m, 1H), 1.76-1.80 (m, 1H), 1.50-1.69 (m, 3H), 1.22-1.27 (m, 1H) ppm. ^13^C NMR (75 MHz, CDCl_3_): *δ* = 215.5, 159.6, 142.5, 129.3, 119.4, 113.3, 112.4, 74.6, 57.3, 55.2, 42.6, 30.8, 27.8, 24.6 ppm. The enantiomeric excess was determined by HPLC (Daicel Chiralpak AD-H, hexane/isopropanol = 90:10, flow rate 0.5 mL/min, *λ* = 221 nm), *syn*-diastereomer: t_Rmajor_ = 30.679 min, t_Rminor_ = 34.341 min; *anti*-diastereomer: t_Rmajor_ = 58.742 min, t_Rminor_ = 54.256 min.

**3-(Hydroxy(4-nitrophenyl)methyl)tetrahydropyran-4-one (3f) :** [[^6^](#_ENREF_7)]

^1^H NMR (400 MHz, CDCl_3_) δ = 8.23 (d, *J* = 8.8 Hz, 2H), 7.52 (d, *J* = 8.5 Hz, 2H), 5.55 (s, 1H), 4.99 (d, *J* = 8.1 Hz, 1H), 4.24-4.18 (m, 1H), 3.83 (s, 1H), 3.75 (dd, *J* = 13.8, 6.5 Hz, 2H), 3.46 (dd, *J* = 11.4, 9.9 Hz, 1H), 2.94-2.86 (m, 1H), 2.73-2.65 (m, 1H), 2.56-2.50 (m, 1H) ppm. ^13^C NMR (101 MHz, CDCl_3_): *δ* = 209.2, 148.2, 147.8, 147.4, 127.5, 126.4, 123.8, 71.3, 69.8, 68.9, 68.3, 67.6, 57.7, 57.3, 43.1, 42.9 ppm. The enantiomeric excess was determined by HPLC (Daicel Chiralpak AD-H, hexane/isopropanol = 80:20, flow rate 1.0 mL/min, *λ* = 254 nm), *syn*-diastereomer: t_Rmajor_ = 22.664 min, t_Rminor_ = 25.955 min; *anti*-diastereomer: t_Rmajor_ = 39.203 min, t_Rminor_ = 33.059 min.

**3-(Hydroxy(4-Cyanophenyl)methyl)-tetrahydrothiopyran-4-one (3g):** [[^6^](#_ENREF_7)]

^1^H NMR (400 MHz, CDCl_3_) δ = 7.68 (d, *J* = 8.4 Hz, 2H), 7.48 (d, *J* = 8.2 Hz, 2H), 5.47 (d, *J* = 2.1 Hz, 1H), 5.00 (d, *J* = 8.1 Hz, 1H), 3.80-3.78 (m, 1H), 3.61 (s, 1H), 2.98 (dt, *J* = 6.3, 4.5 Hz, 3H), 2.84-2.77 (m, 2H), 2.66-2.61 (m, 1H), 2.50 (ddd, *J* = 13.6, 4.8, 2.1 Hz, 1H) ppm. ^13^C NMR (101 MHz, CDCl_3_) δ = 211.3, 145.7, 132.4, 132.2, 127.7, 118.6, 112.1, 73.4, 59.4, 44.7, 32.8, 30.8 ppm. The enantiomeric excess was determined by HPLC (Daicel Chiralpak OD-H, hexane/isopropanol = 92:8, flow rate 1.0 mL/min, λ = 254 nm), *syn*-diastereomer: t_R_major = 32.188 min, t_R_minor = 35.637 min; *anti*-diastereomer: t_R_major = 44.145 min, t_R_minor = 67.214 min.

**2-(Hydroxy-(4-Cyanophenyl-phenyl)methyl) cyclohexan-1-one (****3h):** [[^5^](#_ENREF_6), [^7^](#_ENREF_8)]

^1^H NMR (300 MHz, CDCl_3_): *δ* = 7.63-7.65 (m, 2H), 7.44-7.46 (m, 2H), 4.84 (d, *J* = 8.0 Hz, 1H), 4.08 (br, 1H), 2.35-2.58 (m, 3H), 2.09-2.12 (m, 1H), 1.81-1.84 (m, 1H), 1.50-1.69 (m, 3H), 1.26-1.41 (m, 2H) ppm. ^13^C NMR (75 MHz, CDCl_3_): *δ* = 214.7, 146.4, 132.1, 127.7, 118.7, 111.6, 74.1, 57.1, 42.6, 30.6, 27.6, 24.6 ppm. The enantiomeric excess was determined by HPLC (Daicel Chiralpak AD-H, hexane/isopropanol = 80:20, flow rate 0.5 mL/min, *λ* = 254 nm), *syn*-diastereomer: t_Rmajor_ = 20.754 min, t_Rminor_ = 18.225 min; *anti*-diastereomer: t_Rmajor_ = 29.771 min, t_Rminor_ = 23.713 min.

**Mannich products 5a-f (Table 2, entries 1-6)**

**2-[(3-bromophenylamino)-(4-nitrophenyl)methyl]cyclohexanone (5a):** ^[8]^

^1^H NMR (400 MHz, CDCl_3_): *δ* = 8.18-8.16 (m, 2H), 7.55-7.53 (m, 2H), 6.95-6.91 (m, 1H), 6.82-6.79 (m, 1H), 6.68-6.67 (m, 1H), 6.42-6.39 (m, 1H), 4.81-4.62 (m, 2H), 2.88-2.83 (m, 1H), 2.47-2.31 (m, 2H), 2.07-2.03 (m, 2H), 1.66-1.52 (m, 4H) ppm; ^13^C NMR (101 MHz, CDCl_3_): *δ* = 210.6, 148.8, 148.0, 147.2, 130.5, 128.6, 123.8, 121.2, 116.7, 112.5, 57.1, 42.4, 29.0, 27.0, 24.9 ppm; The enantiomeric excess was determined by HPLC (Daicel Chiralpak AD-H, hexane/isopropanol = 85:15, flow rate 1.0 mL/min, *λ* = 254 nm), *syn*-diastereomer: t_R_ = 24.490 min (major), 16.680 min (minor), *anti*-diastereomer: t_R_ = 18.574 min (major), 13.134 min (minor).

**2-[(4-Chlorophenylamino)-(4-nitrophenyl)methyl]cyclohexanone (5b):** ^[8]^

^1^H NMR (400 MHz, CDCl_3_): *δ* = 8.21-8.15 (m, 2H), 7.55-7.53 (m, 2H), 7.04-7.02 (m, 2H), 6.45-6.42 (m, 2H), 4.80-4.62 (m, 2H), 2.87-2.84 (m, 1H), 2.44-2.33 (m, 2H), 2.07-2.04 (m, 2H), 1.65-1.54 (m, 4H) ppm; ^13^C NMR (101 MHz, CDCl_3_): *δ* = 210.7, 149.0, 147.2, 145.3, 129.0, 128.6, 123.8, 123.0, 114.6, 57.3, 56.8, 42.4, 32.2, 27.9, 24.9 ppm; The enantiomeric excess was determined by HPLC (Daicel Chiralpak AD-H, hexane/isopropanol = 85:15, flow rate 1.0 mL/min, *λ* = 254 nm), *syn*-diastereomer: t_R_ = 28.923 min (major), 26.851 min (minor), *anti*-diastereomer: t_R_ = 21.318 min (major), 21.808 min (minor).

**2-[(3-Methylphenylamino)-(4-nitrophenyl)methyl]cyclohexanone** **(5c):** ^[8]^

^1^H NMR (400 MHz, CDCl_3_): *δ* = 8.16-8.14 (m, 2H), 7.57-7.55 (m, 2H), 6.99-6.95 (m, 1H), 6.54-6.52 (m, 1H), 6.38 (s, 1H), 6.31-6.29 (m, 1H), 4.85-4.67 (m, 2H), 2.88-2.85 (m, 1H), 2.43-2.32 (m, 2H), 2.20 (s, 3H), 2.07-1.65 (m, 6H) ppm; ^13^C NMR (101 MHz, CDCl_3_): *δ* = 210.7, 149.8, 139.0, 128.6, 123.6, 119.3, 115.0, 114.4, 111.0, 110.4, 57.1, 56.3, 42.5, 32.0, 29.1, 27.1, 25.0 ppm; The enantiomeric excess was determined by HPLC (Daicel Chiralpak AD-H, hexane/isopropanol = 85:15, flow rate 1.0 mL/min, *λ* = 254 nm), *syn*-diastereomer: t_R_ = 21.539 min (major), 18.428 min (minor), *anti*-diastereomer: t_R_ = 17.717 min (major), 14.298 min (minor).

**2-[(4-Methylphenylamino)-(4-nitrophenyl)methyl]cyclohexanone (5d):** ^[8]^

^1^H NMR (400 MHz, CDCl_3_): *δ* = 8.15-8.13 (m, 2H), 7.58-7.53 (m, 2H), 6.90-6.88 (m, 2H), 6.43-6.40 (m, 2H), 4.83-4.58 (m, 2H), 2.86-2.82 (m, 1H), 2.42-2.32 (m, 2H), 2.17 (s, 3H), 2.06-1.92 (m, 3H), 1.75-1.60 (m, 3H) ppm; ^13^C NMR (101 MHz, CDCl_3_): *δ* = 210.8, 150.0, 147.0, 129.8, 128.6, 127.7, 123.6, 113.7, 57.5, 57.1, 42.4, 31.9, 27.1, 24.5, 20.4 ppm; The enantiomeric excess was determined by HPLC (Daicel Chiralpak AD-H, hexane/isopropanol = 85:15, flow rate 1.0 mL/min, *λ* = 254 nm), *syn*-diastereomer: t_R_ = 22.410 min (major), 21.531 min (minor), *anti*-diastereomer: t_R_ = 20.236 min (major), 18.089 min (minor)

**3-[(4-chlorophenyl)(phenylamino)methyl]dihydro-2H-thiopyran-4(3H)-one** **(5e):** ^[9]^

^1^H NMR (300 MHz, CDCl_3_): *δ* = 7.27 (m, 4H), 7.12-7.07 (m, 2H), 6.71-6.66 (m, 1H), 6.57-6.52 (m, 2H), 5.18-4.94 (m, 1H), 4.44 (s, 1H), 3.14-2.95 (m, 5H), 2.73-2.67 (m, 2H) ppm; ^13^C NMR (75 MHz, CDCl_3_): *δ* = 208.5, 146.6, 139.2, 133.2, 129.2, 128.9, 128.6, 118.4, 114.1, 58.5, 57.1, 44.3, 31.7, 30.3 ppm; The enantiomeric excess was determined by HPLC (Daicel Chiralcel OD-H, hexane/isopropanol = 95:5, flow rate 1.0 mL/min, *λ* = 254 nm), *syn*-diastereomer: t_R_ = 26.859 min (major), 50.149 min (minor), *anti*-diastereomer: t_R_ = 15.787 min (major), 14.802 min (minor).

**2-[(4-nitrophenyl)(phenylamino)methyl]yclohexanone** **(5f):** ^[9]^

^1^H NMR (300 MHz, CDCl_3_): *δ* = 8.16-8.13 (m, 2H), 7.58-7.54 (m, 2H), 7.10-7.05 (m, 2H), 6.70-6.65 (m, 1H), 6.51-6.48 (m, 2H), 4.86-4.65 (m, 2H), 2.86-2.82 (m, 1H), 2.42-2.32 (m, 2H), 2.06-1.92 (m, 3H), 1.66-1.60 (m, 3H) ppm; ^13^C NMR (75 MHz, CDCl_3_): *δ* = 210.6, 149.5, 147.0, 146.6, 129.1, 128.6, 123.6, 118.3, 114.0, 57.1, 56.2, 42.4, 29.0, 27.0, 24.9 ppm; The enantiomeric excess was determined by HPLC (Daicel Chiralpak AD-H, hexane/isopropanol = 80:20, flow rate 1.0 mL/min, *λ* = 254 nm), *syn*-diastereomer: t_R_ = 17.698 min (major), 13.582 min (minor), *anti*-diastereomer: t_R_ = 16.396 min (major), 11.258 min (minor).

**Henry products 7a-f (Table 3, entries 1-6)**

**3-(1-hydroxy-2-nitroethyl)benzonitrile (7a):** [[^10]^](#_ENREF_1)

^1^H NMR (300 MHz, CDCl_3_): *δ* =7.76 (s,1H),7.68-7.65 (m, 2H), 7.56-7.52 (m, 1H), 5.55-5.51 (m, 1H), 4.61-4.56 (m, 2H), 3.40-3.39 (m, 1H) ppm. ^13^C NMR (75 MHz, CDCl_3_): *δ* =139.8, 132.5, 130.4, 129.9, 129.7, 118.2, 113.1, 80.8, 69.8 ppm.

**2-nitro-1-(4-nitrophenyl)ethanol (7b):** [[^10]^](#_ENREF_1)

^1^H NMR (300 MHz, CDCl_3_) δ = 8.24 (d, *J* = 8.6 Hz, 2H), 7.64 (d, *J* = 8.5 Hz, 2H), 5.63 (d, *J* = 3.0 Hz, 1H), 4.70–4.57 (m, 2H), 3.55 (s, 1H). ^13^C NMR (75 MHz, CDCl_3_) δ = 147.9, 145.1, 126.9, 124.1, 80.6, 69.9.

**2-nitro-1-(2-nitrophenyl)ethanol (7c):** [[^11]^](#_ENREF_1)

^1^H NMR (300 MHz, CDCl_3_): *δ* = 8.07-8.05 (m, 1H), 7.96-7.94 (m, 1H), 7.77-7.73 (m, 1H), 7.57-7.53 (m, 1H), 6.05 (dd, *J_1_* = 1.7 Hz, *J_2_* = 1.7 Hz, 1H), 4.88-4.84 (m, 1H), 4.59-4.53 (m, 1H), 3.50 (br, 1H) ppm. ^13^C NMR (75 MHz, CDCl_3_): *δ* =147.0, 134.5, 134.4, 129.7, 128.7, 124.9, 80.2, 66.8 ppm.

**4-(1-hydroxy-2-nitropropyl)benzonitrile (7d ):** [[^12]^](#_ENREF_1)

*syn*-Isomer as the major product: ^1^H NMR (300 MHz, CDCl_3_) δ = 7.71-7.66 (m, 2H), 7.56-7.52 (m, 2H), 5.12 (dd, J_1_ = 8.6 Hz, J_2_ = 3.8 Hz, 1H), 4.78–4.70 (m, 1H), 3.55 (d, J = 3.8 Hz, 1H), 1.45 (d, *J* = 6.8 Hz, 1H), 1.34 (d, *J* = 6.8 Hz, 2H). ^13^C NMR (75 MHz, CDCl_3_) δ = 143.70, 132.7, 127.8, 118.3, 112.5, 87.8, 75.2, 16.1.

**2-nitro-1-(4-nitrophenyl)butan-1-ol (7e):** [[^10]^](#_ENREF_1)

*syn*-Isomer as the major product: ^1^H NMR (300 MHz, CDCl_3_) δ = 8.25 (m, 2H), 7.59 (d, *J* = 8.6 Hz, 2H), 5.19 (d, *J* = 8.4 Hz, 1H), 4.68–4.53 (m, 1H), 3.18 (m, 1H), 1.95–1.82 (m, 1H), 1.59–1.34 (m, 1H), 0.97–0.89 (m, 3H). ^13^C NMR (75 MHz, CDCl_3_) δ = 148.2, 145.6, 127.8, 124.1, 94.5, 74.3, 23.8, 10.0.

**4-(1-hydroxy-2-nitroethyl)benzonitrile (7f):** [[^10]^](#_ENREF_1)

^1^H NMR (300 MHz, CDCl_3_): *δ* =7.72-7.70 (m, 2H), 7.57-7.55 (m, 2H), 5.57-5.53 (m, 1H), 4.60-4.55 (m, 2H), 3.32-3.31 (m, 1H) ppm. ^13^C NMR (75 MHz, CDCl_3_): *δ* =143.4, 132.8 (2C), 126.9 (2C), 118.3, 112.6, 80.7, 70.1 ppm.

**Biginelli products 10a-g (Table 4, entries 1-7)**

**5-Ethoxycarbonyl-6-methyl-4-(4-methoxyphenyl)-3,4-dihydropyrimidin-2(1H)-one (10a):** ^[13]^

^1^H NMR (300 MHz, DMSO-*d_6_*): *δ* = 9.16 (s, 1H), 7.68 (s, 1H), 7.12-7.15 (d, *J* = 8.4 Hz, 2H), 6.85 (d, *J* = 8.4 Hz, 2H), 5.08 (d, *J* = 2.5 Hz, 1H), 3.96 (q, *J* = 7.0 Hz, 2H), 3.70 (s, 3H), 2.23 (s, 3H), 1.08 (t, *J* = 7.0 Hz, 3 H) ppm. ^13^C NMR (75 MHz, DMSO-*d_6_*): *δ=* 165.7, 152.6, 148.6, 142.3, 136.8, 129.3, 126.9, 126.5, 99.8, 59.6, 54.0, 21.0, 18.1, 14.5 ppm.

**(R)-5-Ethoxycarbonyl-6-methyl-4-(3-nitrophenyl)-3,4-dihydropyrimidin-2(1H)-one (10b):** ^[14, 15]^

^1^H NMR (300 MHz, DMSO-*d_6_*): *δ* = 9.37 (s, 1H), 8.07-8.13 (m, 2H), 7.90 (s, 1H), 7.61-7.70 (m, 2H), 5.29 (d, *J* = 3.1 Hz, 1H), 3.94-4.02 (m, 2H), 2.26 (s, 3H), 1.08 (t, *J* = 7.1 Hz, 3H) ppm. ^13^C NMR (75 MHz, DMSO-*d_6_*): *δ =* 165.5, 152.2, 149.8, 148.1, 147.4, 133.4, 130.6, 122.8, 121.4, 98.7, 59.8, 53.9, 18.3, 14.4 ppm. The enantiomeric excess was determined by HPLC (Daicel Chiralpak AD-H, hexane/isopropanol = 90:10, flow rate 1.0 mL/min, *λ* = 254 nm), t_Rmajor_ = 34.064 min, t_Rminor_ = 44.829 min.

**5-Ethoxycarbonyl-6-methyl-4-(4-fluorophenyl)-3,4-dihydropyrimidin-2(1H)-one (10c):** ^[13]^

^1^H NMR (300 MHz, DMSO-*d_6_*): *δ* = 9.26 (s, 1H), 7.79 (s, 1H), 7.25-7.30 (m, 2H), 7.13-7.18 (m, 2H), 5.16 (d, *J* = 2.7 Hz, 1H), 3.97 (q, *J* = 7.0 Hz, 2H), 2.27 (s, 3H), 1.09 (t, *J* = 7.1 Hz, 3H) ppm. ^13^C NMR (75 MHz, DMSO-*d_6_*): *δ =* 165.6, 163.3, 160.1, 152.4, 148.9, 141.5, 128.7, 128.6, 115.6, 115.4, 99.5, 59.6, 53.8, 18.2, 14.4 ppm.

**(R)-5-Ethoxycarbonyl-6-methyl-4-(3-chlorophenyl)-3,4-dihydropyrimidin-2(1H)-one (10d):** ^[14, 16]^

^1^H NMR (300 MHz, DMSO-*d_6_*): *δ* = 9.23 (s, 1H), 7.75 (s, 1H), 7.25-7.32 (m, 2H), 7.18-7.22 (m, 1H), 7.12-7.14 (m, 1H), 5.09 (d, *J* = 3.0 Hz, 1H), 3.90-3.93 (m, 2H), 2.19 (s, 3H), 1.02 (t, *J* = 7.1 Hz, 3 H) ppm. ^13^C NMR (75 MHz, DMSO-*d_6_*): *δ* = 165.6, 152.4, 149.4, 147.6, 132.3, 130.8, 127.6, 126.6, 125.3, 99.0, 59.7, 54.0, 18.2, 14.4 ppm. The enantiomeric excess was determined by HPLC (Daicel Chiralpak AD-H, hexane/isopropanol = 80:20, flow rate 0.5 mL/min, *λ* = 254 nm), t_Rmajor_ = 15.111 min, t_Rminor_ = 16.662 min.

**5-Methoxycarbonyl-6-methyl-4-(2-chlorophenyl)-3,4-dihydropyrimidin-2(1H)-one (10e):** [[^17^](#_ENREF_5)]

^1^H NMR (300 MHz, DMSO-*d_6_*): *δ* = 9.32 (s, 1H), 7.73 (s, 1H), 7.39-7.41 (m, 1H), 7.27-7.30 (m, 3H), 5.6 (d, *J* = 2.4 Hz, 1H), 3.45 (s, 3H), 2.30 (s, 3H) ppm. ^13^C NMR (75 MHz, DMSO-*d_6_*): *δ* = 165.9, 151.8, 149.9, 141.9, 132.1, 129.9, 129.5, 129.1, 128.2, 98.1, 51.7, 51.4, 18.2 ppm. The enantiomeric excess was determined by HPLC (Daicel Chiralpak AD-H, hexane/isopropanol = 85:15, flow rate 1.0 mL/min, *λ* = 254 nm), t_Rmajor_ = 12.148 min, t_Rminor_ = 14.796 min.

**5-Methoxycarbonyl-6-methyl-4-(3-nitrophenyl)-3,4-dihydropyrimidin-2(1H)-one (10f):** ^[17]^

^1^H NMR (300 MHz, DMSO-*d_6_*) δ = 9.41 (s, 1H), 8.13 (dd, *J* = 7.4, 1.9 Hz, 1H), 8.08 (s, 1H), 7.94 (s, 1H), 7.69 – 7.64 (m, 2H), 5.30 (d, *J* = 3.3 Hz, 1H), 3.54 (s, 3H), 2.28 (s, 3H) ppm. ^13^C NMR (75 MHz, DMSO-*d_6_*) δ = 166.01, 152.21, 150.10, 148.24, 147.12, 133.33, 130.67, 122.82, 121.30, 98.46, 53.73, 51.35, 18.31 ppm. The enantiomeric excess was determined by HPLC (Daicel Chiralpak AD-H, hexane/isopropanol = 85:15, flow rate 1.0 mL/min, *λ* = 254 nm), t_Rmajor_ = 17.795 min, t_Rminor_ = 24.397 min.

**5-Ethoxycarbonyl-6-methyl-4-phenyl-3,4-dihydropyrimidin-2(1H)-one (10g):** [[^18^](#_ENREF_6)]

^1^H NMR (300 MHz, DMSO-*d_6_*): *δ* = 9.22 (s, 1H), 7.95-7.97 (m, 1H), 7.76 (s, 1H), 7.48-7.53 (m, 2H), 7.30-7.35 (m, 2H), 5.15 (d, *J* = 2.7 Hz, 1H), 3.98 (q, *J* = 7.0 Hz, 2H), 2.26 (s, 3H), 1.09 (t, *J* = 7.0 Hz, 3 H) ppm. ^13^C NMR (75 MHz, DMSO-*d_6_*): *δ =* 167.7, 165.7, 152.5, 148.8, 148.7, 145.3, 133.2, 129.6, 128.9, 128.5, 127.6, 126.6, 99.7, 59.6, 54.4, 18.2, 18.1, 14.5 ppm.

**Coumarin derivatives 13a-g (Table 5, entries 1-7)**

**3-benzoyl-6-nitro-2*H*-chromen-2-one (13a):** [[^19^](#_ENREF_6)]

^1^H NMR (300 MHz, CDCl_3_): *δ* = 8.52-8.49 (m, 2H), 8.12 (s, 1H), 7.90-7.88 (m, 2H), 7.69-7.64 (m, 1H), 7.57-7.49 (m, 3H) ppm. ^13^C NMR (75 MHz, CDCl_3_): *δ* = 190.3, 157.8, 156.2, 144.3, 143.4, 135.4, 134.3, 129.6, 129.1, 128.8, 127.9, 124.8, 118.2 ppm.

**3-Benzoyl-6-chloro-2*H*-chromen-2-one (13b):** [[^20^](#_ENREF_6)]

^1^H NMR (300 MHz, CDCl_3_): *δ* = 7.98 (s, 1H), 7.87-7.85 (m, 2H), 7.64-7.57 (m, 3H), 7.50-7.45 (m, 2H), 7.36-7.33 (m, 1H) ppm. ^13^C NMR (75 MHz, CDCl_3_): *δ* = 191.1, 157.8, 153.0, 143.9, 135.8, 134.0, 133.4, 130.2, 129.5, 128.6, 128.2, 128.0, 119.1, 118.3 ppm.

**3-Benzoyl-6-methoxy-2*H*-chromen-2-one (13c):** [[^20^](#_ENREF_6)]

^1^H NMR (300 MHz, CDCl_3_) δ = 8.03 (s, 1H), 7.87 (d, *J* = 7.5 Hz, 2H), 7.60 (m, 1H), 7.47 (m, 2H), 7.30 (m, 1H), 7.22 (m, 1H), 7.01 (s, 1H), 3.85 (s, 3H). ^13^C NMR (75 MHz, CDCl_3_) δ = 191.8, 158.6, 156.3, 149.2, 145.3, 133.8, 129.5, 128.5, 127.0, 121.7, 118.4, 117.9, 110.6, 55.9.

**2-benzoyl-3*H*-benzo[f]chromen-3-one (13d):** [[^21^](#_ENREF_6)]

^1^H NMR (300 MHz, DMSO) δ 9.42 (s, 1H), 8.80 (d, *J* = 8.0 Hz, 1H), 8.51 (d, *J* = 8.9 Hz, 1H), 8.29 (d, *J* = 7.7 Hz, 1H), 8.19 (d, *J* = 7.2 Hz, 2H), 7.89 (m, 4H), 7.75 (m, 2H). ^13^C NMR (75 MHz, DMSO) δ = 154.8, 141.5, 136.5, 135.3, 134.3, 130.3, 130.0, 129.5, 129.3, 129.1, 126.7, 126.0, 122.9, 117.1, 113.0.

**3-Benzoyl-2*H*-chromen-2-one (13e):** [[^20^](#_ENREF_6)]

^1^H NMR (300 MHz, CDCl_3_): *δ* = 8.06 (s, 1H), 7.88-7.85 (m, 2H), 7.65-7.57 (m, 3H), 7.48-7.43 (m, 2H), 7.38-7.31 (m, 2H) ppm. ^13^C NMR (75 MHz, CDCl_3_): *δ* = 191.6, 158.4, 154.6, 154.5, 136.1, 133.8, 133.6, 129.5, 129.3, 128.6, 126.7, 125.0, 118.1, 116.8 ppm.

**Ethyl 2-oxo-2*H*-chromene-3-carboxylate (13f):** [[^20^](#_ENREF_6)]

^1^H NMR (300 MHz, CDCl_3_): *δ* = 8.55 (s, 1H), 7.69-7.62 (m, 2H), 7.38-7.33 (m, 3H), 4.46-4.39 (m, 2H), 1.44-1.39 (m, 3H) ppm. ^13^C NMR (75 MHz, CDCl_3_): *δ* = 163.0, 156.7, 155.1, 148.6, 134.3, 129.5, 124.8, 118.2, 117.8, 116.7, 61.9, 14.2 ppm.

**3-Acetyl-2*H*-chromen-2-one (13g):** [[^20^](#_ENREF_6)]

^1^H NMR (300 MHz, CDCl_3_): *δ* = 8.51 (s, 1H), 7.69-7.67 (m, 2H), 7.38 (d, *J* = 7.1 Hz, 2H), 2.72 (s, 3H) ppm. ^13^C NMR (75 MHz, CDCl_3_): *δ* = 195.3, 159.1, 155.2, 147.4, 134.3, 130.2, 124.9, 124.4, 118.1, 116.5 ppm.

**Aza-Diels-Alder products 15a-e and 16a-e (Table 6, entries 1-5)**

**3-*endo*-(3-Fluorophenyl)-2-(4-methoxyphenyl)-2-azabicyclo-[2.2.2]octan-5-one (15a):** ^[22]^

^1^H NMR (400 MHz, CDCl_3_): δ = 6.94-7.30 (m, 4H), 6.80 (d, *J* = 9.1 Hz, 2H), 6.63 (d, *J* = 9.2 Hz, 2H), 4.59 (s, 1H), 4.45 (s, 1H), 3.74 (s, 3H), 2.75 (m, 2H), 2.49 (m, 1H), 2.25 (m, 1H), 2.15(m, 1H), 2.04 (m, 1H), 1.76 (m, 1H) ppm. ^13^C NMR (101 MHz, CDCl_3_): δ = 211.62, 164.56, 162.11, 152.32, 145.30, 145.24, 142.17, 130.62, 130.54, 121.36, 114.79, 114.76, 114.59, 114.38, 112.96, 112.74, 65.73, 55.65, 52.10, 49.49, 46.09, 22.58, 22.27 ppm.

**3-*exo*-(3-Fluorophenyl)-2-(4-methoxyphenyl)-2-azabicyclo-[2.2.2]octan-5-one (16a):** ^[22]^

^1^H NMR (400 MHz, CDCl_3_): δ = 6.99-7.35 (m, 4H), 6.75 (d, *J* = 9.1 Hz, 2H), 6.54 (d, *J* = 9.1 Hz, 2H), 4.70 (s, 1H), 4.45 (s, 1H), 3.71 (s, 3H), 2.75 (m, 1H), 2.65 (m, 1H), 2.36 (m, 1H), 2.25 (m, 1H), 1.90 (m, 1H), 1.68 (m, 2H) ppm. ^13^C NMR (101 MHz, CDCl_3_): δ = 213.47, 164.63, 162.18, 152.23, 143.65, 143.59, 142.38, 130.46, 130.38, 121.82, 121.79, 114.88, 114.47, 114.38, 114.26, 113.47, 113.25, 62.29, 55.66, 50.80, 48.90, 41.89, 26.28, 16.34 ppm.

**3-*endo*-(4-Fluorophenyl)-2-(4-methoxyphenyl)-2-azabicyclo-[2.2.2]octan-5-one (15b):** ^[22]^

^1^H NMR (400 MHz, CDCl_3_): δ = 7.26 (d, *J* = 8.6 Hz, 2H), 6.99 (d, *J* = 8.6 Hz, 2H), 6.77 (d, *J* = 9.1 Hz, 2H), 6.60 (d, *J* = 9.1 Hz, 2H), 4.56 (s, 1H), 4.43 (s, 1H), 3.72 (s, 3H), 2.73 (m, 2H), 2.49 (m, 1H), 2.25 (m, 1H), 2.15 (m, 1H), 2.02 (m, 1H), 1.74 (m, 1H) ppm. ^13^C NMR (101 MHz, CDCl_3_): δ = 211.82, 163.30, 160.86, 152.28, 142.24, 137.94, 137.92, 127.39, 127.31, 115.93, 115.72, 114.79, 114.77, 65.53, 55.66, 52.32, 49.57, 46.12, 22.51, 22.31 ppm.

**3-*exo*-(4-Fluorophenyl) -2-(4-methoxyphenyl)-2-azabicyclo-[2.2.2]octan-5-one (16b):** ^[22]^

^1^H NMR (400 MHz, CDCl_3_): δ = 7.38 (m, 2H), 7.06 (m, 2H), 6.74 (d, *J* = 9.1 Hz, 2H), 6.54 (d, *J* = 9.1 Hz, 2H), 4.68 (s, 1H), 4.43 (s, 1H), 3.70 (s, 3H), 2.77 (m, 1H), 2.62 (m, 1H), 2.38 (m, 1H), 2.23 (m, 1H), 1.89 (m, 1H), 1.68 (m, 2H) ppm. ^13^C NMR (101 MHz, CDCl_3_): δ = 213.71, 163.33, 160.89, 152.16, 142.45, 136.03, 136.00, 127.85, 127.77, 115.83, 115.62, 114.87, 114.36, 62.03, 55.67, 51.06, 48.96, 41.91, 26.32, 16.25 ppm.

**3-*endo*-(4-Fluorophenyl)-2-phenyl-2-azabicyclo-[2.2.2]octan-5-one (15c):** ^[22]^

^1^H NMR (400 MHz, CDCl_3_): δ = 7.21-7.28 (m, 4H), 7.02 (d, *J* = 8.4 Hz, 2H), 6.78 (m, 1H), 6.67 (d, *J* = 8.4 Hz, 2H), 4.66 (s, 1H), 4.57 (s, 1H), 2.75 (m, 2H), 2.49 (m, 1H), 2.29 (m, 1H), 2.10 (m, 2H), 1.77 (m, 1H) ppm. ^13^C NMR (101 MHz, CDCl_3_): δ = 211.58, 163.33, 160.89, 147.96, 137.53, 129.36, 127.36, 127.28(2C), 118.03, 116.01, 115.79, 113.45, 65.26, 52.29, 48.69, 45.90, 22.69, 22.47 ppm.

**3-*exo*-(4-Fluorophenyl)-2-phenyl-2-azabicyclo-[2.2.2]octan-5-one** **(16c):**

^1^H NMR (400 MHz, CDCl_3_): δ = 7.18-7.40 (m, 4H), 7.09 (t, *J* = 8.5 Hz, 2H), 6.75 (m, 1H), 6.60 (d, *J* = 8.5 Hz, 2H), 4.76 (s, 1H), 4.57 (s, 1H), 2.74 (m, 2H), 2.44 (m, 1H), 2.24 (m, 1H), 1.94 (m, 1H), 1.70 (m, 2H) ppm. ^13^C NMR (101 MHz, CDCl_3_): δ = 213.33, 163.36, 160.92, 148.01, 135.66, 135.63, 129.40, 127.76, 127.68, 117.95, 115.89, 115.67, 113.14, 61.79, 51.00, 48.22, 42.25, 26.01, 16.26 ppm.

**3-*endo*-(3-Chlorophenyl)-2-(4-methoxyphenyl)-2-azabicyclo-[2.2.2]octan-5-one (15d):** ^[23]^

^1^H NMR (400 MHz, CDCl_3_): δ = 7.21-7.31 (m 4H), 6.80 (d, *J* = 9.2 Hz, 2H), 6.62 (d, *J* = 9.2 Hz, 2H), 4.54 (s, 1H), 4.45 (s, 1H), 3.74 (s, 3H), 2.77 (m, 2H), 2.49 (m, 1H), 2.27 (m, 1H), 2.14 (m, 1H), 2.05 (m, 1H), 1.76 (m，1H) ppm. ^13^C NMR (101 MHz, CDCl_3_): δ = 211.50, 152.37, 144.60, 142.15, 134.91, 130.30, 127.80, 126.01, 123.97, 114.93(2C), 114.81(2C), 65.77, 55.67, 52.10, 49.52, 46.13, 22.61, 22.27 ppm.

**3-*exo*-(3-Chlorophenyl)-2-(4-methoxyphenyl)-2-azabicyclo-[2.2.2]octan-5-one (16d):** ^[23]^

^1^H NMR (400 MHz, CDCl_3_): δ = 7.25-7.43 (m, 4H), 6.75 (d, *J* = 9.1 Hz, 2H), 6.54 (d, *J* = 9.1 Hz, 2H), 4.66 (s, 1H), 4.42 (s, 1H), 3.71 (s, 3H), 2.75 (dd, *J* = 18.8, 3.0 Hz, 1H), 2.63 (m, 1H), 2.40 (dd, *J* = 18.8 ,1.8 Hz, 1H), 2.36 (m, 1H), 1.89 (m, 1H), 1.69 (m, 2H) ppm. ^13^C NMR (101 MHz, CDCl_3_): δ = 213.35, 152.27, 142.96, 142.37, 134.93, 130.18, 127.71, 126.43, 124.47, 114.91(2C), 114.42(2C), 62.37, 55.67, 50.77, 48.89, 41.87, 26.29, 16.31 ppm.

**3-*endo*-(4-Chlorophenyl)-2-(4-methoxy phenyl)-2-azabicyclo-[2.2.2]octan-5-one (15e):** ^[23]^

^1^H NMR (400 MHz, CDCl_3_): δ = 7.26 (m, 4H), 6.77 (m, 2H), 6.59 (m, 2H), 4.55 (s, 1H), 4.43 (s, 1H), 3.72 (s, 3H), 2.72 (m, 2H), 2.47 (dd, *J* = 18.9, 2.4 Hz, 1H), 2.25 (d, 1H), 2.13 (d, 1H), 2.03 (m, 1H), 1.75 (m, 1H) ppm ^13^C NMR (101 MHz, CDCl_3_): δ = 211.70, 152.32, 142.15, 140.85, 133.17, 129.14(2C), 127.21(2C), 114.80(2C), 114.78(2C), 65.58, 55.67, 52.18, 49.56, 46.13, 22.52, 22.32 ppm.

**3-*exo*-(4-Chlorophenyl)-2-(4-methoxyphenyl)-2-azabicyclo-[2.2.2]octan-5-one (16e):** ^[23]^

^1^H NMR (400 MHz, CDCl_3_): δ = 7.35 (s, 4H), 6.74 (d, *J* = 9.1 Hz, 2H), 6.53 (d, *J* = 9.1 Hz, 2H), 4.66 (d, *J* = 2.3 Hz, 1H), 4.41 (s, 1H), 3.70 (s, 3H), 2.74 (d, 1H), 2.62 (d, *J* = 2.8 Hz, 1H), 2.36 (m, 1H), 2.22 (m, 1H), 1.88 (m, 1H), 1.67 (m, 2H). ^13^C NMR (101 MHz, CDCl_3_): δ = 213.51, 152.21, 142.36 , 139.06 , 133.10 , 129.03(2C) , 127.70(2C) , 114.89(2C) , 114.37(2C) , 62.12 , 55.68 , 50.87 , 48.94 , 41.92 , 26.32 , 16.27 ppm.

**References**

[1] W.-W. Zheng, Y.-H. Hsieh, Y.-C. Chiu, S.-J. Cai, C.-L. Cheng, C. Chen, Organic functionalization of ultradispersed nanodiamond: synthesis and applications. *J. Mater. Chem.* **19**, 8432-8441 (2009).

[2] H. Yang, R. G. Carter, N-(p-dodecylphenylsulfonyl)-2-pyrrolidinecarboxamide: a practical proline mimetic for facilitating enantioselective aldol reactions. *Org. Lett.* **10**, 4649-4652 (2008).

[3] Y. Qian, X. Zheng, Y. Wang, A green and efficient asymmetric aldol reaction catalyzed by a chiral anion modified ionic liquid. *Eur. J. Org. Chem.* **2010**, 3672-3677 (2010).

[4] F. Chen, S. Huang, H. Zhang, F. Liu, Y. Peng, Proline-based dipeptides with two amide units as organocatalyst for the asymmetric aldol reaction of cyclohexanone with aldehydes. *Tetrahedron* **64**, 9585-9591 (2008).

[5] C. Wu, X. Fu, S. Li, A highly efficient, large-scale, asymmetric direct aldol reaction employing simple threonine derivatives as recoverable organocatalysts in the presence of water. *Eur. J. Org. Chem.* **2011**, 1291-1299 (2011).

[6] Z. Guan, J.-P. Fu, Y.-H. He, Biocatalytic promiscuity: lipase-catalyzed asymmetric aldol reaction of heterocyclic ketones with aldehydes. *Tetrahedron Lett.* **53**, 4959-4961 (2012).

[7] D. Almaşi, D. A. Alonso, C. Nájera, Prolinamides versus prolinethioamides as recyclable catalysts in the enantioselective solvent-free inter- and intramolecular aldol reactions. *Adv. Synth. Catal.* **350**, 2467-2472 (2008).

[8] Y.-J. An, C.-C. Wang, Z.-P. Liu, J.-C. Tao, Isosteviol-proline conjugates as highly efficient amphiphilic organocatalysts for asymmetric three-component Mannich reactions in the presence of water. *Helv. Chim. Acta* **95,** 43-51 (2012).

[9] Q. X. Guo, H. Liu, C. Guo, S. W. Luo, Y. Gu, L. Z. Gong, Chiral Brønsted acid-catalyzed direct asymmetric Mannich reaction. *J. Am. Chem. Soc.* **129**, 3790-3791 (2007).

[[10]](#_ENREF_1) F. Niu, J. Wu, L. Zhang, P. Li, J. Zhu, Z. Wu, C. Wang, W. Song, Hydroxyl group rich C60 fullerenol: an excellent hydrogen bond catalyst with superb activity, selectivity, and stability. *ACS Catalysis* **1,** 1158-1161 (2011).

[[11]](#_ENREF_1) Y. Song, H. Jing, B. Li, D. Bai, Crown ether complex cation ionic liquids: preparation and applications in organic reactions. *Chem.Eur. J.* **17,** 8731-8738, (2011).

[[12]](#_ENREF_1) T. Nitabaru, A. Nojiri, M. Kobayashi, N. Kumagai, M. Shibasaki, *anti*-Selective catalytic asymmetric nitroaldol reaction via a heterobimetallic heterogeneous catalyst. *J. Am. Chem. Soc.* **131**, 13860-13869 (2009).

[13] Y. Wang, H. Yang, J. Yu, Z. Miao, R. Chen, Highly enantioselective Biginelli reaction promoted by chiral bifunctional primary amine-thiourea catalysts: asymmetric synthesis of dihydropyrimidines. *Adv. Synth. Catal.* **351**, 3057-3062 (2009).

[14] H. Adibi, H. A. Samimi, M. Beygzadeh, Iron(III) trifluoroacetate and trifluoromethanesulfonate: recyclable Lewis acid catalysts for one-pot synthesis of 3,4-dihydropyrimidinones or their sulfur analogues and 1,4-dihydropyridines via solvent-free Biginelli and Hantzsch condensation protocols. *Catal. Commun.* **8**, 2119-2124 (2007).

[15] D. Ding, C.-G. Zhao, Primary amine catalyzed Biginelli reaction for the enantioselective synthesis of 3,4-dihydropyrimidin-2(1H)-ones. *Eur. J. Org. Chem.* **2010,** 3802-3005 (2010).

[16] Y.-F. Cai, H.-M. Yang, L. Li, K.-Z. Jiang, G.-Q. Lai, J.-X. Jiang, L.-W. Xu, Cooperative and enantioselective NbCl_5_/primary amine catalyzed Biginelli reaction. *Eur. J. Org. Chem.* **2010,** 4986-4990 (2010).

[17] S. S. Bahekar, S. A. Kotharkar, D. B. Shinde, One-pot construction of dihydropyrimidinones in ionic liquids. *Mendeleev Commun.* **14**, 210-212 (2004).

[18] H. Karade, M. Sathe, M. Kaushik, Synthesis of 4-aryl substituted 3,4-dihydropyrimidinones using silica-chloride under solvent free conditions. *Molecules* **12**, 1341-1351 (2007).

[19] Y.-J. Jang, S.-E. Syu, Y.-J. Chen, M.-C. Yang, W. Lin, Syntheses of furo[3,4-c]coumarins and related furyl coumarin derivatives via intramolecular Wittig reactions. *Org. Biomol. Chem.* **10,** 843-847 (2012).

[20] H.-J. Yuan, M. Wang, Y.-J. Liu, Q. Liu, Copper(II)-catalyzed C-C bond-forming reactions of α-electron-withdrawing group-substituted ketene S,S-acetals with carbonyl compounds and a facile synthesis of coumarins. *Adv. Synth. Catal.* **351**, 112-116, (2009).

[21] X. Tang, A. J. Blake, W. Lewis, S. Woodward, Asymmetric conjugate additions to 1,1′-diactivated cyclic enones-a comparative study. *Tetrahedron: Asymmetry* **20**, 1881-1891 (2009).

[22] Y.-H. He, W. Hu, Z. Guan, Enzyme-catalyzed direct three-component aza-Diels–Alder reaction using hen egg white lysozyme. *J. Org.Chem.* **77**, 200–207 (2012).

[23] U. Costantino, F. Fringuelli, M. Orrù, M. Nocchetti, O. Piermatti, F. Pizzo, Direct aza-Diels-Alder reaction in water catalyzed by layered
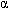
-zirconium hydrogen phosphate and sodium dodecyl sulfate. *Eur. J. Org. Chem*. **8**, 1214–1220 (2009).
