# Supplementary material for: Earthworm Is a Versatile and Sustainable Biocatalyst for Organic Synthesis
Source: PLoS One. 2014 Aug 22;9(8):e105284. doi: 10.1371/journal.pone.0105284 (PMC4141794; doi:10.1371/journal.pone.0105284)
Supplement: Materials S1 — The materials and general methods. (DOC) [file pone.0105284.s006.doc]

**Supporting Information Materials S1**

Earthworm is a versatile and sustainable biocatalyst for organic synthesis

Zhi Guan, Yan-Li Chen, Yi Yuan, Jian Song, Da-Cheng Yang, Yang Xue, Yan-Hong He*

School of Chemistry and Chemical Engineering, Southwest University, Chongqing, 400715, P. R. China

Fax: (+86)23-68254091; Email: heyh@swu.edu.cn

**Materials S1** The materials and general methods.

**1 Materials**

Live earthworms (*Eisenia foetida,* the common Chinese name: “Daping II”) were purchased from Tianjin Cheng Gong earthworm farm (Tianjin, China). All reagents were purchased from commercial suppliers and used without further purification.

**2 General methods**

All reactions were monitored by thin-layer chromatography (TLC) with Haiyang GF254 silica gel plates. Flash column chromatography was carried out using 100-200 mesh silica gel at increased pressure. The NMR spectra were recorded on Bruker AMX-300MHz and Bruker AMX-400MHz spectrometers. Chemical shifts (δ) are expressed in ppm with TMS as internal standard, and coupling constants (*J*) are reported in Hz. The enantiomeric excess (ee) of products was determined by chiral HPLC analysis performed using Chiralpak AD-H, AS-H, and Chiralcel OD-H columns. Relative and absolute configurations of the products were determined by comparing ^1^H NMR, ^13^C NMR and chiral HPLC analysis.with those of reported compounds (their *syn/anti* configurations and absolute configurations have been reported previously). The preparation of racemic mixtures of aldol products: aldehyde (0.50 mmol), ketone (7.50 mmol), EtOH (1.0 mL), and saturated NaHCO_3_ (2-3 drops) stirred at r.t. overnight. The preparation of racemic mixtures of Mannich products: aldehyde (0.50 mmol), arylamine (0.55 mmol), cyclohexanone (5.00 mmol), MeCN (1.0 mL), and 0.1 M H_2_SO_4_ (2-3 drops) stirred at r.t. overnight. The preparation of racemic mixtures of Biginelli products: aldehyde (0.50 mmol), urea (1.00 mmol), acetoacetate (1.00 mmol), and acidic ionic liquid 1-butylsulfonic-3-methylimidazolium hydrogensulfate ([BSO_3_HMIm]HSO_4_, 0.5 mL) stirred at r.t. overnight.
