# Supplementary material for: Earthworm Is a Versatile and Sustainable Biocatalyst for Organic Synthesis
Source: PLoS One. 2014 Aug 22;9(8):e105284. doi: 10.1371/journal.pone.0105284 (PMC4141794; doi:10.1371/journal.pone.0105284)
Supplement: Spectra S1 — 1H and 13C NMR-spectra and HPLC chart. (DOC) [file pone.0105284.s007.doc]

**Supporting Information Spectra S1**

Earthworm is a versatile and sustainable biocatalyst for organic synthesis

Zhi Guan, Yan-Li Chen, Yi Yuan, Jian Song, Da-Cheng Yang, Yang Xue, Yan-Hong He*

School of Chemistry and Chemical Engineering, Southwest University, Chongqing, 400715, P. R. China

Fax: (+86)23-68254091; Email: heyh@swu.edu.cn

**Spectra S1 ^1^H and ^13^C NMR-spectra and HPLC chart**

**Aldol products**

**2-(Hydroxy-(3-Cyanophenyl-phenyl)methyl) cyclohexan-1-one (****3a):** [[^1^](#_ENREF_2)]


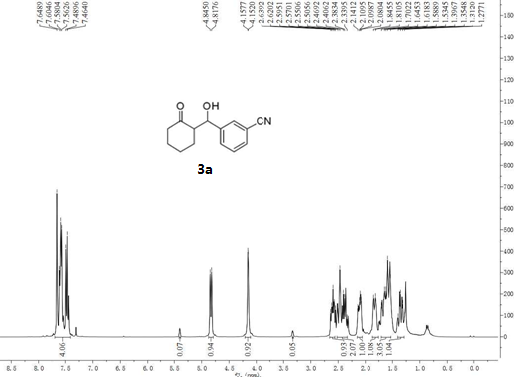


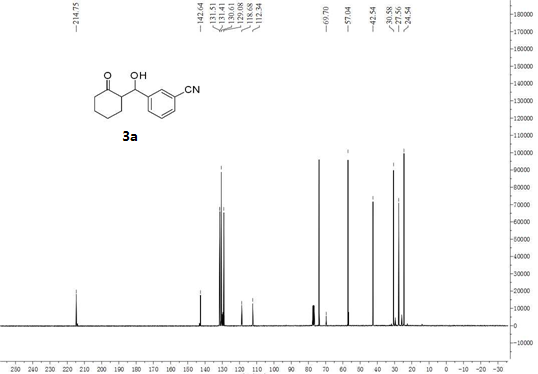


**3a (Racemic)**


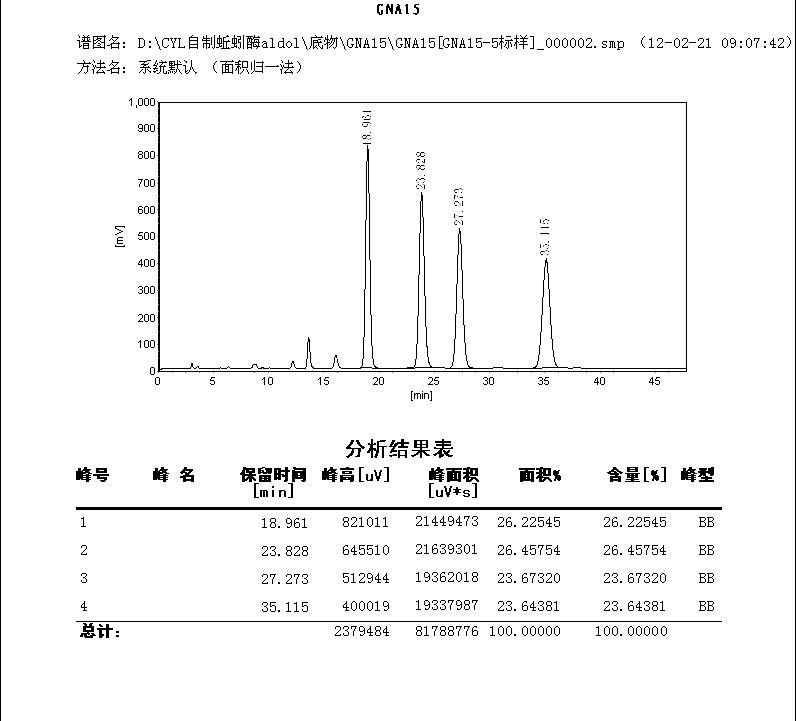


DEFAULT REPORT

| Peak # | Time [min] | Height [μv] | Area [μv.s] | Area [%] |
| --- | --- | --- | --- | --- |
| 1 | 18.961 | 821011 | 21449473 | 26.22545 |
| 2 | 23.828 | 645510 | 21639301 | 26.45754 |
| 3 | 27.273 | 512944 | 19362018 | 23.67320 |
| 4 | 35.115 | 400019 | 19337987 | 23.64381 |
| [Sum](http://www.nciku.cn/search/en/sum) |  | 2379484 | 81788776 | 100.00000 |

**3a (Chiral)**


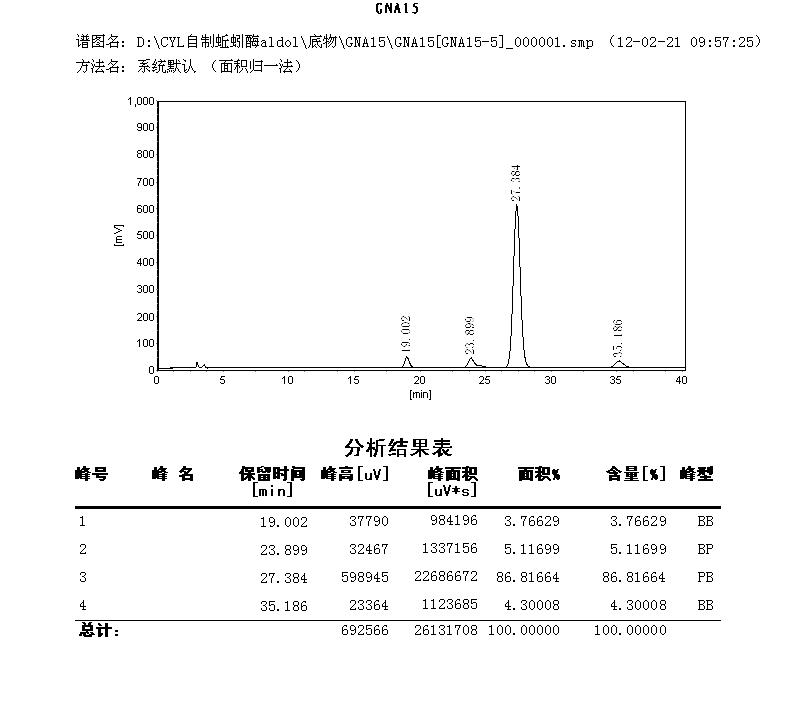
 DEFAULT REPORT

| Peak # | Time [min] | Height [μv] | Area [μv.s] | Area [%] |
| --- | --- | --- | --- | --- |
| 1 | 19.002 | 37790 | 984196 | 3.76629 |
| 2 | 23.899 | 32467 | 1337156 | 5.11699 |
| 3 | 27.384 | 598945 | 22686672 | 86.81664 |
| 4 | 35.186 | 23364 | 1123685 | 4.30008 |
| [Sum](http://www.nciku.cn/search/en/sum) |  | 692566 | 81788776 | 100.00000 |

**2-(Hydroxy-(4-chlorophenyl-phenyl)methyl)cyclohexan-1-one (****3b):** [[^2^](#_ENREF_3)^, 3]^


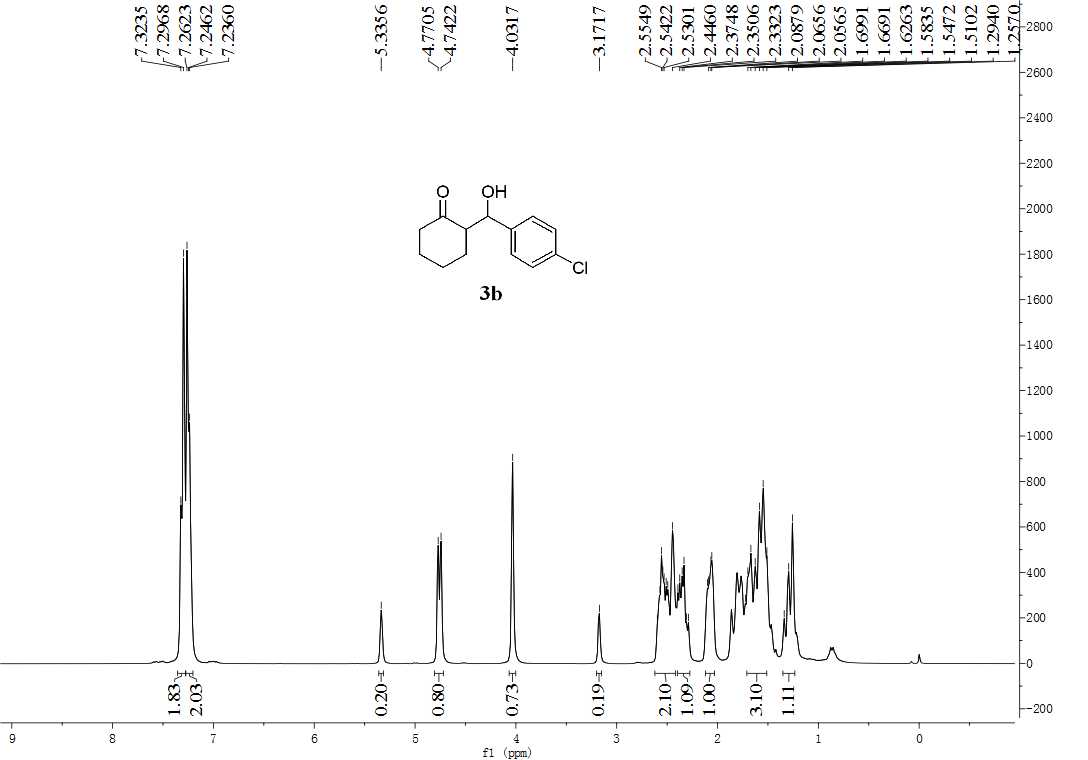


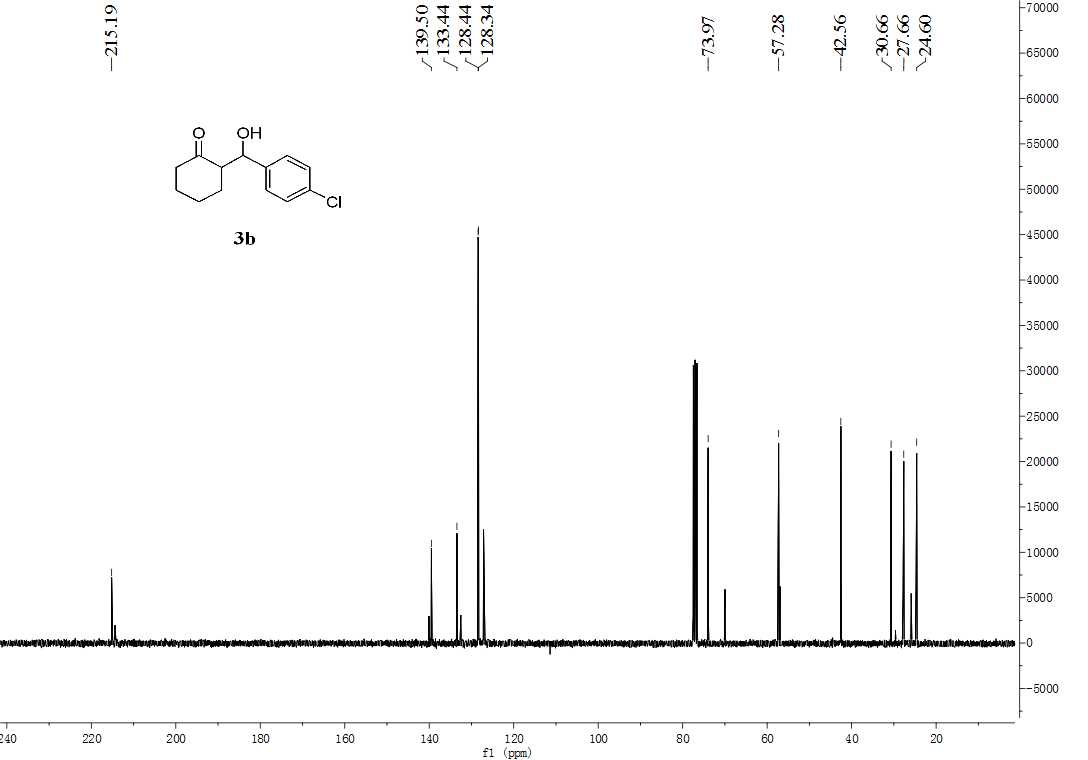


**3b (Racemic)**


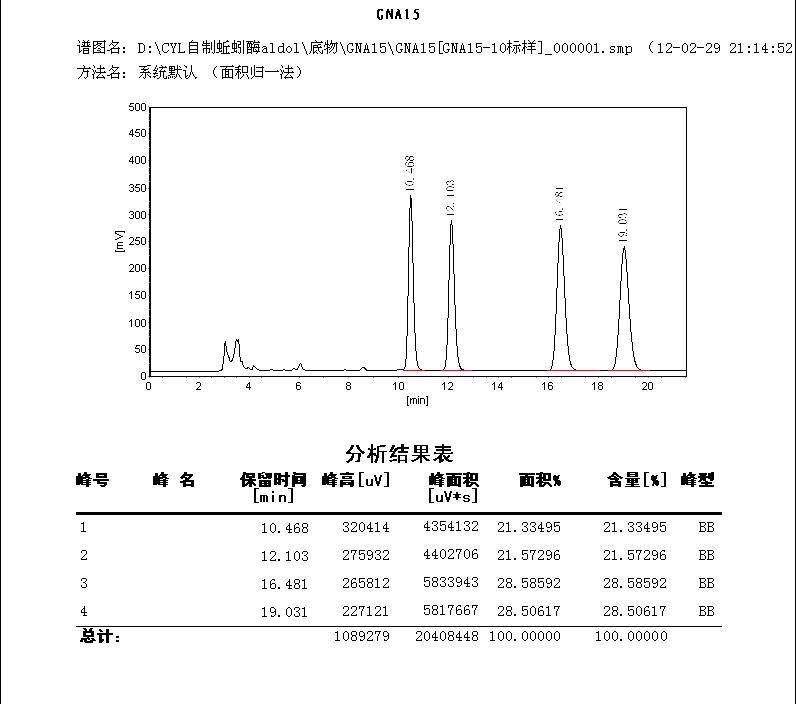


DEFAULT REPORT

| Peak # | Time [min] | Height [μv] | Area [μv.s] | Area [%] |
| --- | --- | --- | --- | --- |
| 1 | 10.468 | 320414 | 4354132 | 21.33495 |
| 2 | 12.103 | 275932 | 4402706 | 21.57296 |
| 3 | 16.481 | 265812 | 5833943 | 28.58592 |
| 4 | 19.031 | 227121 | 5817667 | 28.50617 |
| [Sum](http://www.nciku.cn/search/en/sum) |  | 1088279 | 20408448 | 100.00000 |

**3b (Chiral)**


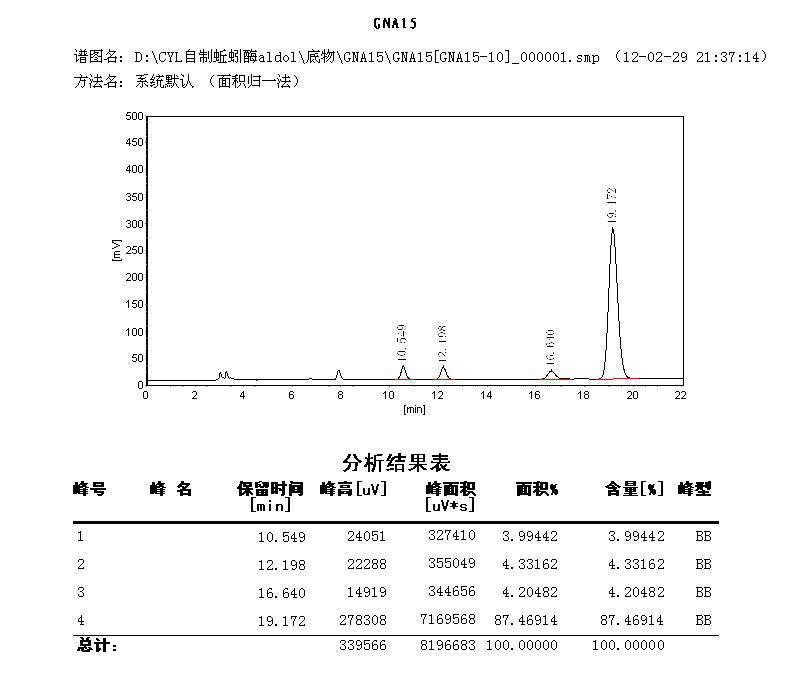


DEFAULT REPORT

| Peak # | Time [min] | Height [μv] | Area [μv.s] | Area [%] |
| --- | --- | --- | --- | --- |
| 1 | 10.549 | 24051 | 327410 | 3.99442 |
| 2 | 12.198 | 22288 | 355049 | 4.33162 |
| 3 | 16.640 | 14919 | 344656 | 4.20482 |
| 4 | 19.172 | 278308 | 7169568 | 87.46914 |
| [Sum](http://www.nciku.cn/search/en/sum) |  | 339566 | 8196683 | 100.00000 |

**2-(Hydroxy-(4-trifluoromethyl-phenyl)methyl)cyclohexan-1-one (3c):** [[^3^](#_ENREF_4)]


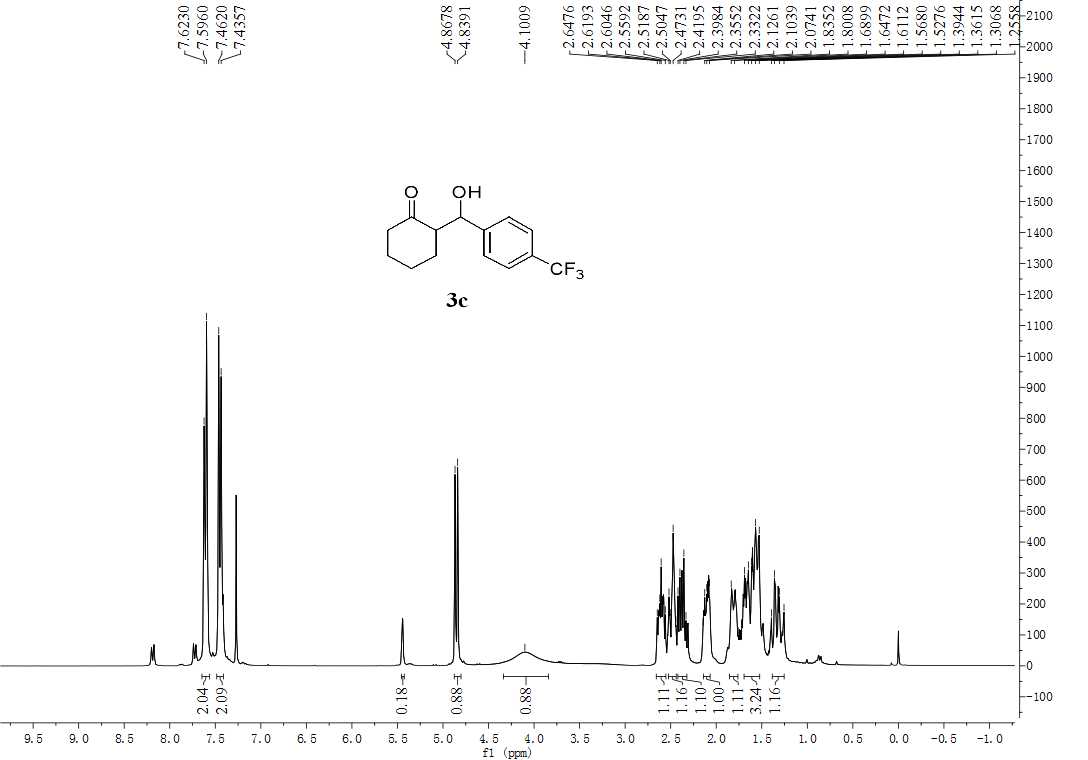


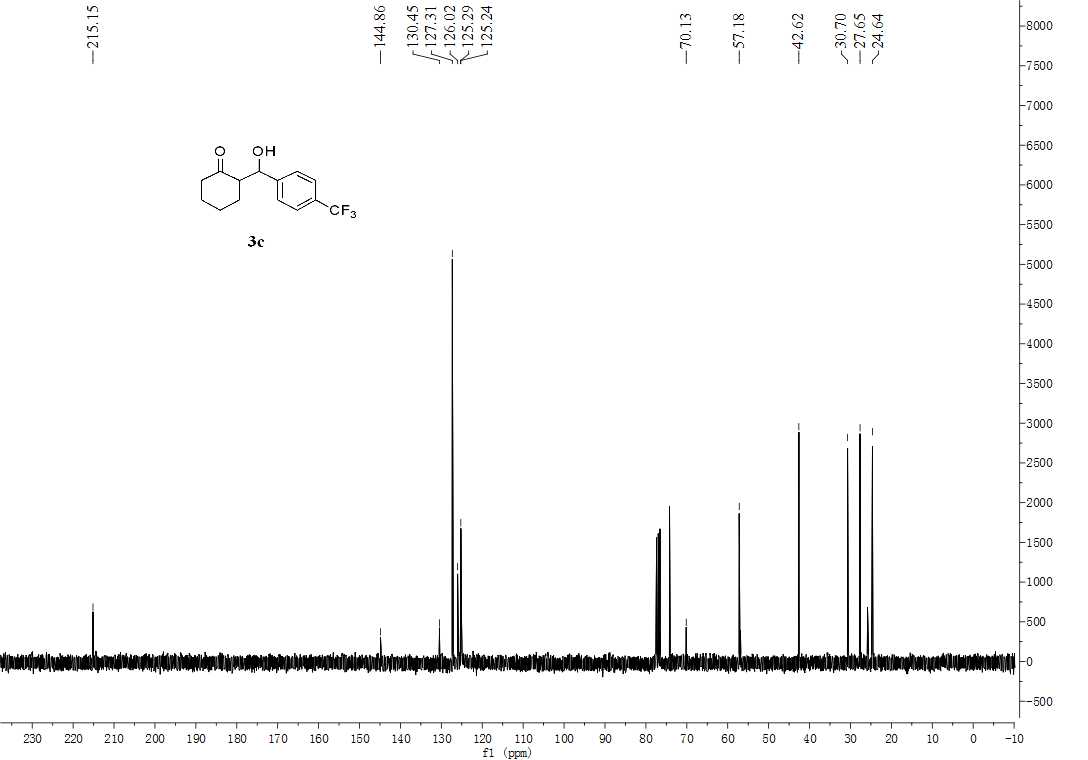


**3c (Racemic)**


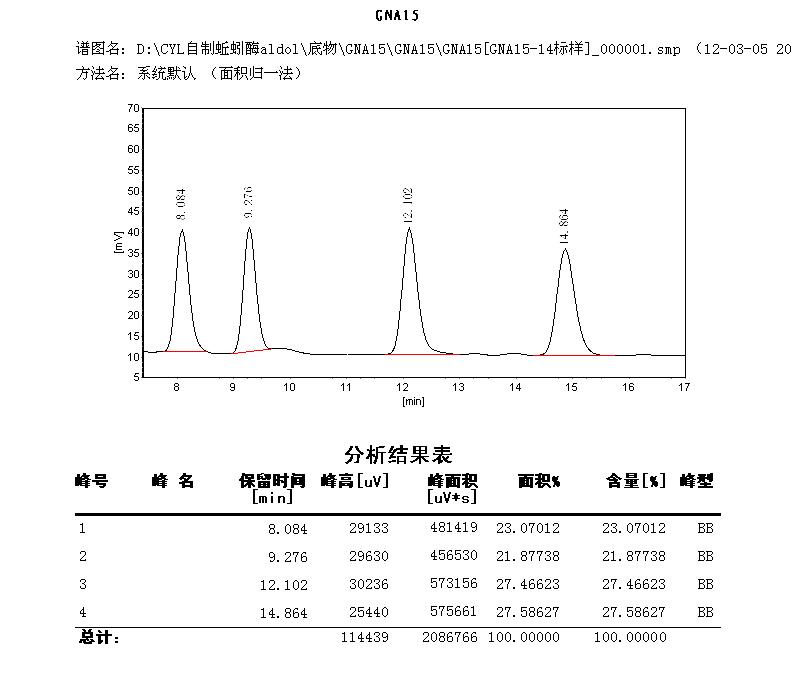


DEFAULT REPORT

| Peak # | Time [min] | Height [μv] | Area [μv.s] | Area [%] |
| --- | --- | --- | --- | --- |
| 1 | 8.084 | 29133 | 481419 | 23.07012 |
| 2 | 9.276 | 29630 | 456530 | 21.87738 |
| 3 | 12.102 | 30236 | 573156 | 27.46623 |
| 4 | 14.864 | 25440 | 575661 | 27.58627 |
| [Sum](http://www.nciku.cn/search/en/sum) |  | 114439 | 2086766 | 100.00000 |

**3c (Chiral)**


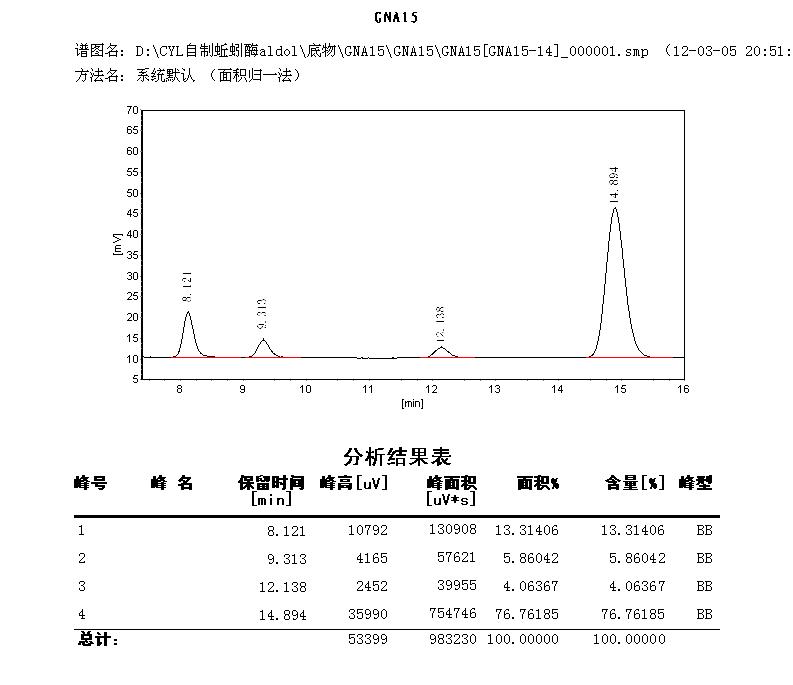
 DEFAULT REPORT

| Peak # | Time [min] | Height [μv] | Area [μv.s] | Area [%] |
| --- | --- | --- | --- | --- |
| 1 | 8.121 | 10792 | 130908 | 13.31406 |
| 2 | 9.313 | 4165 | 57621 | 5.86042 |
| 3 | 12.138 | 2452 | 39955 | 4.06367 |
| 4 | 14.894 | 35990 | 754746 | 76.76185 |
| [Sum](http://www.nciku.cn/search/en/sum) |  | 53399 | 983230 | 100.00000 |

**2-(Hydroxy-(2,4-dichlorophenyl-phenyl)methyl)cyclohexan-1-one (****3d):** [[^2^](#_ENREF_3), [^4^](#_ENREF_5)]


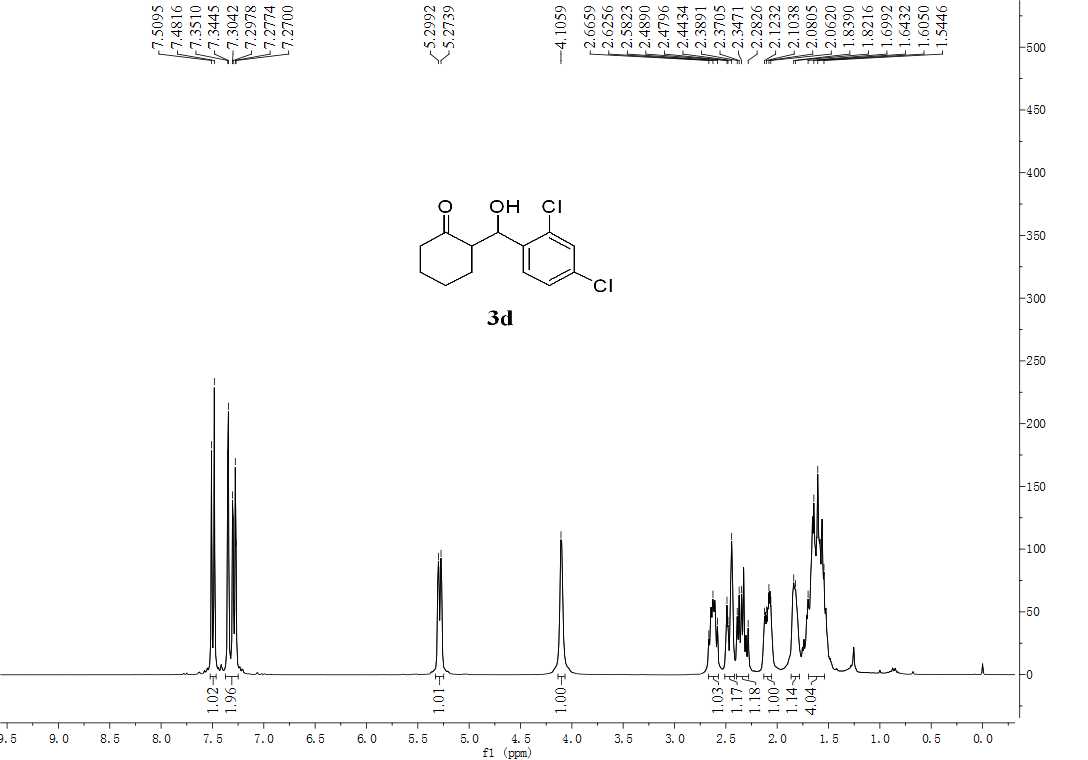


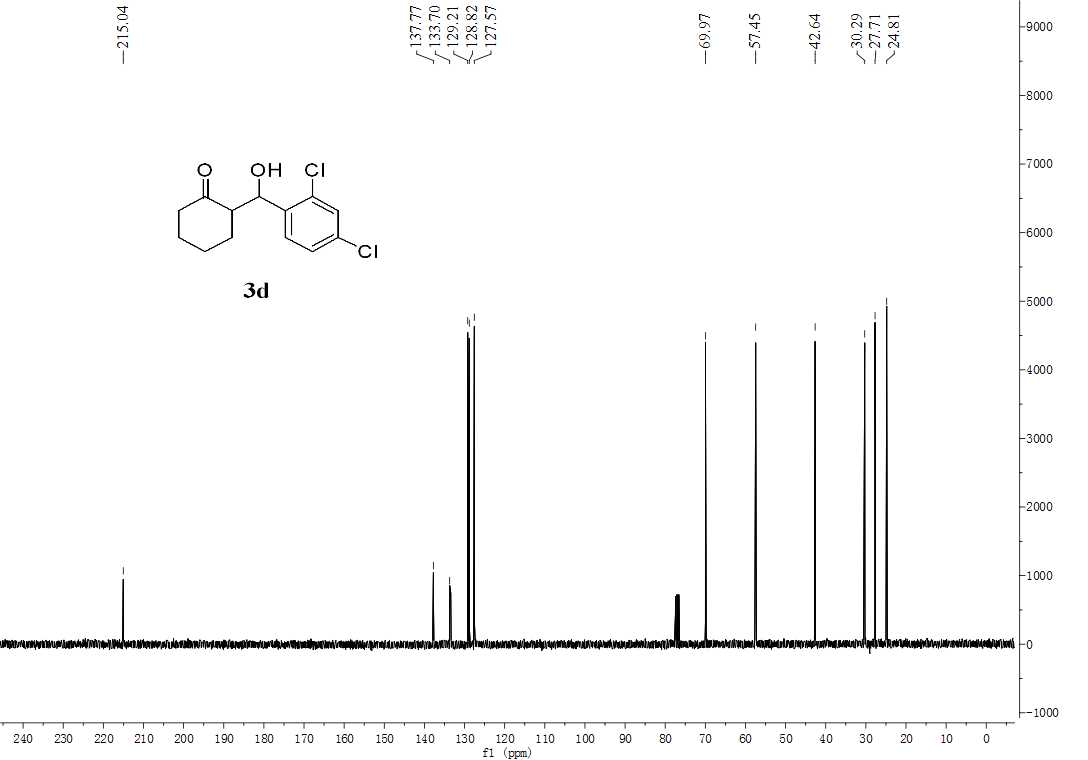


**3d (Racemic)**


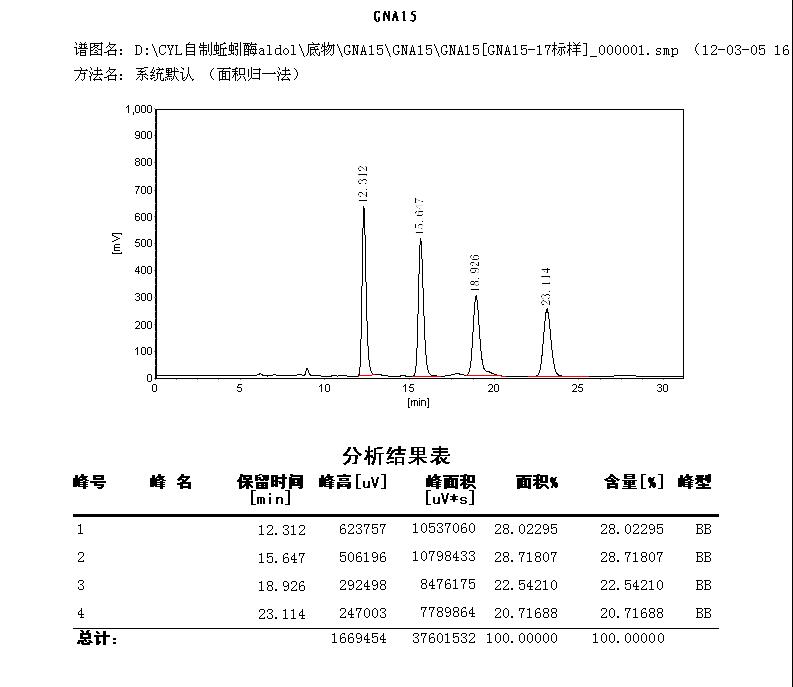


DEFAULT REPORT

| Peak # | Time [min] | Height [μv] | Area [μv.s] | Area [%] |
| --- | --- | --- | --- | --- |
| 1 | 12.312 | 623757 | 10537060 | 28.02295 |
| 2 | 15.647 | 506196 | 10798433 | 28.71807 |
| 3 | 18.926 | 292498 | 8476175 | 22.54210 |
| 4 | 23.114 | 247003 | 7789864 | 20.71688 |
| [Sum](http://www.nciku.cn/search/en/sum) |  | 1669454 | 37601532 | 100.00000 |

**3d (Chiral)**


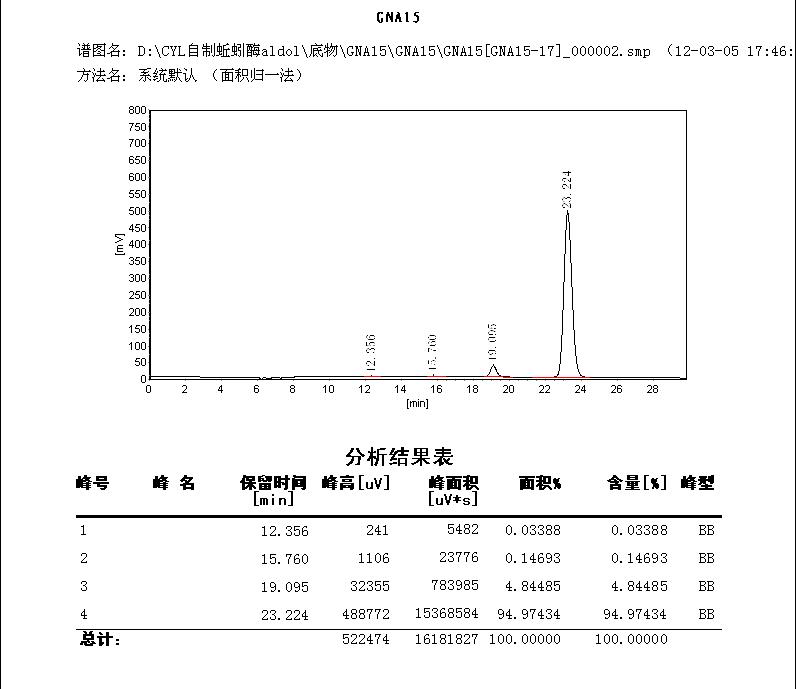
 DEFAULT REPORT

| Peak # | Time [min] | Height [μv] | Area [μv.s] | Area [%] |
| --- | --- | --- | --- | --- |
| 1 | 12.356 | 241 | 5482 | 0.03388 |
| 2 | 15.760 | 1106 | 23776 | 0.14693 |
| 3 | 19.095 | 32355 | 783985 | 4.84485 |
| 4 | 23.224 | 488772 | 15368584 | 94.97434 |
| [Sum](http://www.nciku.cn/search/en/sum) |  | 522474 | 16181827 | 100.00000 |

**2-(Hydroxy-(3-methoxy-phenyl)-methyl)cyclohexan-1-one (****3e):** [[^5^](#_ENREF_6)]


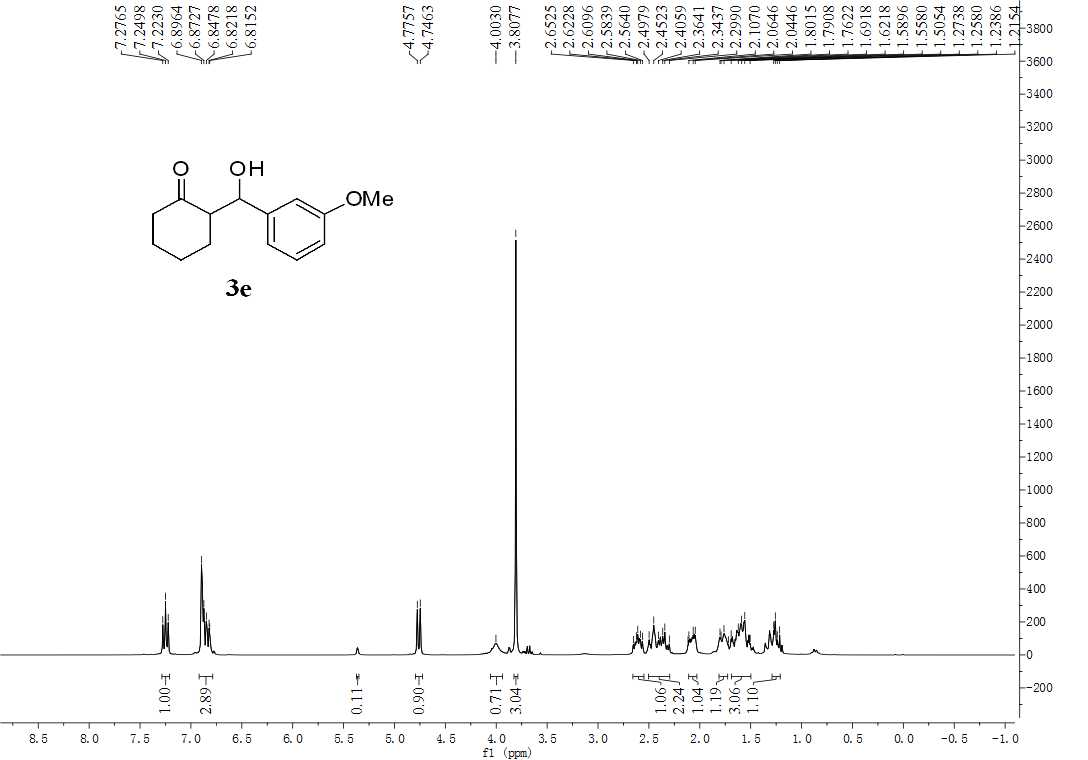


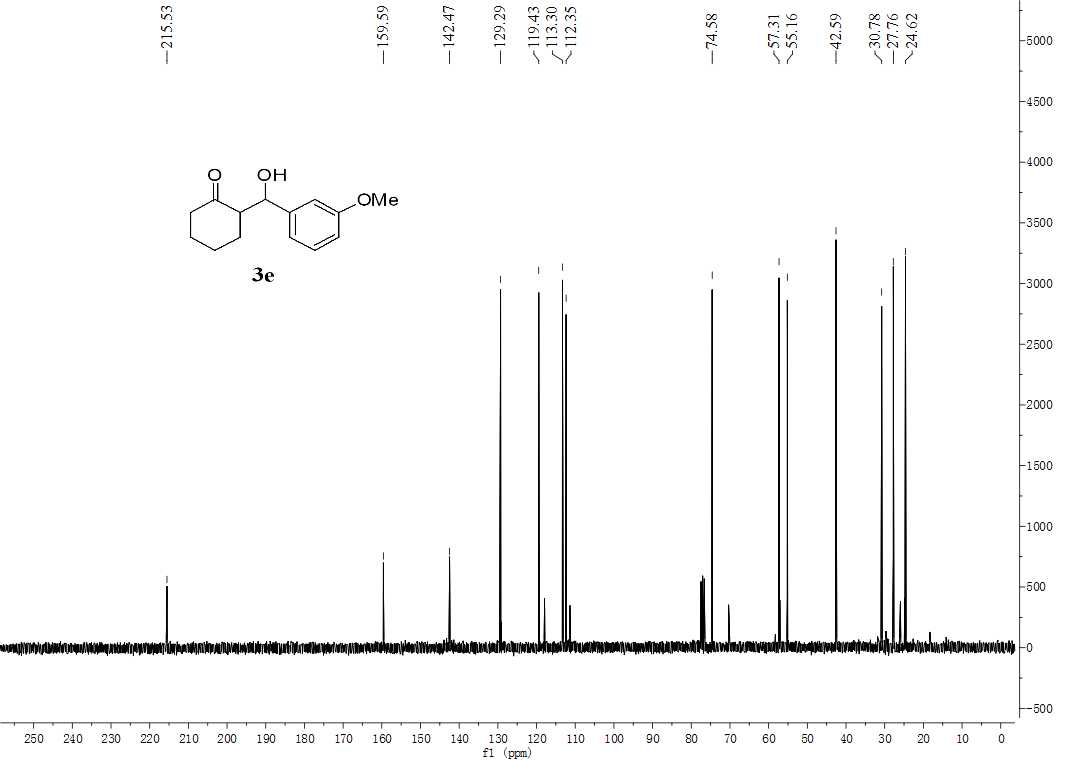


**3e (Racemic)**


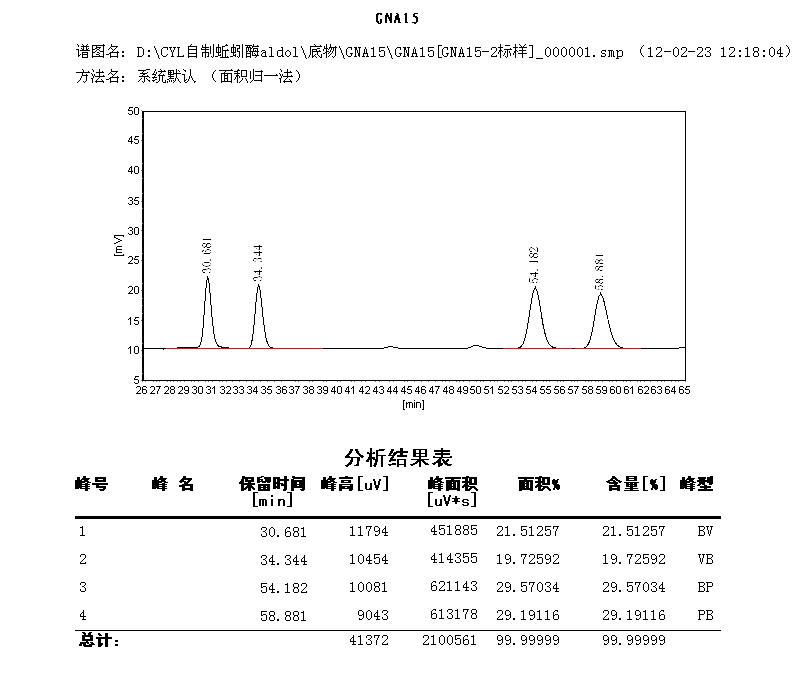


DEFAULT REPORT

| Peak # | Time [min] | Height [μv] | Area [μv.s] | Area [%] |
| --- | --- | --- | --- | --- |
| 1 | 30.681 | 11794 | 451885 | 21.51257 |
| 2 | 34.344 | 10454 | 414355 | 19.72592 |
| 3 | 54.182 | 10081 | 621143 | 29.57034 |
| 4 | 58.881 | 9043 | 613178 | 29.19116 |
| [Sum](http://www.nciku.cn/search/en/sum) |  | 41372 | 2100561 | 99.99999 |

**3e (Chiral)**


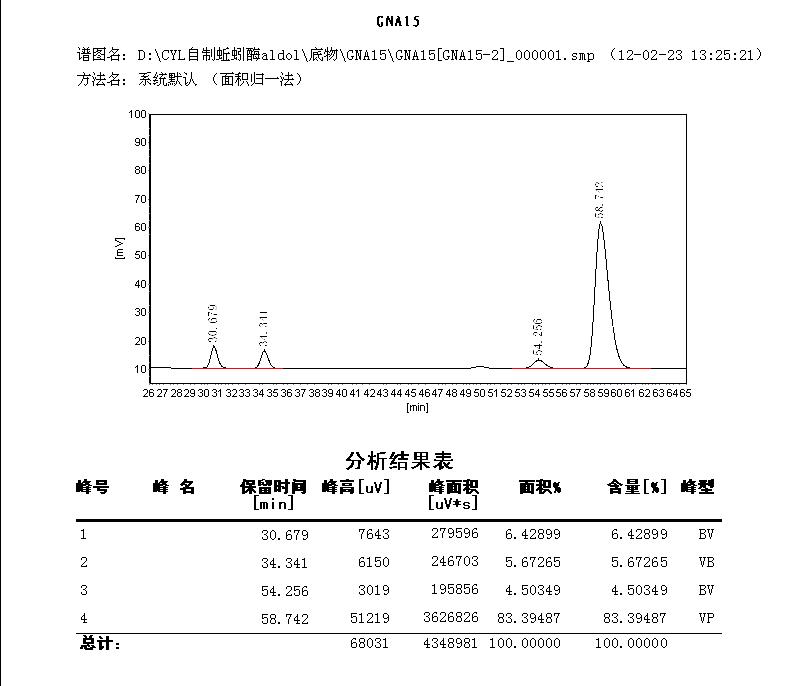


DEFAULT REPORT

| Peak # | Time [min] | Height [μv] | Area [μv.s] | Area [%] |
| --- | --- | --- | --- | --- |
| 1 | 30.679 | 7643 | 279596 | 6.42899 |
| 2 | 34.341 | 6150 | 246703 | 5.67265 |
| 3 | 54.256 | 3019 | 195856 | 4.50349 |
| 4 | 58.742 | 51219 | 3636826 | 83.39487 |
| [Sum](http://www.nciku.cn/search/en/sum) |  | 68031 | 4348981 | 100.00000 |

**3-(Hydroxy(4-nitrophenyl)methyl)tetrahydropyran-4-one (3f) :** [[^6^](#_ENREF_7)]


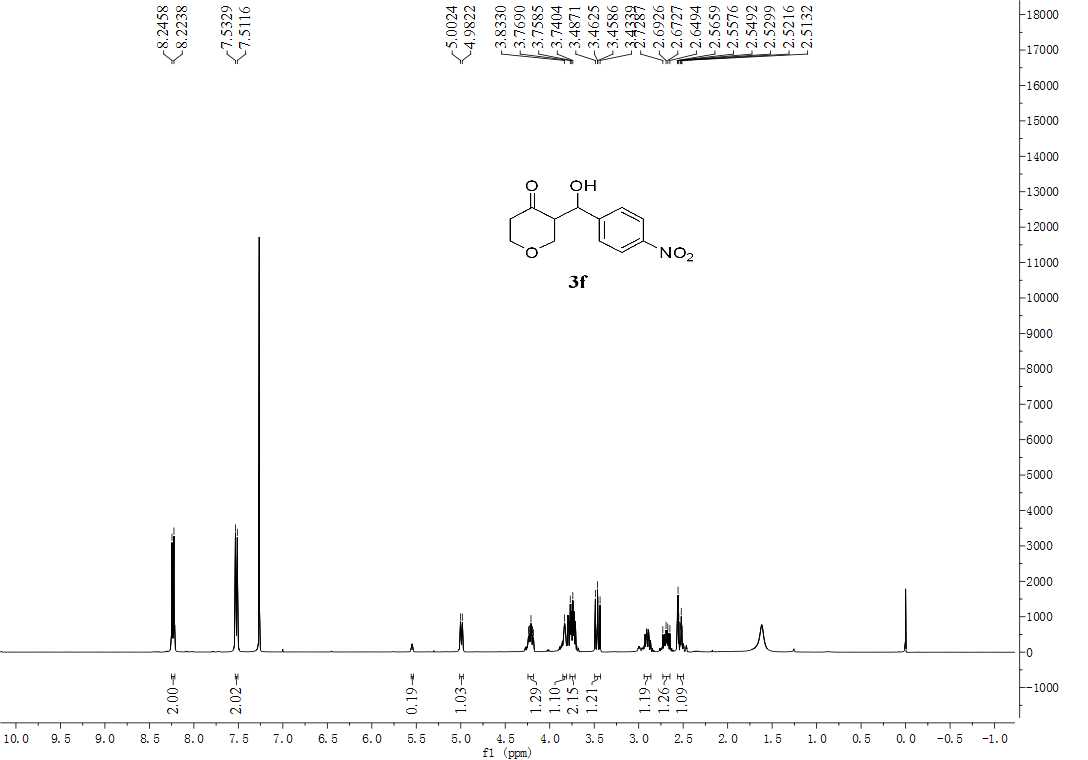


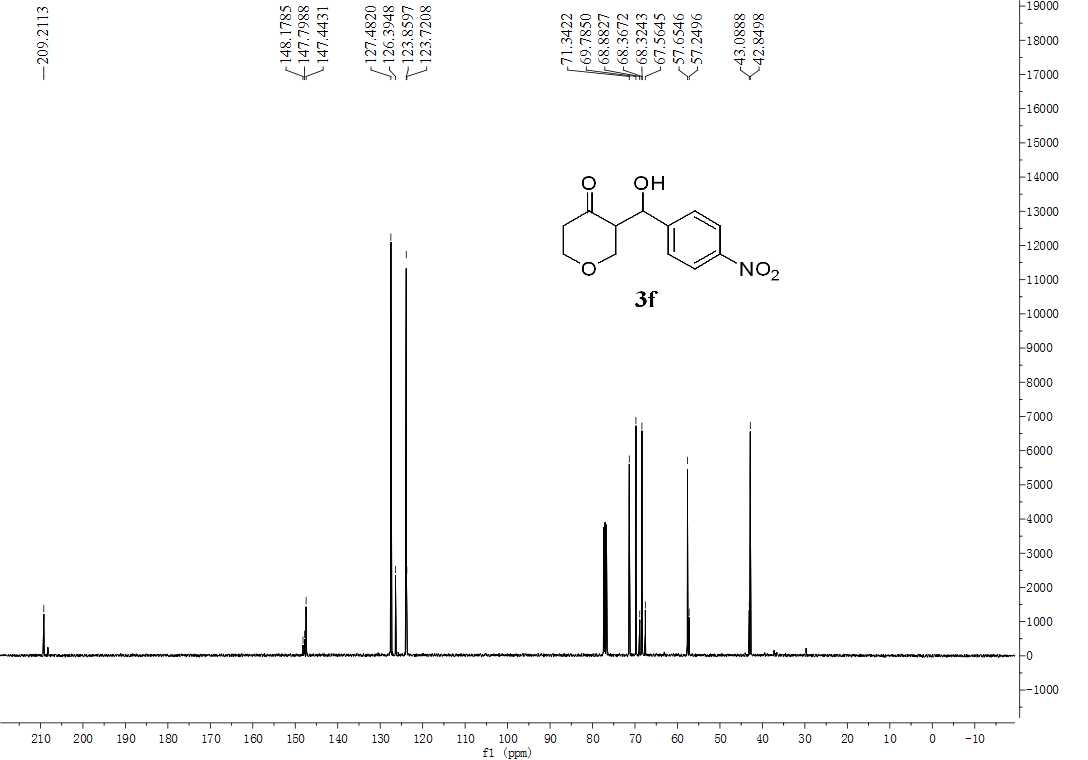


**3f (Racemic)**

**
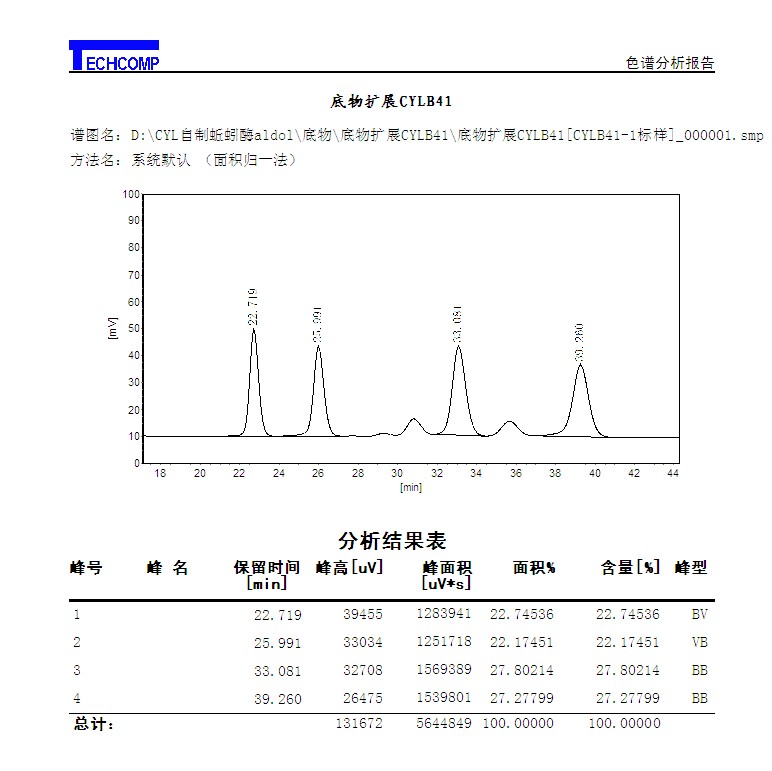
**

DEFAULT REPORT

| Peak # | Time [min] | Height [μv] | Area [μv.s] | Area [%] |
| --- | --- | --- | --- | --- |
| 1 | 22.719 | 39455 | 1283941 | 22.74536 |
| 2 | 25.991 | 33034 | 1251718 | 22.17451 |
| 3 | 33.081 | 32708 | 1569389 | 27.80214 |
| 4 | 39.260 | 26475 | 1539801 | 27.27799 |
| [Sum](http://www.nciku.cn/search/en/sum) |  | 131672 | 5644849 | 100.00000 |

**3f (Chiral)**

**
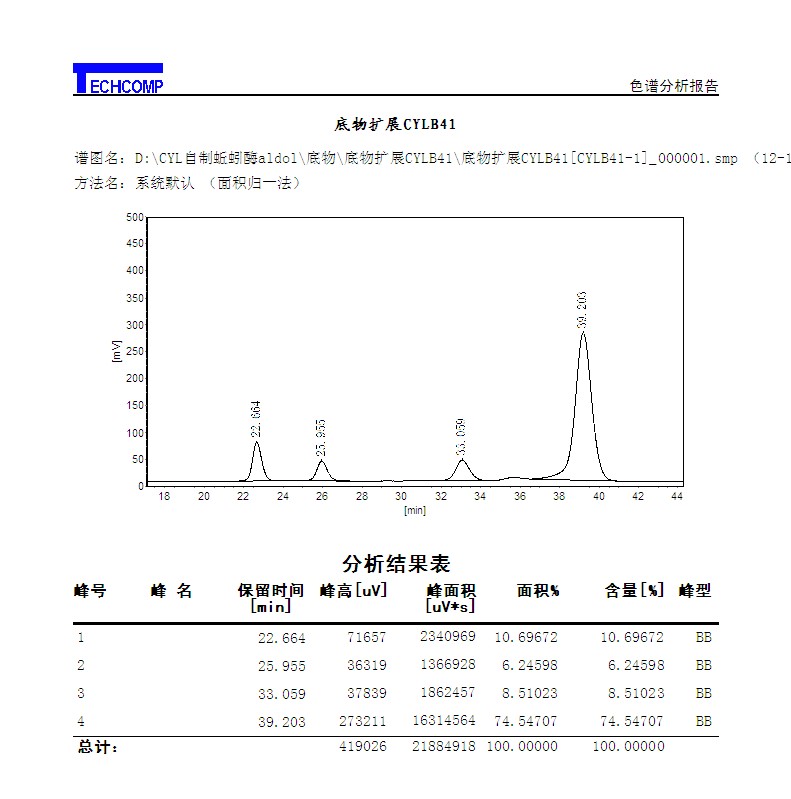
**

DEFAULT REPORT

| Peak # | Time [min] | Height [μv] | Area [μv.s] | Area [%] |
| --- | --- | --- | --- | --- |
| 1 | 22.664 | 71657 | 2340969 | 10.69672 |
| 2 | 25.955 | 36319 | 1866928 | 6.24598 |
| 3 | 33.059 | 37839 | 1862457 | 8.51023 |
| 4 | 39.203 | 273211 | 16314564 | 74.547079 |
| [Sum](http://www.nciku.cn/search/en/sum) |  | 419026 | 21884918 | 100.00000 |

**3-(Hydroxy(4-Cyanophenyl)methyl)-tetrahydrothiopyran-4-one (3g):** [[^6^](#_ENREF_7)]


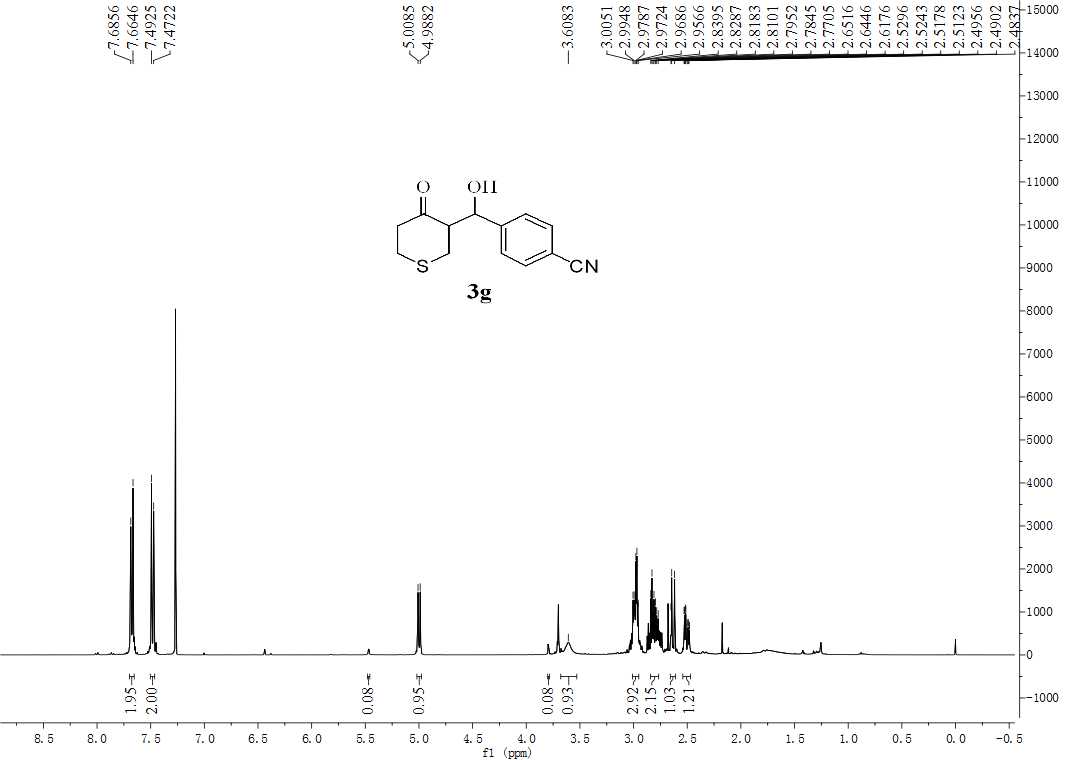


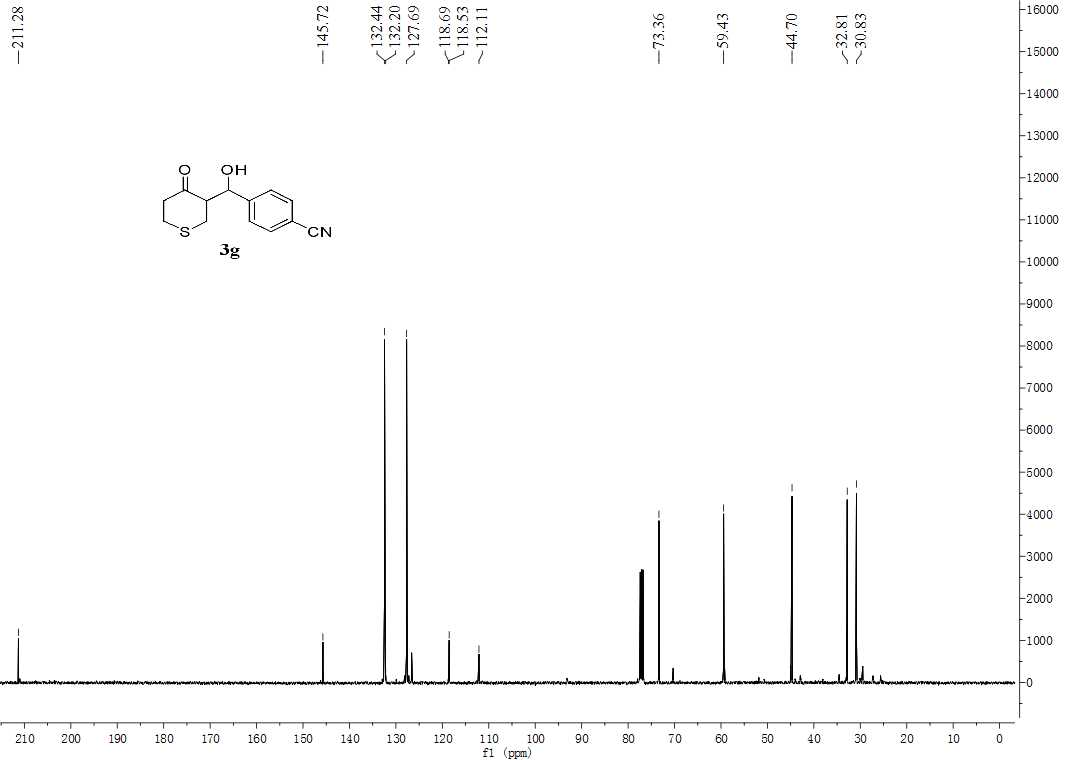


**3g (Racemic)**

**
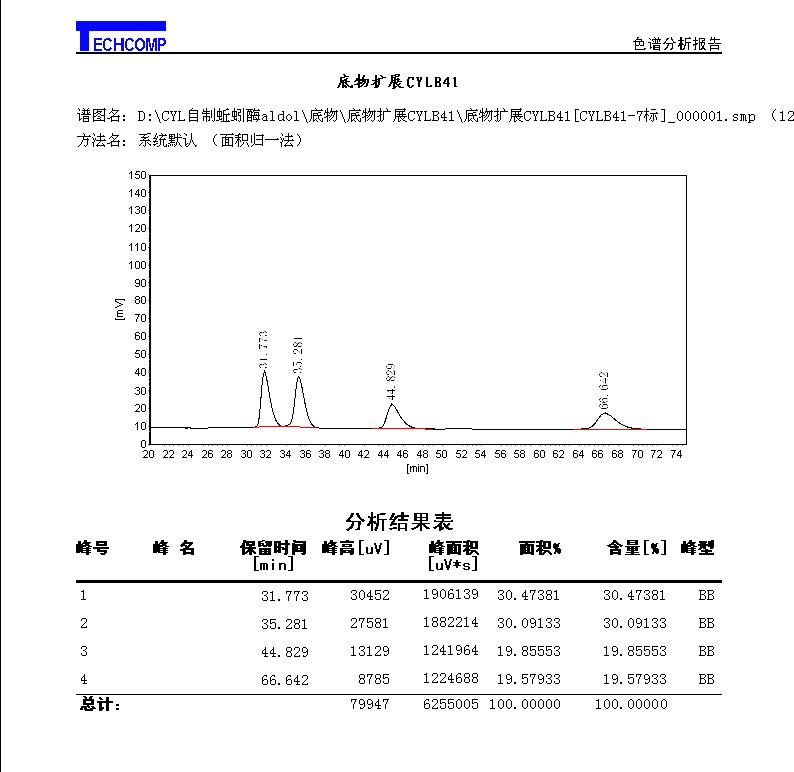
** DEFAULT REPORT

| Peak # | Time [min] | Height [μv] | Area [μv.s] | Area [%] |
| --- | --- | --- | --- | --- |
| 1 | 31.773 | 30452 | 1906139 | 30.47381 |
| 2 | 35.281 | 27581 | 1882214 | 30.09133 |
| 3 | 44.829 | 13129 | 1241964 | 19.85553 |
| 4 | 66.642 | 8785 | 1224688 | 19.57933 |
| [Sum](http://www.nciku.cn/search/en/sum) |  | 79947 | 6255005 | 100.00000 |

**3g (Chiral)**

**
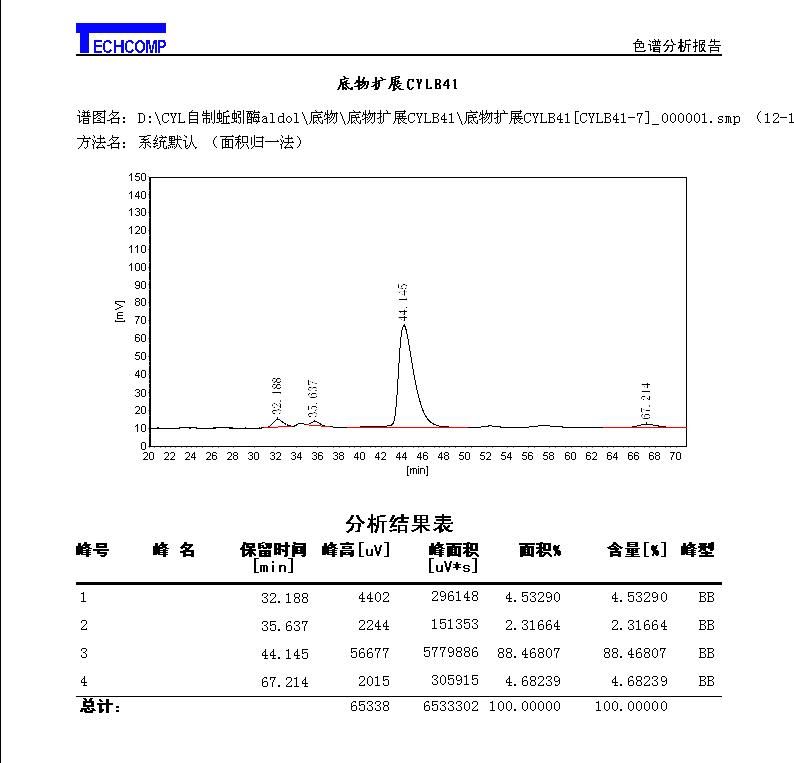
** DEFAULT REPORT

| Peak # | Time [min] | Height [μv] | Area [μv.s] | Area [%] |
| --- | --- | --- | --- | --- |
| 1 | 32.188 | 4402 | 296148 | 4.53290 |
| 2 | 35.637 | 2244 | 151353 | 2.31664 |
| 3 | 44.145 | 56677 | 5779886 | 88.46807 |
| 4 | 67.214 | 2015 | 305915 | 4.68239 |
| [Sum](http://www.nciku.cn/search/en/sum) |  | 65338 | 6533302 | 100.00000 |

**2-(Hydroxy-(4-Cyanophenyl-phenyl)methyl) cyclohexan-1-one (****3h):** [[^5^](#_ENREF_6), [^7^](#_ENREF_8)]


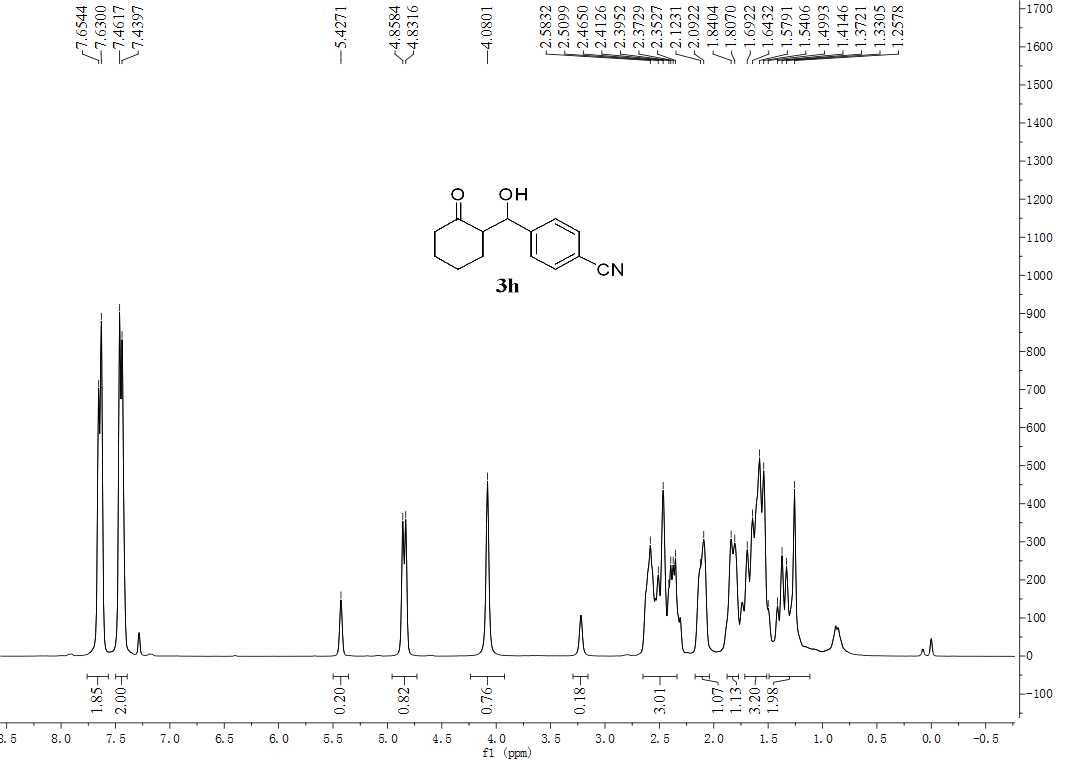


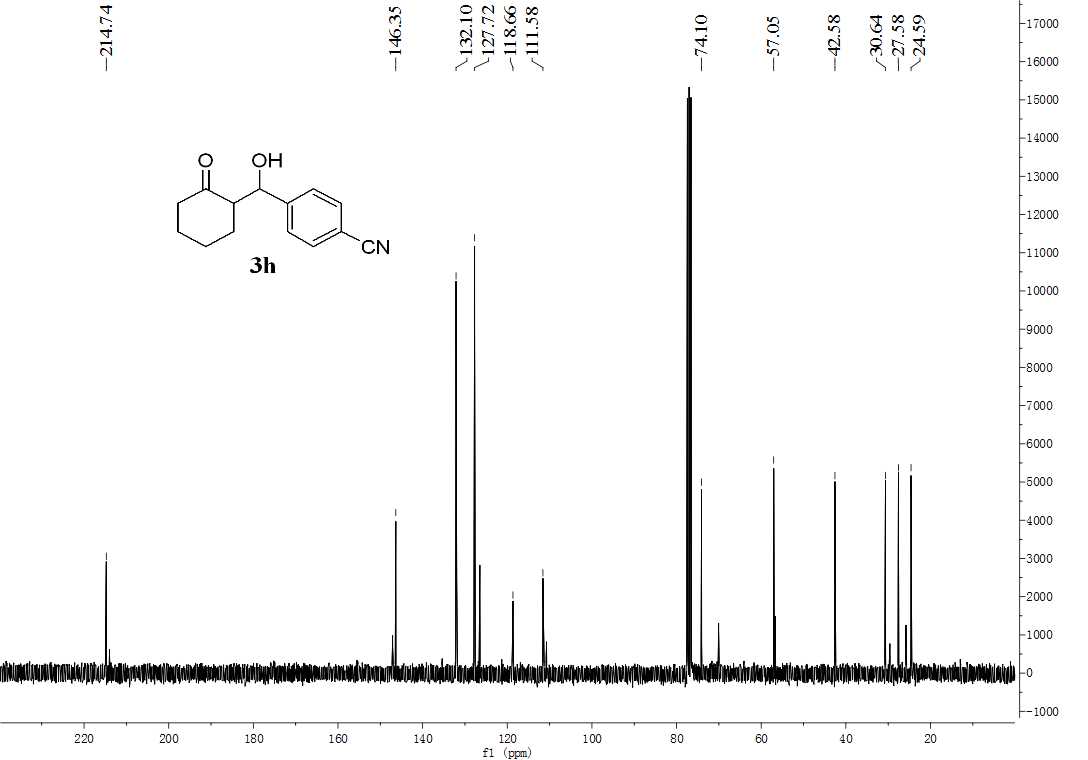


**3h (Racemic)**


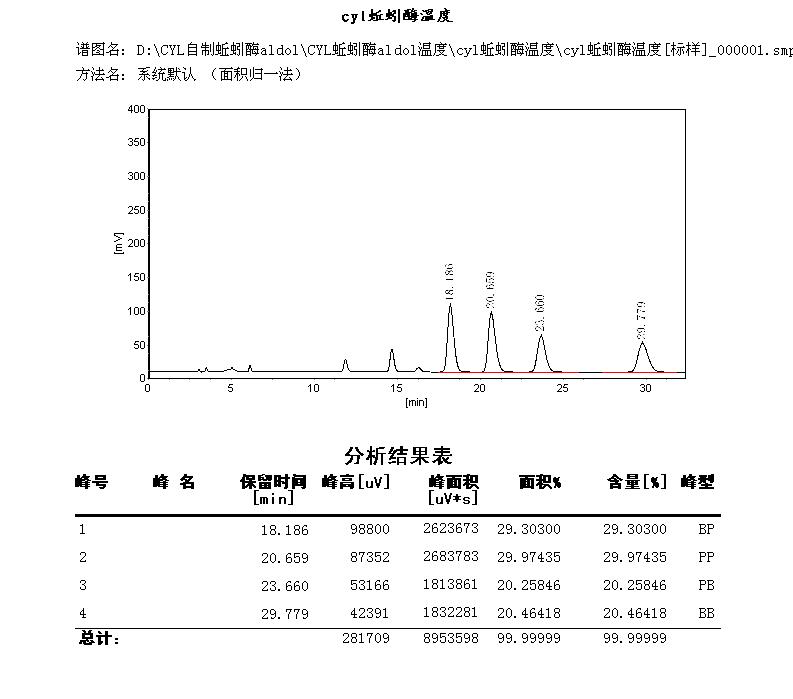
 DEFAULT REPORT

| Peak # | Time [min] | Height [μv] | Area [μv.s] | Area [%] |
| --- | --- | --- | --- | --- |
| 1 | 18.186 | 98800 | 2623673 | 29.30300 |
| 2 | 20.659 | 87352 | 2683783 | 29.97435 |
| 3 | 23.660 | 53166 | 1813861 | 20.25846 |
| 4 | 29.779 | 42391 | 1832281 | 20.46418 |
| [Sum](http://www.nciku.cn/search/en/sum) |  | 281709 | 8953598 | 99.99999 |

**3h (Chiral)**


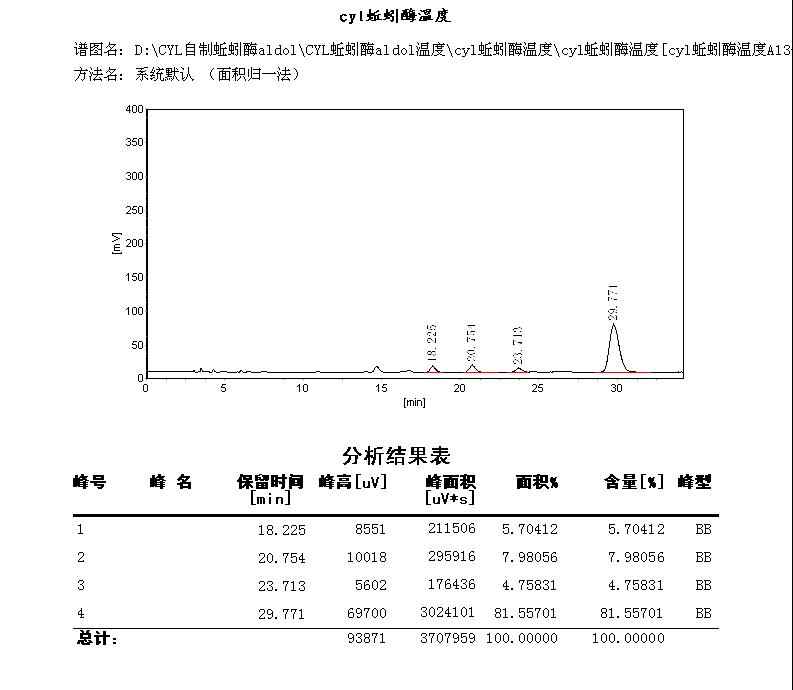
 DEFAULT REPORT

| Peak # | Time [min] | Height [μv] | Area [μv.s] | Area [%] |
| --- | --- | --- | --- | --- |
| 1 | 18.225 | 8551 | 211506 | 5.70412 |
| 2 | 20.754 | 10018 | 295916 | 7.98056 |
| 3 | 23.713 | 5602 | 176436 | 4.75831 |
| 4 | 29.771 | 69700 | 3024101 | 81.55701 |
| [Sum](http://www.nciku.cn/search/en/sum) |  | 93871 | 3707959 | 100.00000 |

**Mannich products**

**2-[(3-bromophenylamino)-(4-nitrophenyl)methyl]cyclohexanone (5a):** ^[8]^


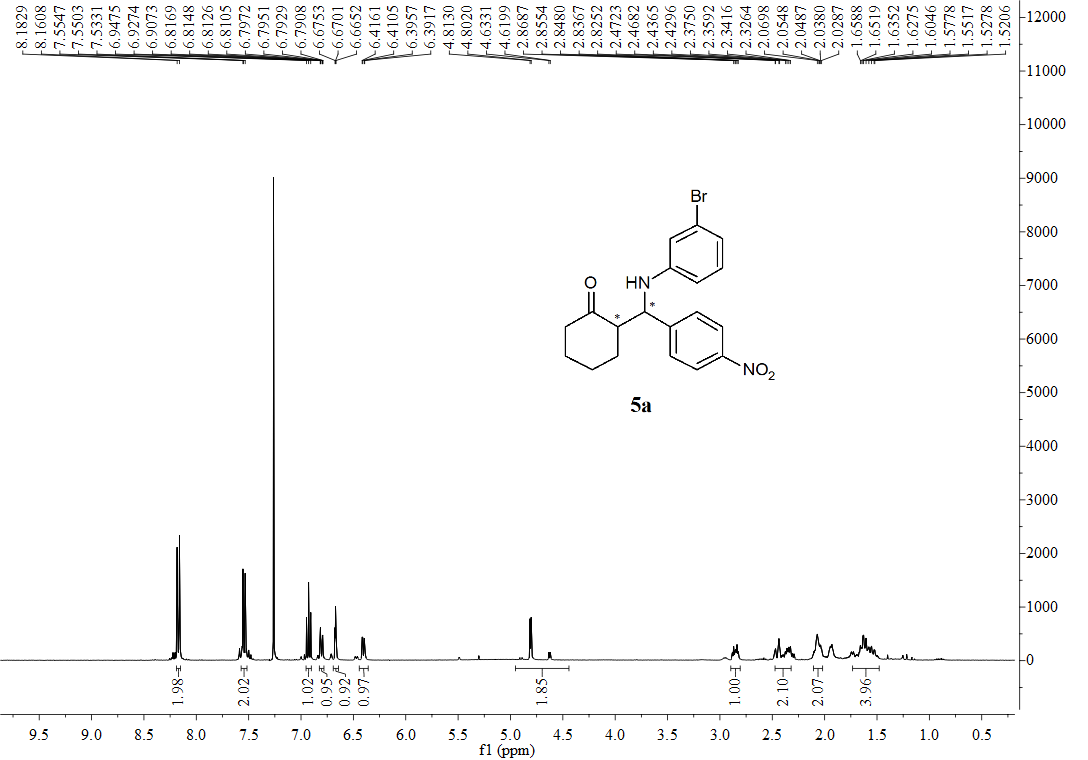


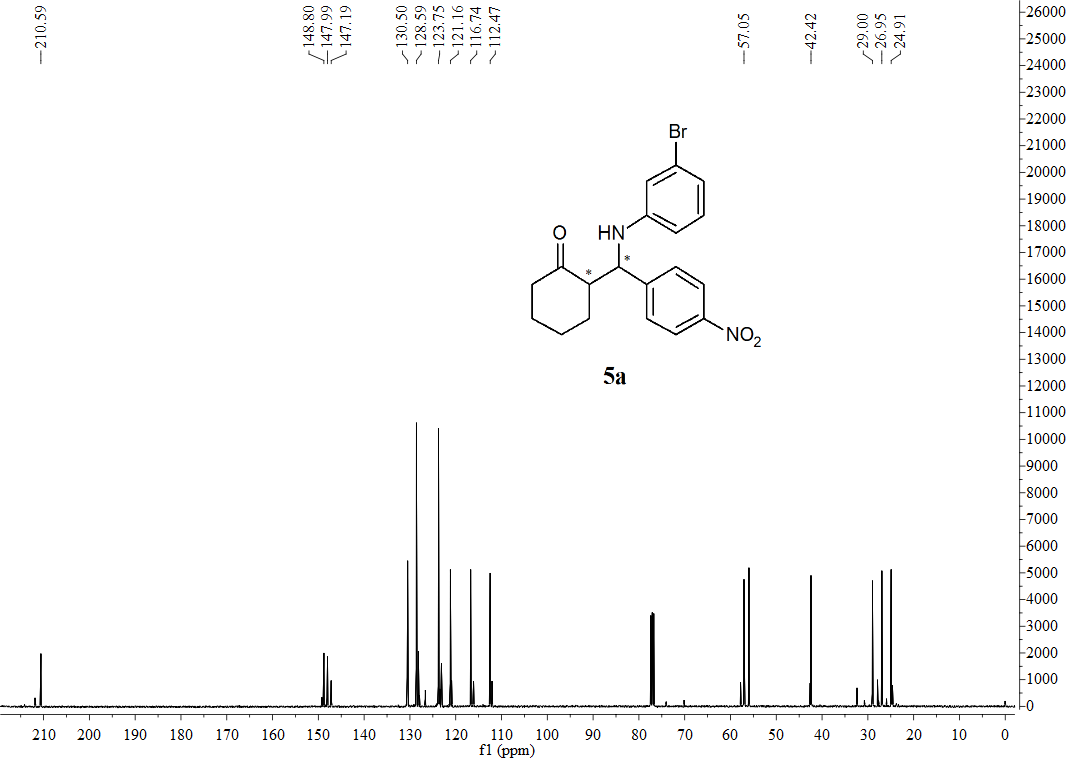


**5a (Racemic)**


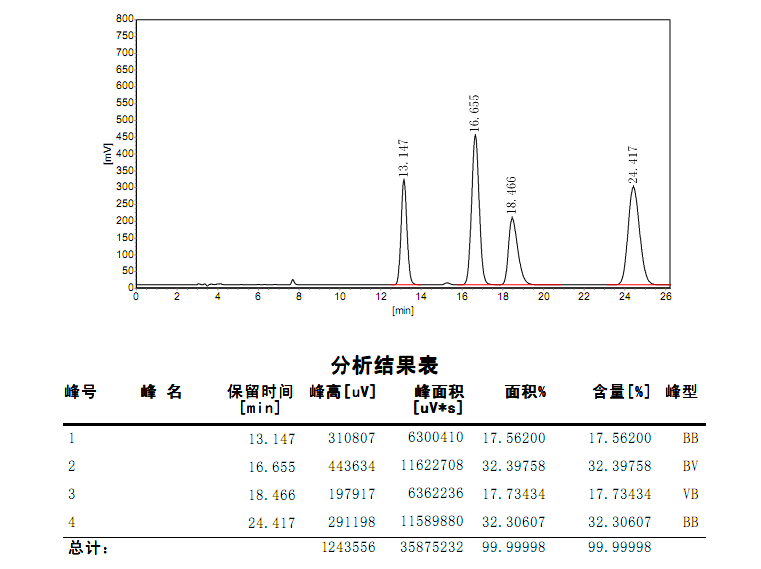


| Peak # | Time [min] | Height [μv] | Area [μv.s] | Area [%] |
| --- | --- | --- | --- | --- |
| 1 | 13.147 | 310807 | 6300410 | 17.56200 |
| 2 | 16.655 | 443634 | 11622708 | 32.39758 |
| 3 | 18.466 | 197917 | 6362236 | 17.73434 |
| 4 | 24.417 | 291198 | 11589880 | 32.30607 |
| [Sum](http://www.nciku.cn/search/en/sum) |  | 1243556 | 35875232 | 99.99998 |

**5a (Chiral)**


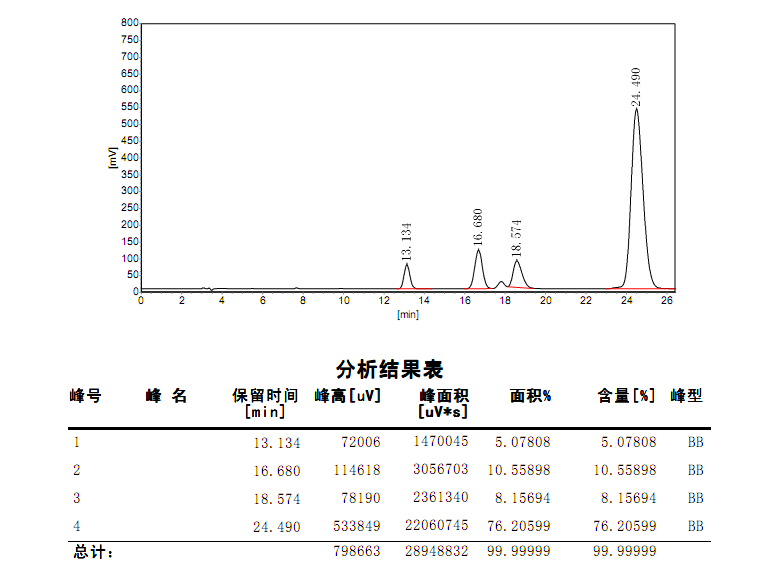


| Peak # | Time [min] | Height [μv] | Area [μv.s] | Area [%] |
| --- | --- | --- | --- | --- |
| 1 | 13.134 | 72006 | 1470045 | 5.07808 |
| 2 | 16.680 | 114618 | 3056703 | 10.55898 |
| 3 | 18.574 | 78190 | 2361340 | 8.15694 |
| 4 | 24.490 | 533849 | 22060745 | 76.20599 |
| [Sum](http://www.nciku.cn/search/en/sum) |  | 798663 | 28948832 | 99.99999 |

**2-[(4-Chlorophenylamino)-(4-nitrophenyl)methyl]cyclohexanone (5b):** ^[8]^


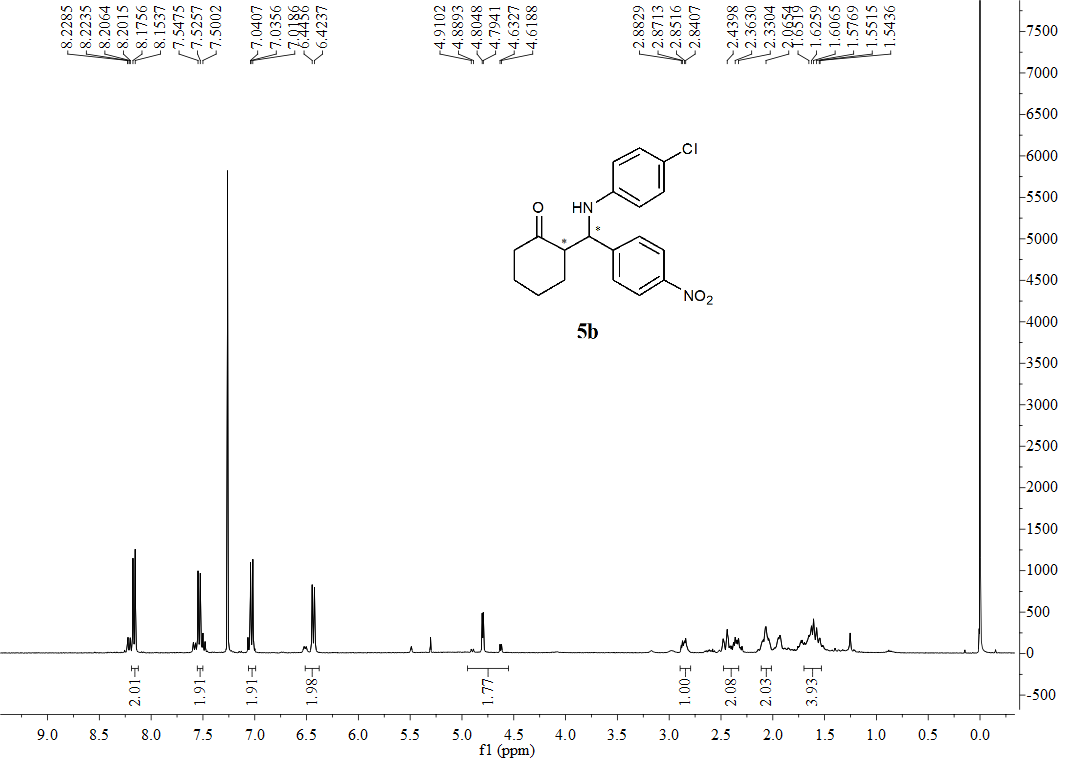


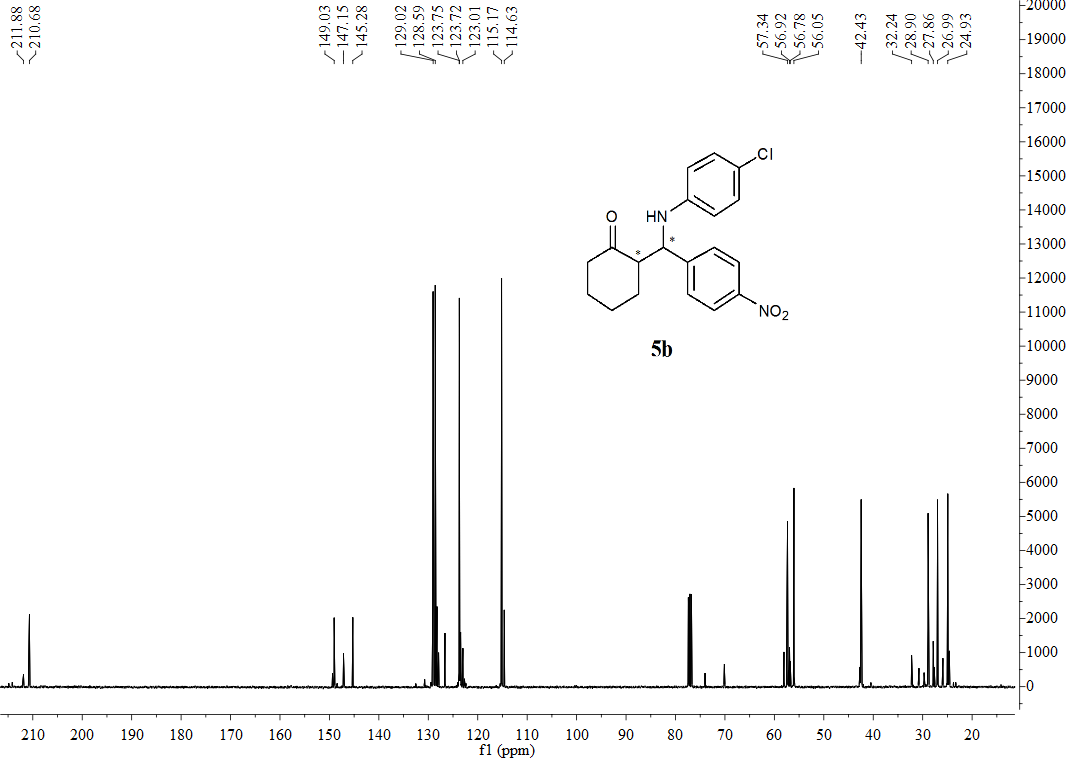


**5b (Racemic)**


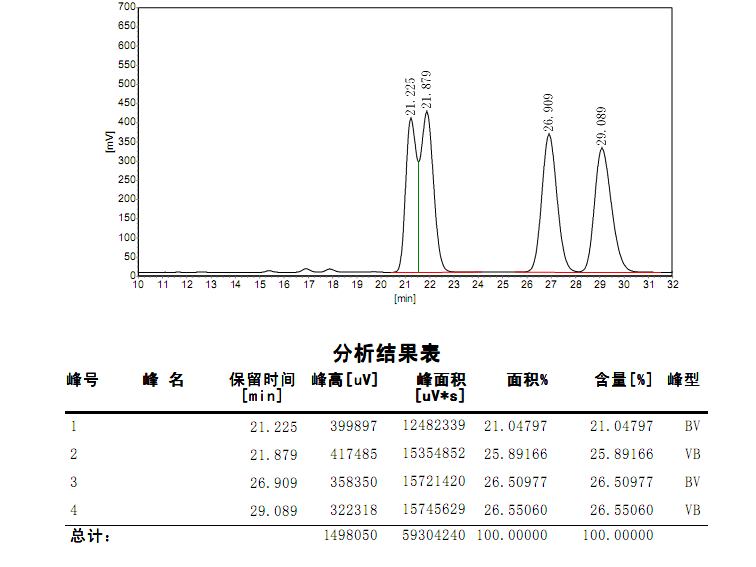


| Peak # | Time [min] | Height [μv] | Area [μv.s] | Area [%] |
| --- | --- | --- | --- | --- |
| 1 | 21.225 | 399897 | 12482339 | 21.04797 |
| 2 | 21.879 | 417485 | 15354852 | 25.89166 |
| 3 | 26.909 | 358350 | 15721420 | 26.50977 |
| 4 | 29.089 | 322318 | 15745629 | 26.55060 |
| [Sum](http://www.nciku.cn/search/en/sum) |  | 1498050 | 59304240 | 100.00000 |

**5b (Chiral)**


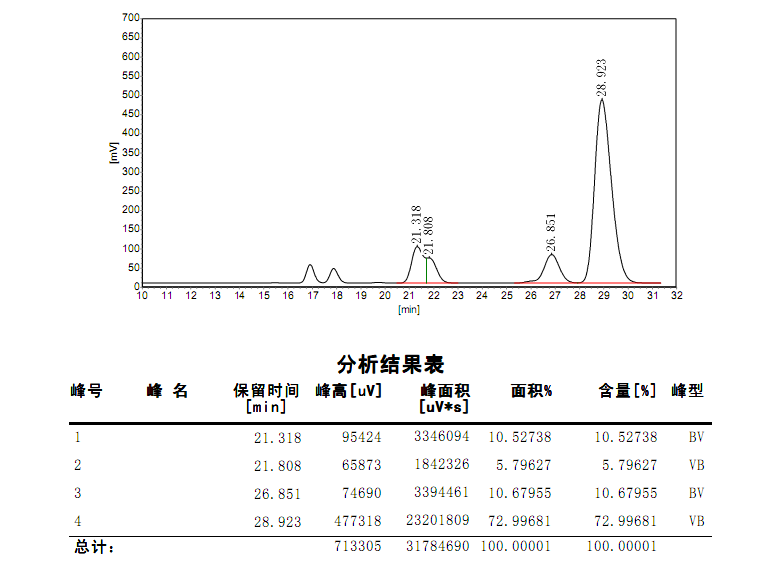


| Peak # | Time [min] | Height [μv] | Area [μv.s] | Area [%] |
| --- | --- | --- | --- | --- |
| 1 | 21.318 | 95424 | 3346094 | 10.52738 |
| 2 | 21.808 | 65873 | 1842326 | 5.79627 |
| 3 | 26.851 | 74690 | 3394461 | 10.67955 |
| 4 | 28.923 | 477318 | 23201809 | 72.99681 |
| [Sum](http://www.nciku.cn/search/en/sum) |  | 713305 | 31784690 | 100.00001 |

**2-[(3-Methylphenylamino)-(4-nitrophenyl)methyl]cyclohexanone** **(5c):** ^[8]^


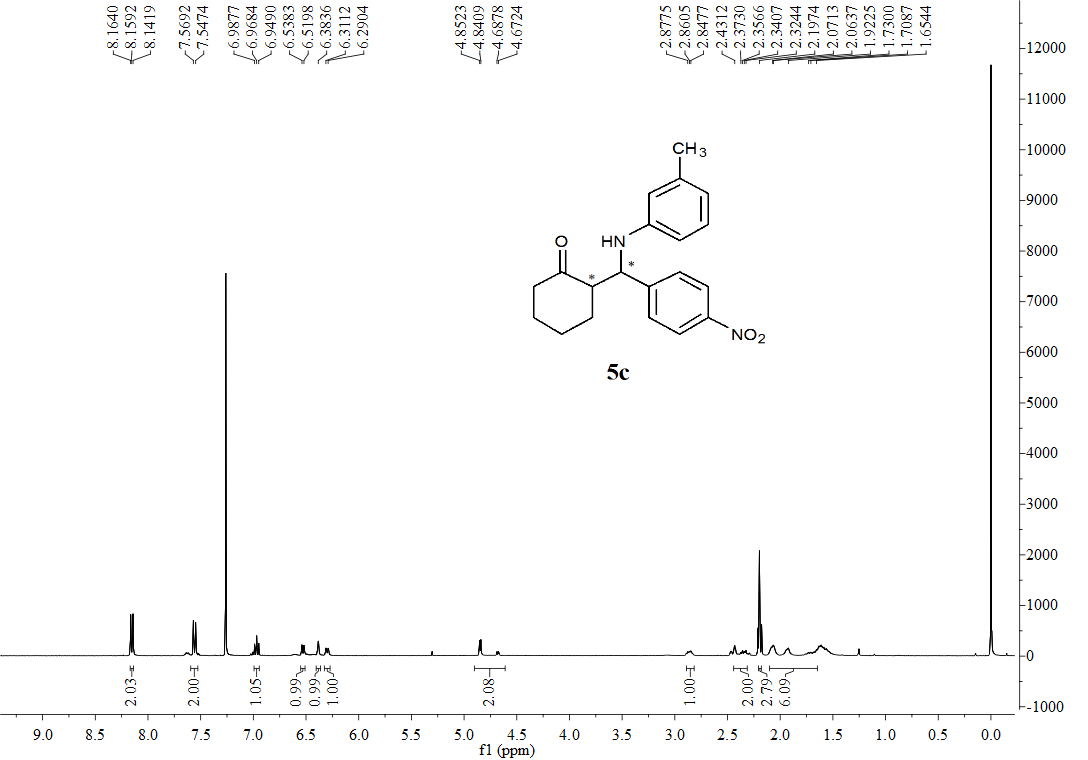


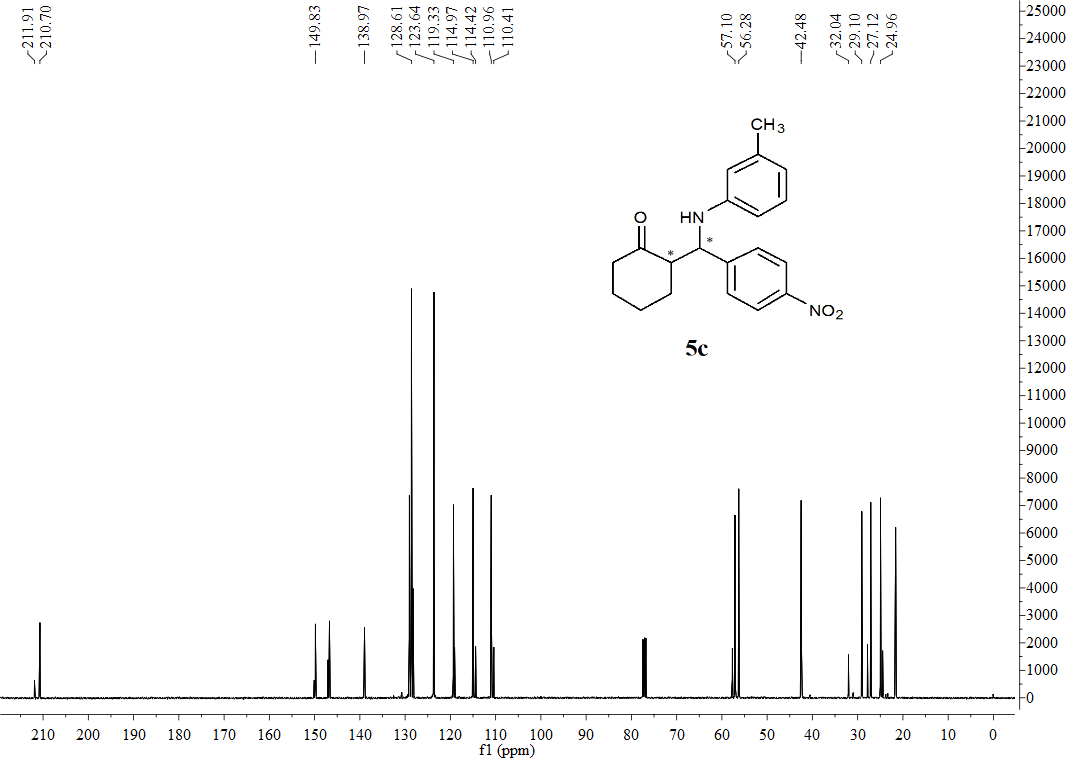


**5c (Racemic)**


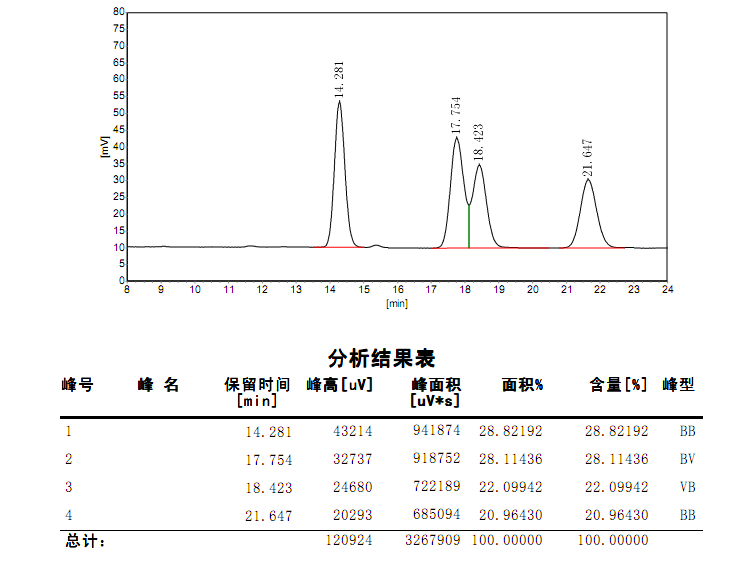


| Peak # | Time [min] | Height [μv] | Area [μv.s] | Area [%] |
| --- | --- | --- | --- | --- |
| 1 | 14.281 | 43214 | 941874 | 28.82192 |
| 2 | 17.754 | 32737 | 918752 | 28.11436 |
| 3 | 18.423 | 24680 | 722189 | 22.09942 |
| 4 | 21.647 | 20293 | 685094 | 20.96430 |
| [Sum](http://www.nciku.cn/search/en/sum) |  | 120924 | 3267909 | 100.00000 |

**5c (Chiral)**


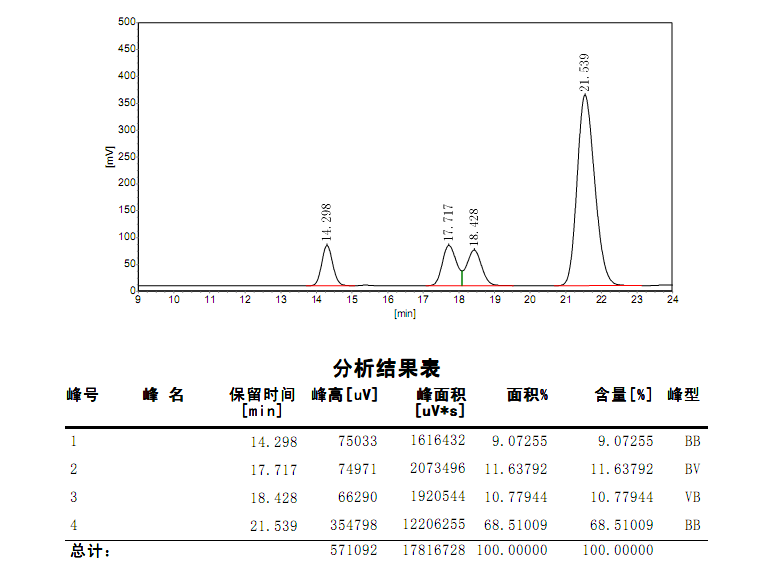


| Peak # | Time [min] | Height [μv] | Area [μv.s] | Area [%] |
| --- | --- | --- | --- | --- |
| 1 | 14.298 | 75033 | 1616432 | 9.07255 |
| 2 | 17.717 | 74971 | 2073496 | 11.63792 |
| 3 | 18.428 | 66290 | 1920544 | 10.77944 |
| 4 | 21.539 | 354798 | 12206255 | 68.51009 |
| [Sum](http://www.nciku.cn/search/en/sum) |  | 571092 | 17816728 | 100.00000 |

**2-[(4-Methylphenylamino)-(4-nitrophenyl)methyl]cyclohexanone (5d):** ^[8]^


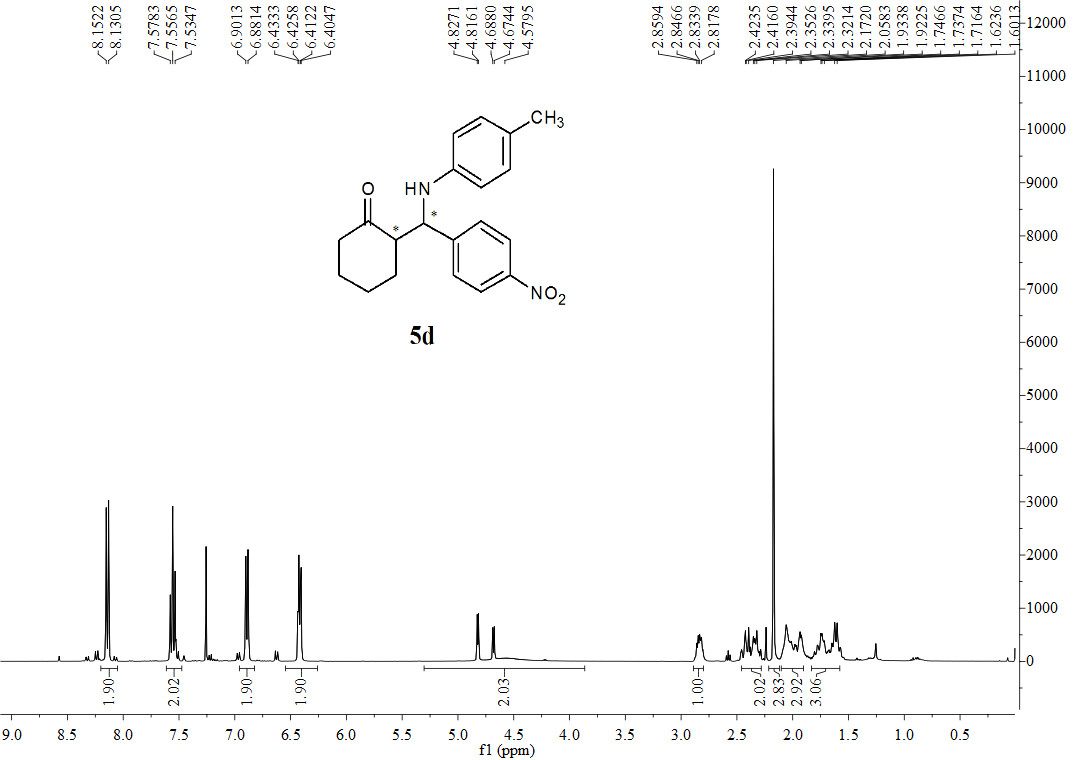


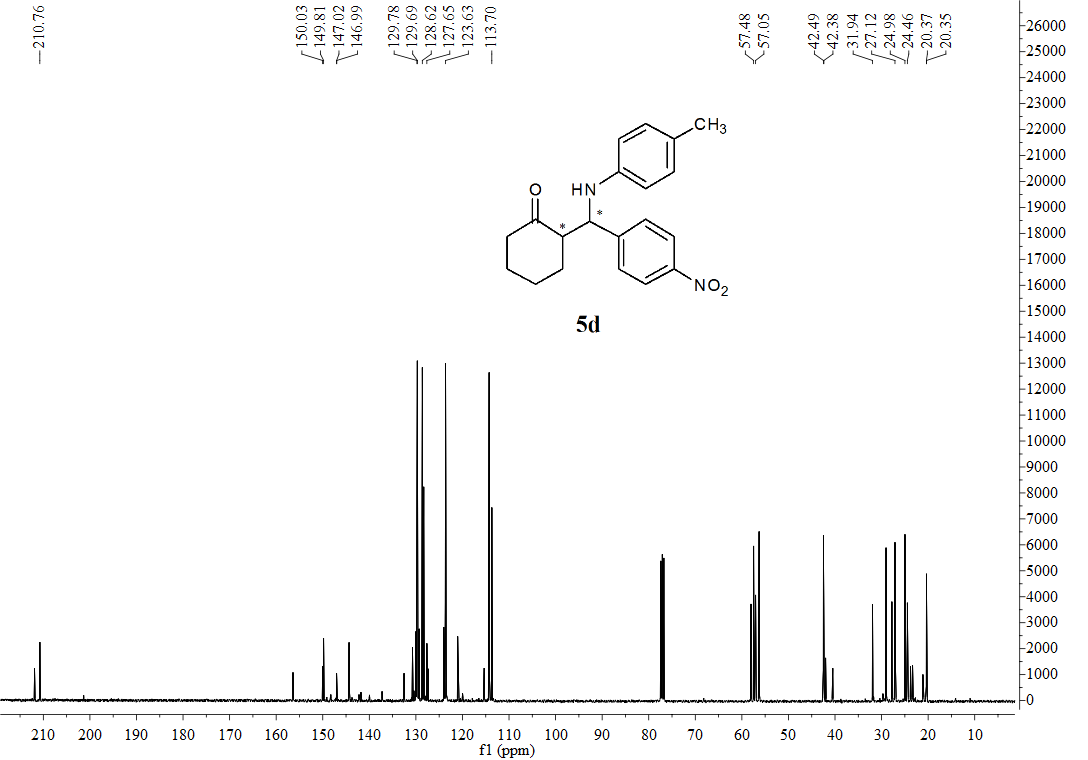


**5d (Racemic)**


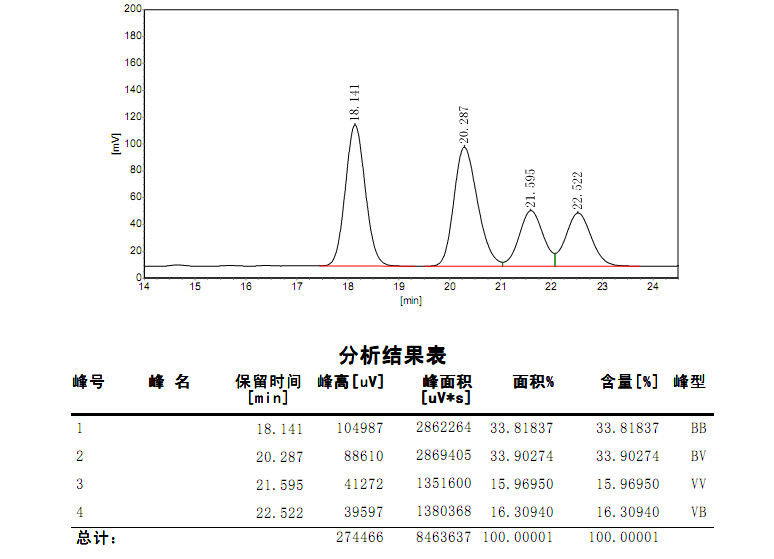


| Peak # | Time [min] | Height [μv] | Area [μv.s] | Area [%] |
| --- | --- | --- | --- | --- |
| 1 | 18.141 | 104987 | 2862264 | 33.81837 |
| 2 | 20.287 | 88610 | 2869405 | 33.90274 |
| 3 | 21.595 | 41272 | 1351600 | 15.96950 |
| 4 | 22.522 | 39597 | 1380368 | 16.30940 |
| [Sum](http://www.nciku.cn/search/en/sum) |  | 274466 | 8463637 | 100.00001 |

**5d (Chiral)**


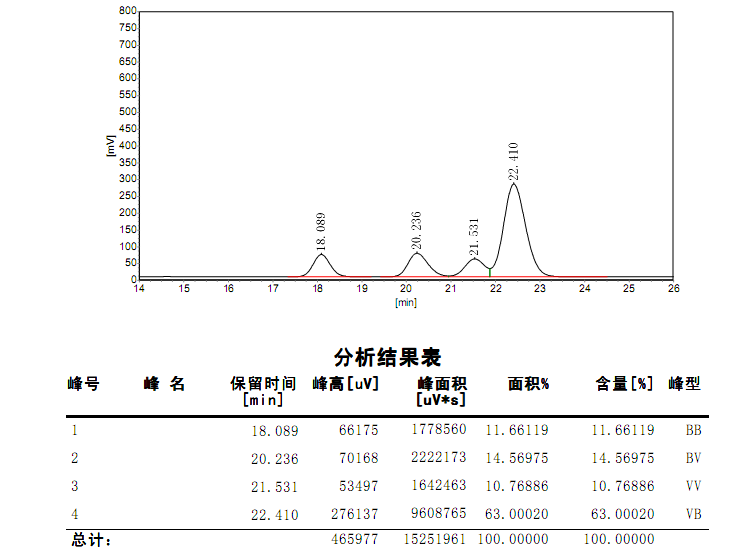


| Peak # | Time [min] | Height [μv] | Area [μv.s] | Area [%] |
| --- | --- | --- | --- | --- |
| 1 | 18.089 | 66175 | 1778560 | 11.66119 |
| 2 | 20.236 | 70168 | 2222173 | 14.56975 |
| 3 | 21.531 | 53497 | 1642463 | 10.76886 |
| 4 | 22.410 | 276137 | 9608765 | 63.00020 |
| [Sum](http://www.nciku.cn/search/en/sum) |  | 465977 | 15251961 | 100.00000 |

**3-[(4-chlorophenyl)(phenylamino)methyl]dihydro-2H-thiopyran-4(3H)-one** **(5e):** ^[9]^


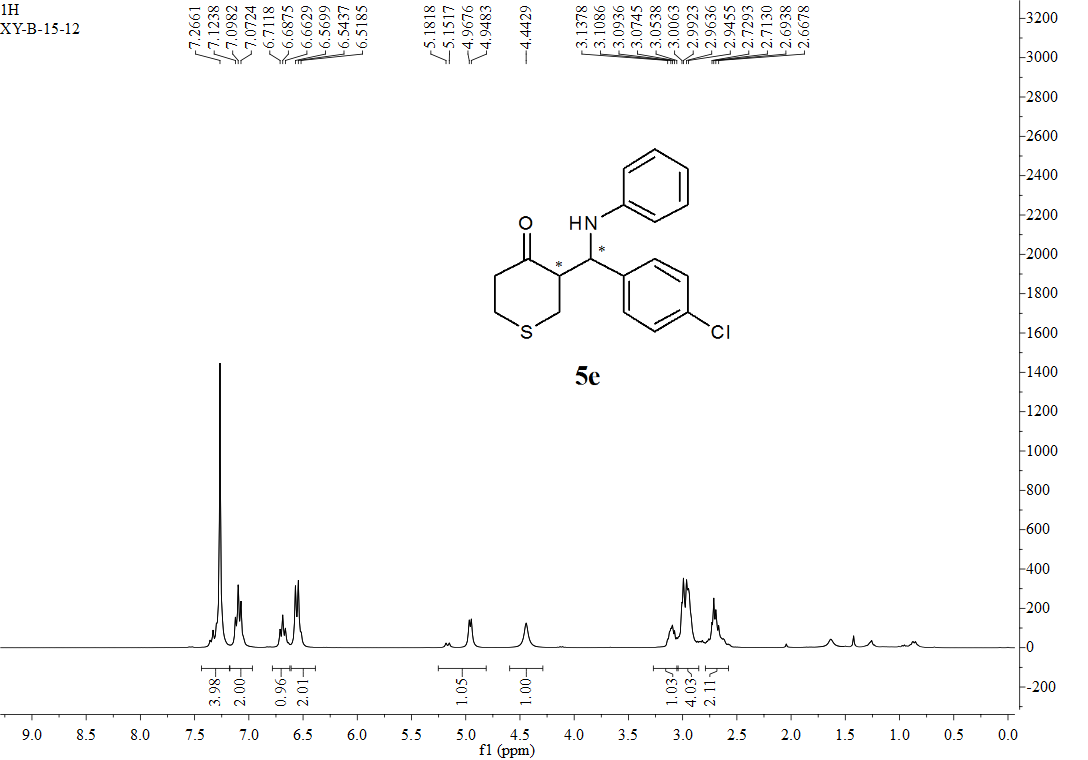


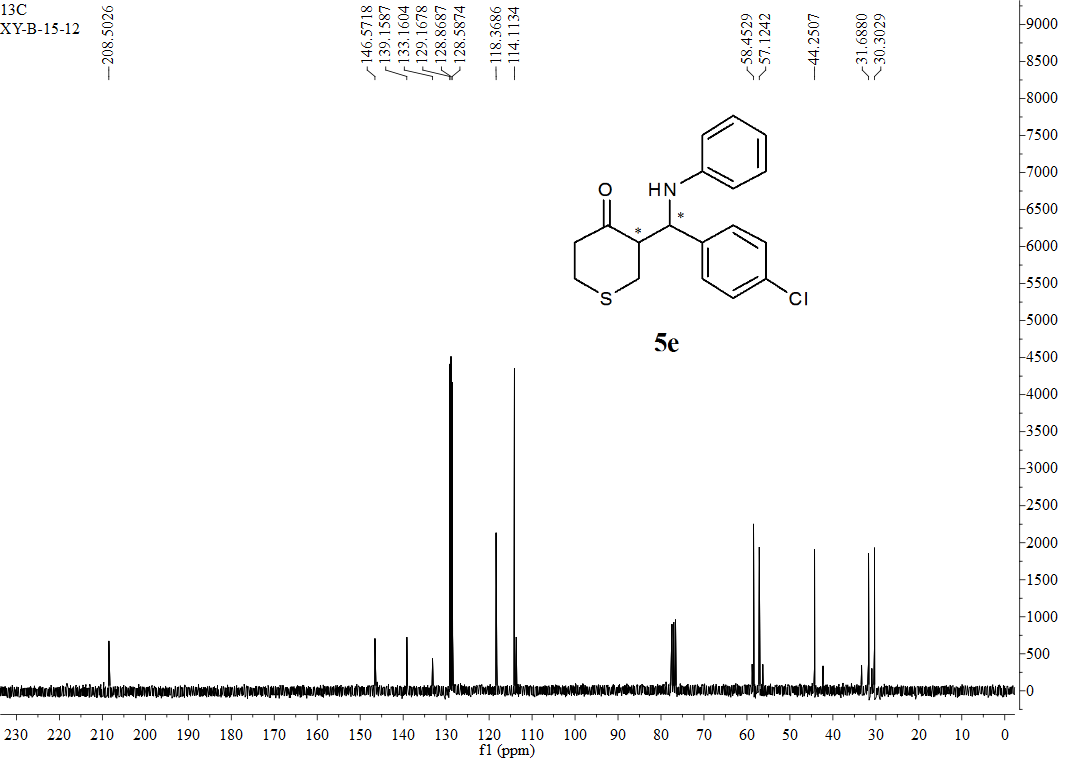


**5e (Racemic)**


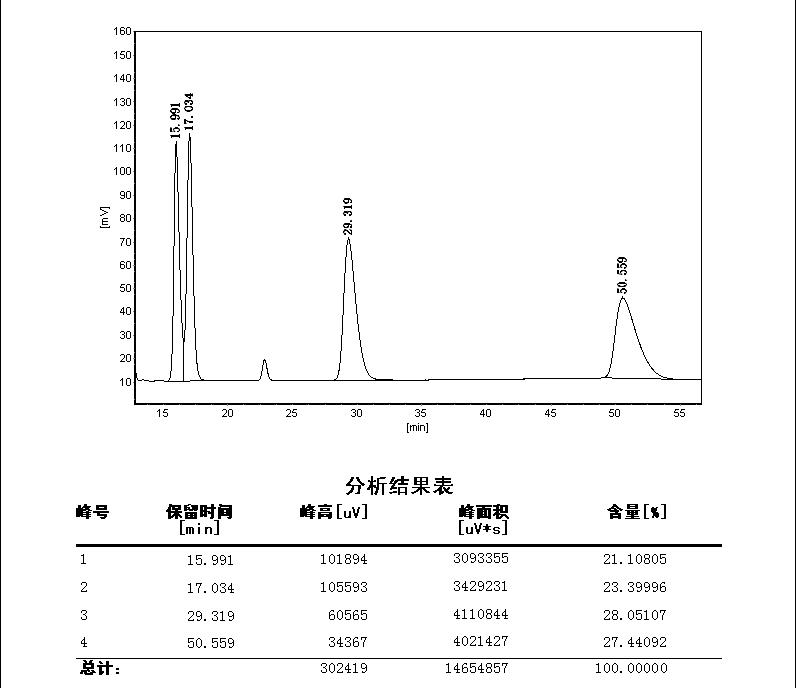


| Peak # | Time [min] | Height [μv] | Area [μv.s] | Area [%] |
| --- | --- | --- | --- | --- |
| 1 | 15.991 | 101894 | 3093355 | 21.10805 |
| 2 | 17.034 | 105593 | 3429231 | 23.39996 |
| 3 | 29.319 | 60565 | 4110844 | 28.05107 |
| 4 | 50.559 | 34367 | 4021427 | 27.44092 |
| [Sum](http://www.nciku.cn/search/en/sum) |  | 302419 | 14654857 | 100.00000 |

**5e (Chiral)**


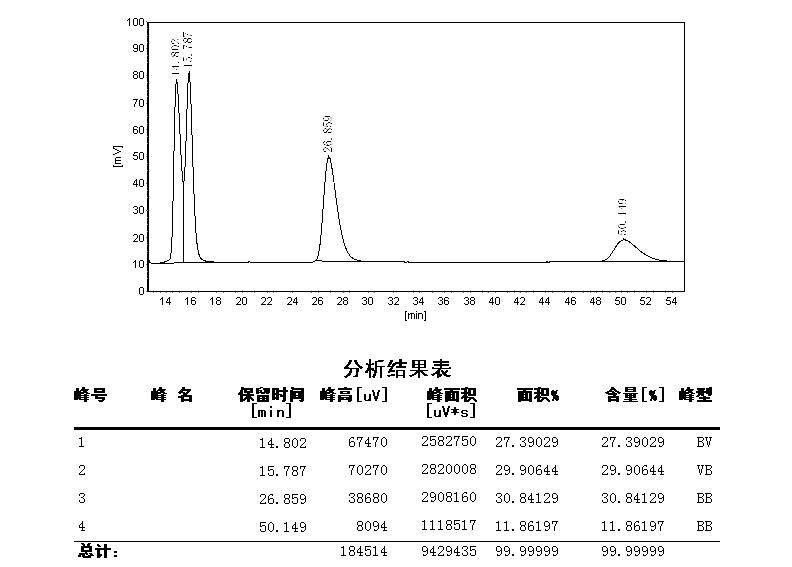


| Peak # | Time [min] | Height [μv] | Area [μv.s] | Area [%] |
| --- | --- | --- | --- | --- |
| 1 | 14.802 | 67470 | 2582750 | 27.39029 |
| 2 | 15.787 | 70270 | 2820008 | 29.90644 |
| 3 | 26.859 | 38680 | 2908160 | 30.84129 |
| 4 | 50.149 | 8094 | 1118517 | 11.86197 |
| [Sum](http://www.nciku.cn/search/en/sum) |  | 184514 | 9429435 | 99.99999 |

**2-[(4-nitrophenyl)(phenylamino)methyl]yclohexanone** **(5f):** ^[9]^


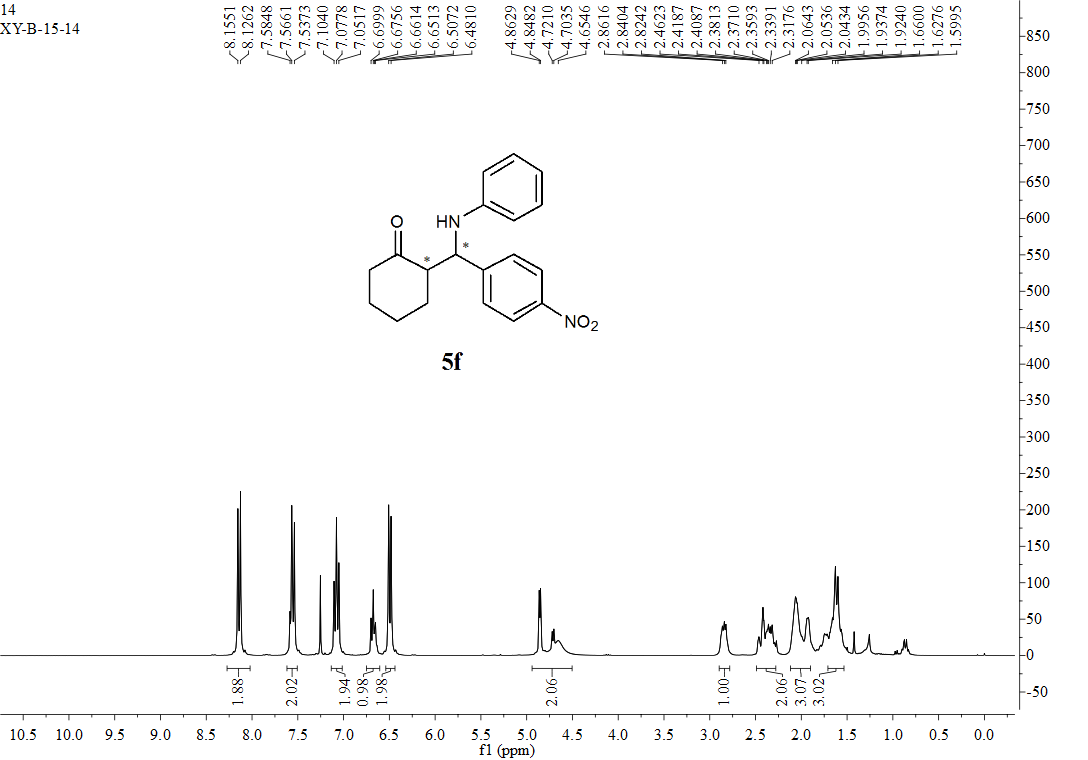


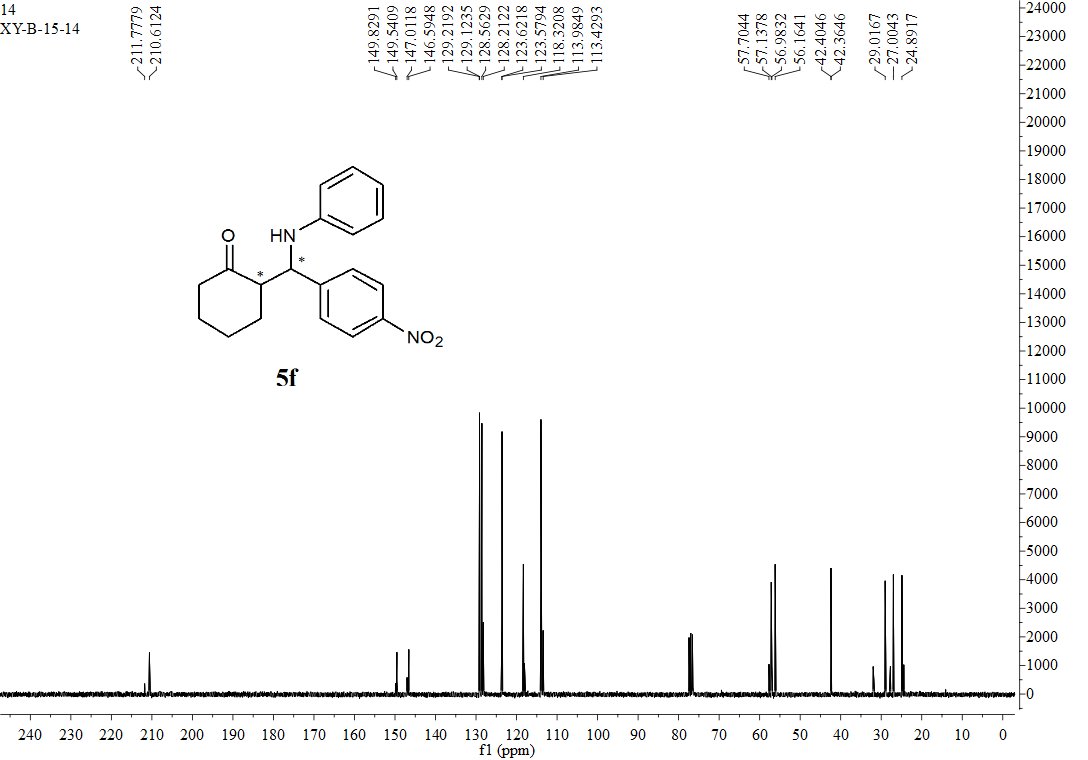


**5f (Racemic)**


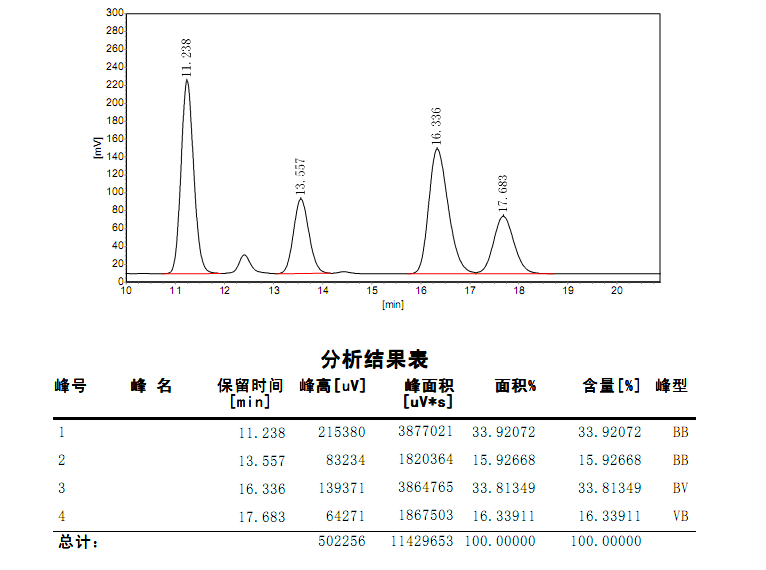


DEFAULT REPORT

| Peak # | Time [min] | Height [μv] | Area [μv.s] | Area [%] |
| --- | --- | --- | --- | --- |
| 1 | 11.238 | 215380 | 3877021 | 33.92072 |
| 2 | 13.557 | 83234 | 1820364 | 15.92668 |
| 3 | 16.336 | 139371 | 3864765 | 33.81349 |
| 4 | 17.683 | 64271 | 1867503 | 16.33911 |
| [Sum](http://www.nciku.cn/search/en/sum) |  | 502256 | 11429653 | 100.00000 |

**5f (Chiral)**


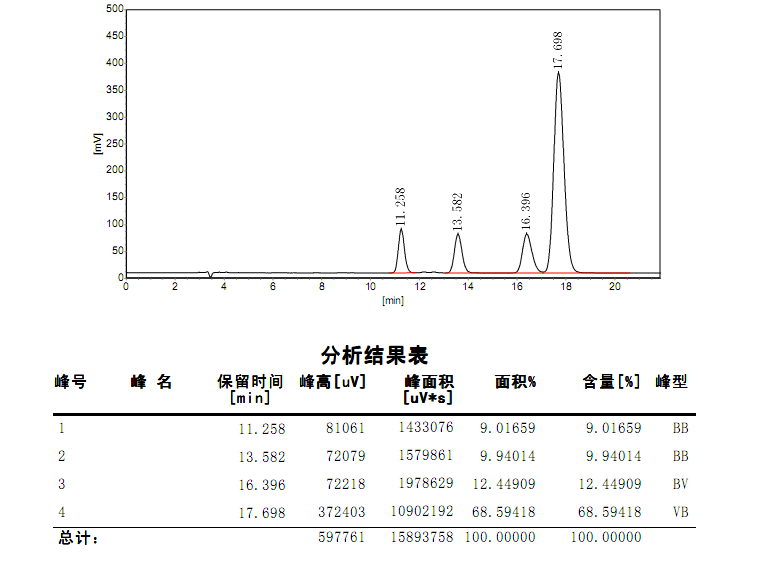


DEFAULT REPORT

| Peak # | Time [min] | Height [μv] | Area [μv.s] | Area [%] |
| --- | --- | --- | --- | --- |
| 1 | 11.258 | 81061 | 1433076 | 9.01659 |
| 2 | 13.582 | 72079 | 1579861 | 9.94014 |
| 3 | 16.396 | 72218 | 1978629 | 12.44909 |
| 4 | 17.698 | 372403 | 10902192 | 68.59418 |
| [Sum](http://www.nciku.cn/search/en/sum) |  | 597761 | 15893758 | 100.00000 |

**Henry products**

**3-(1-hydroxy-2-nitroethyl)benzonitrile (7a):** [[^10]^](#_ENREF_1)


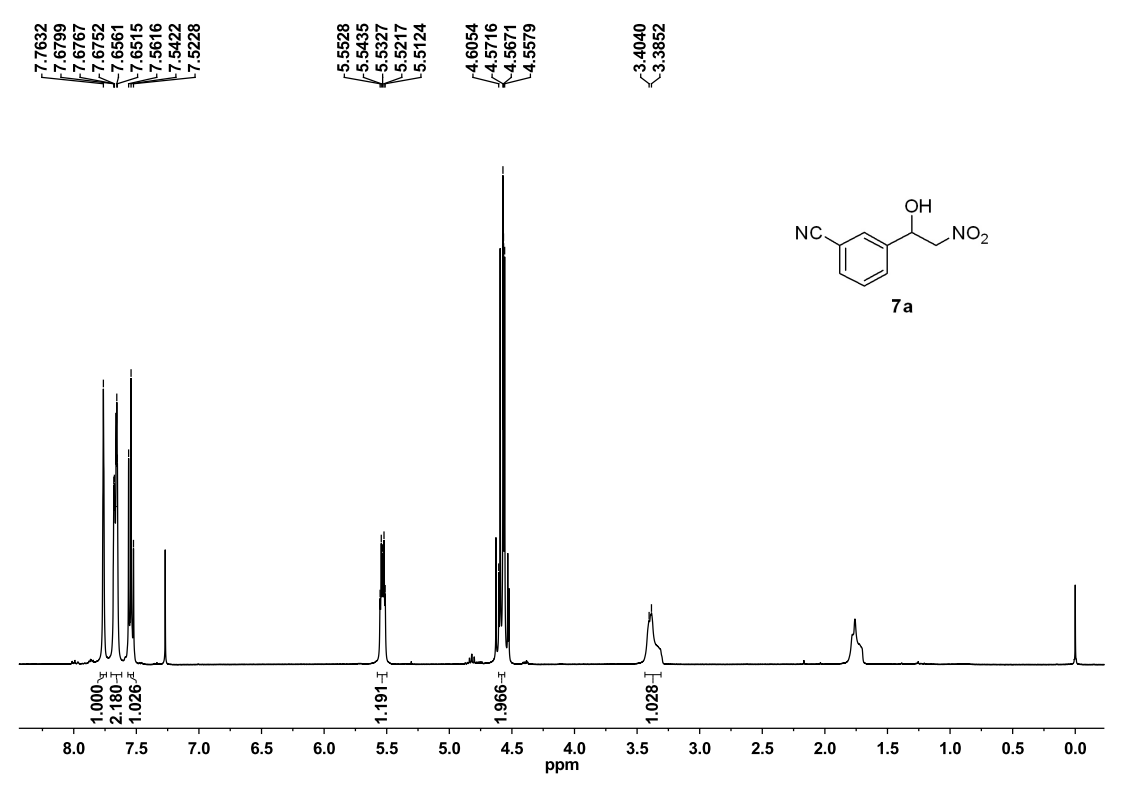


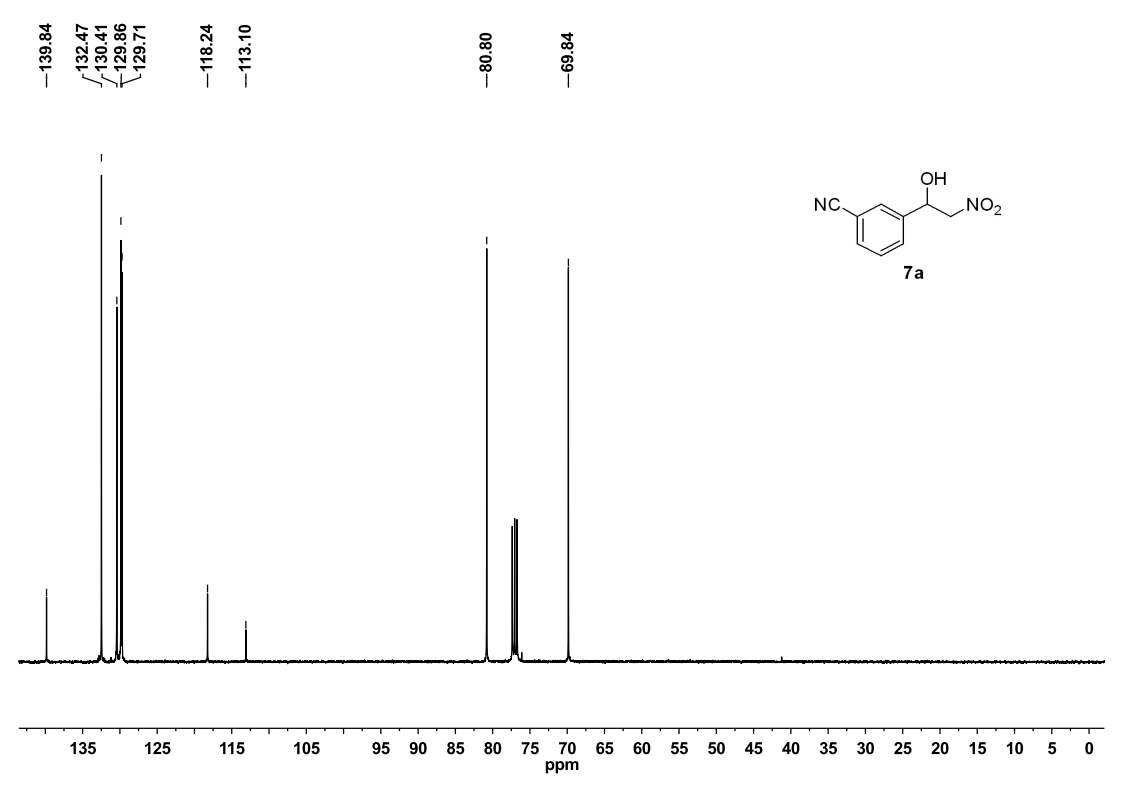


**2-nitro-1-(4-nitrophenyl)ethanol (7b):** [[^10]^](#_ENREF_1)


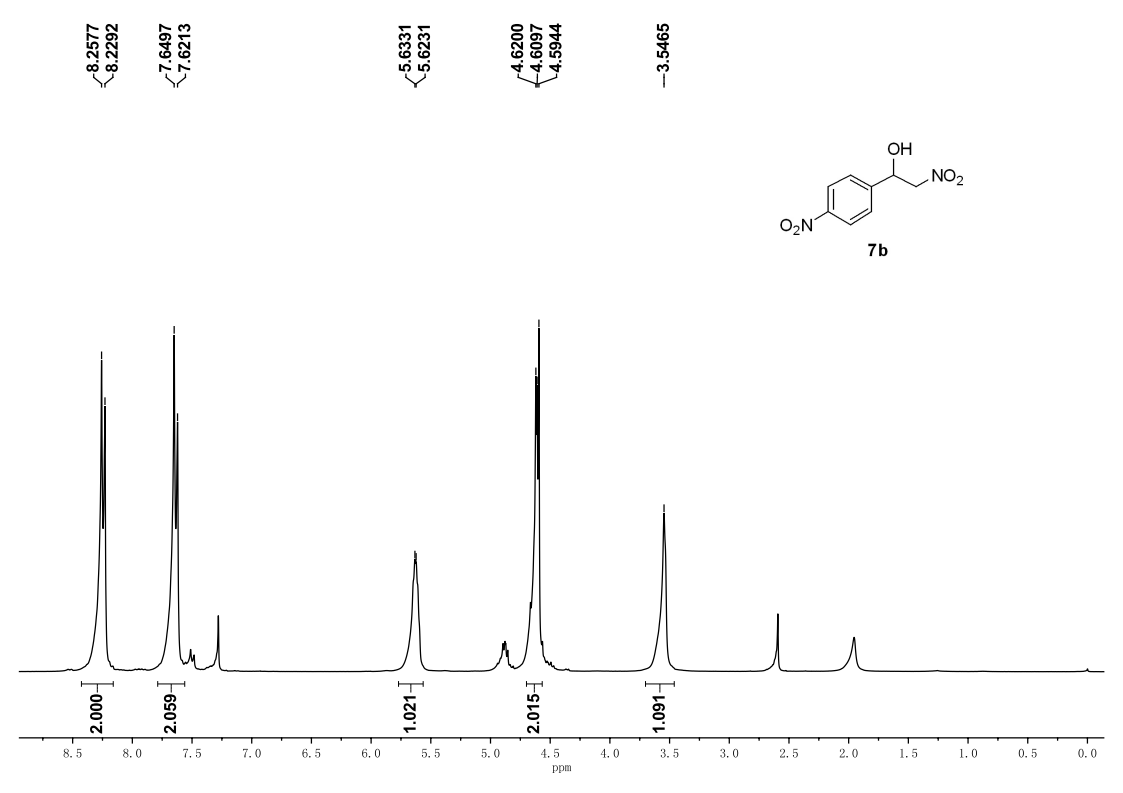


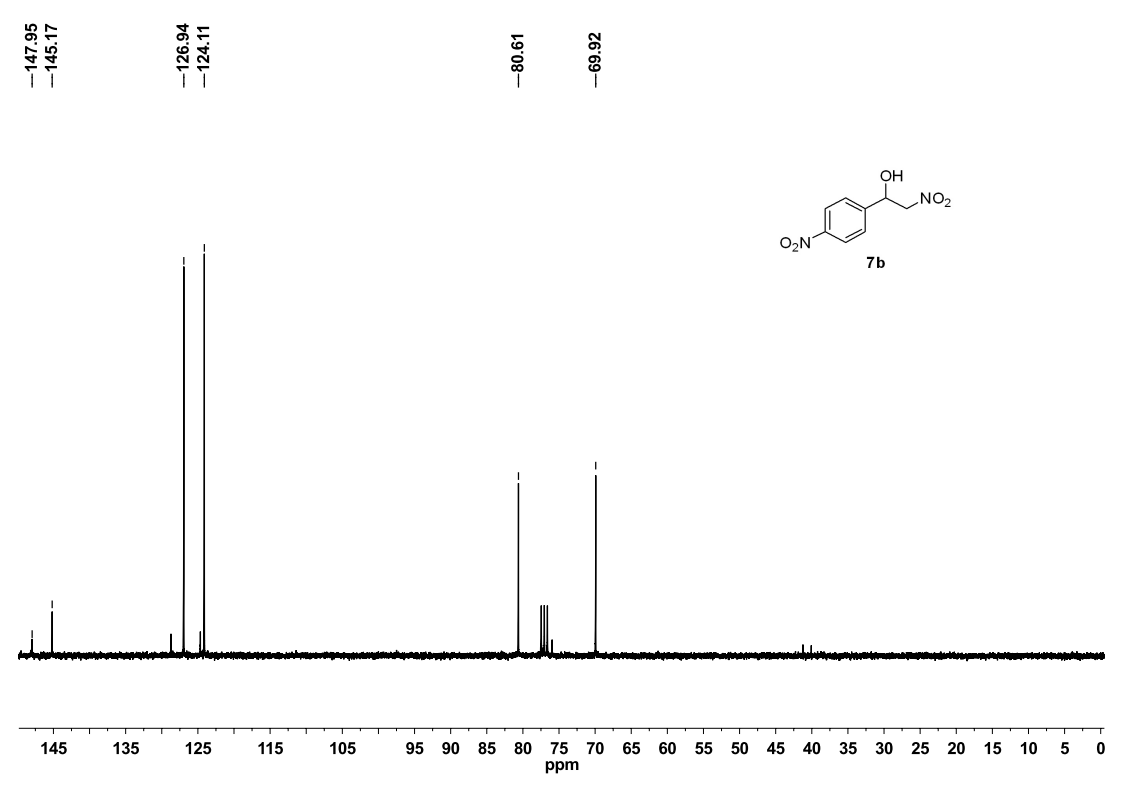


**2-nitro-1-(2-nitrophenyl)ethanol (7c):** [[^11]^](#_ENREF_1)


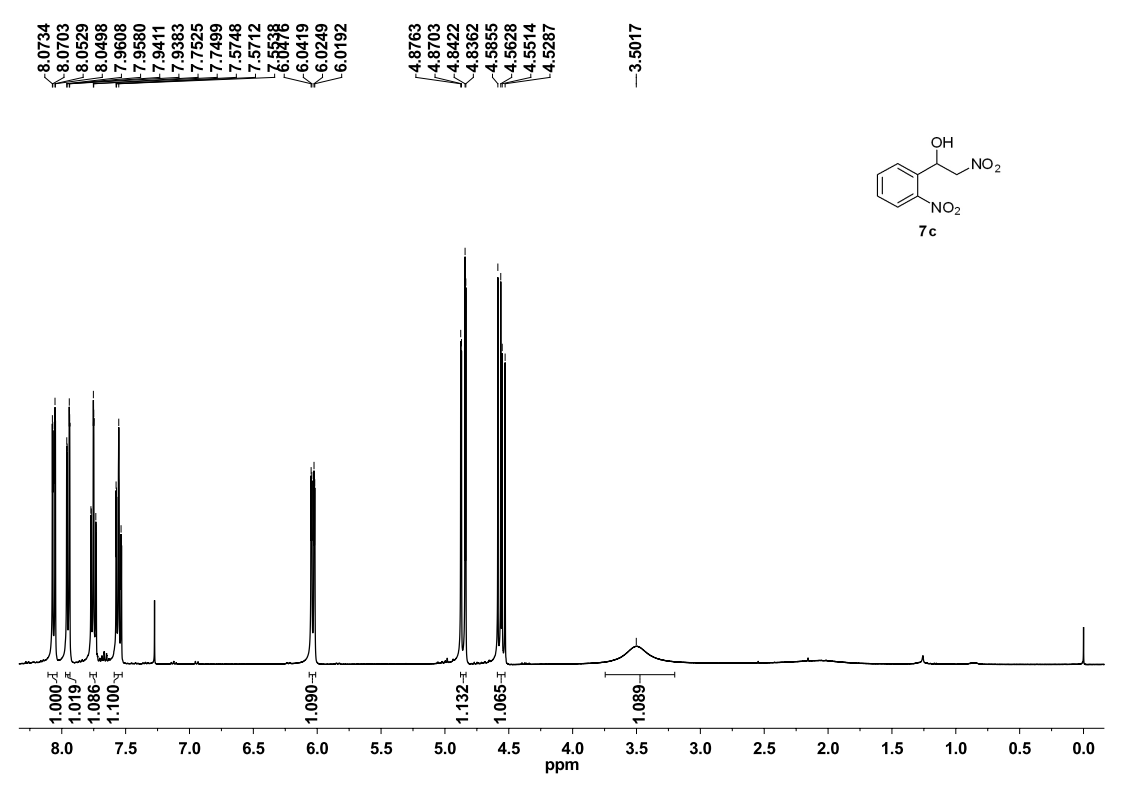


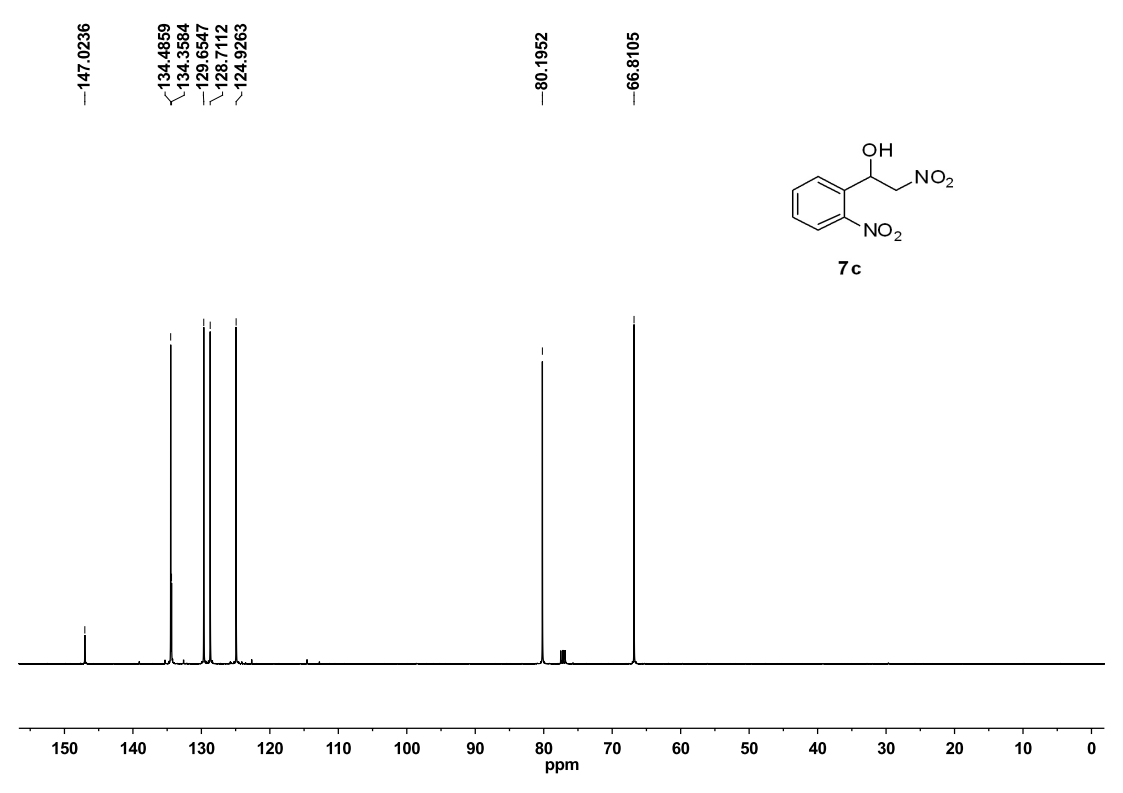


**4-(1-hydroxy-2-nitropropyl)benzonitrile (7d ):** [[^12]^](#_ENREF_1)


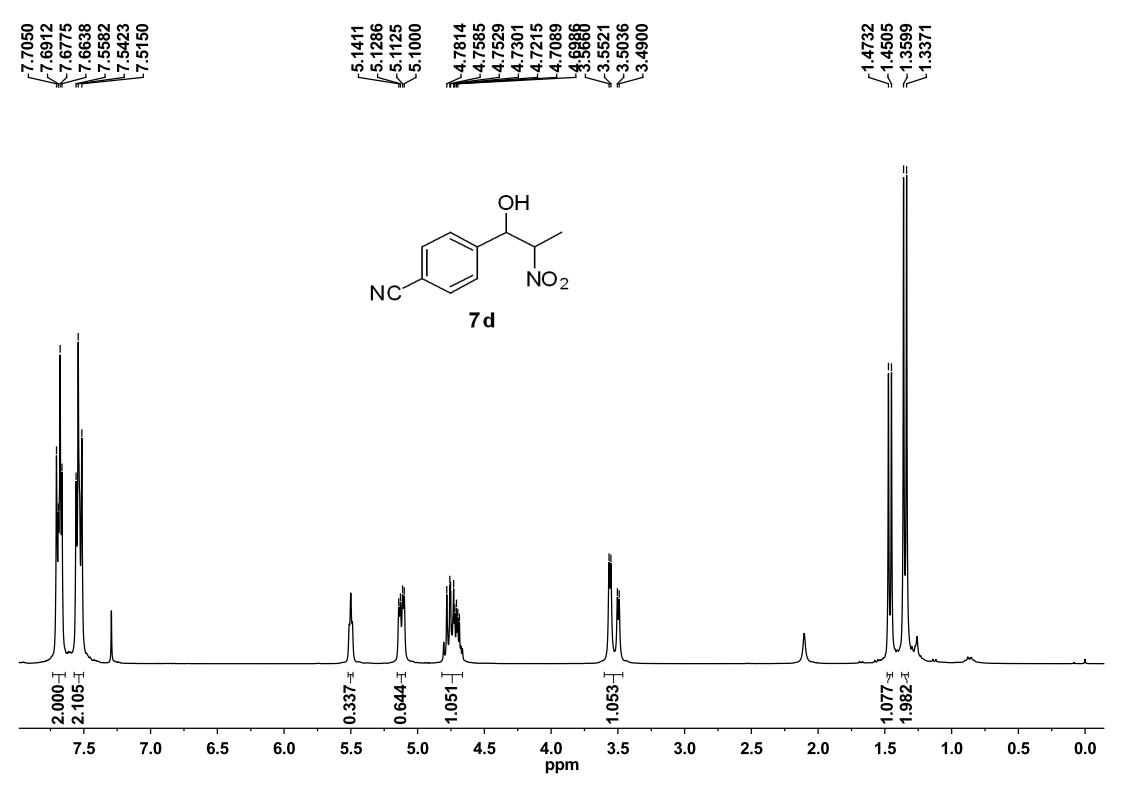


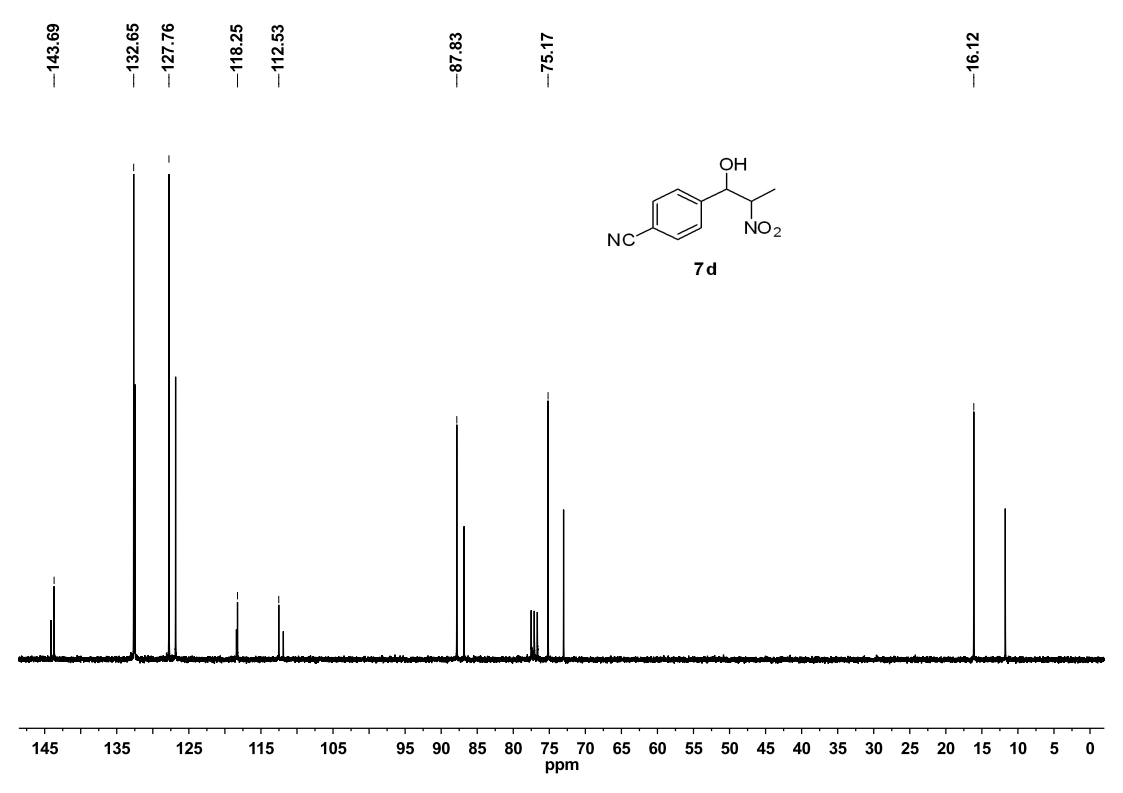


**2-nitro-1-(4-nitrophenyl)butan-1-ol (7e):** [[^10]^](#_ENREF_1)


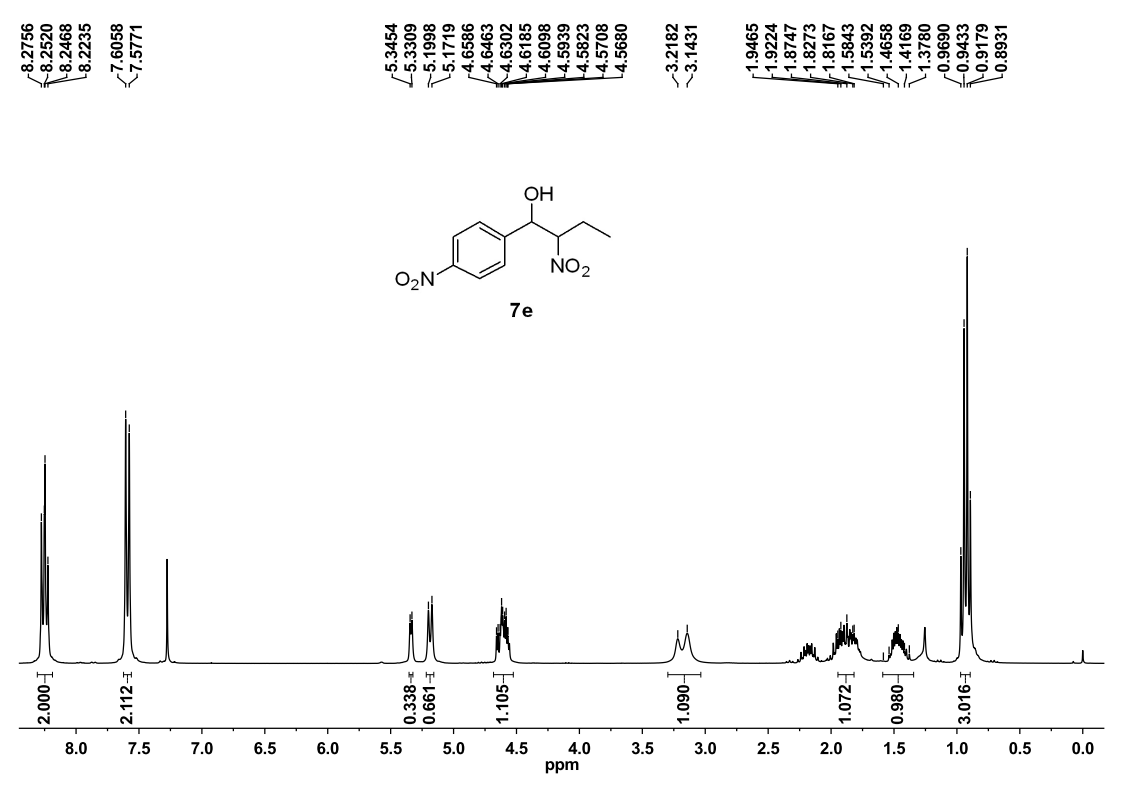


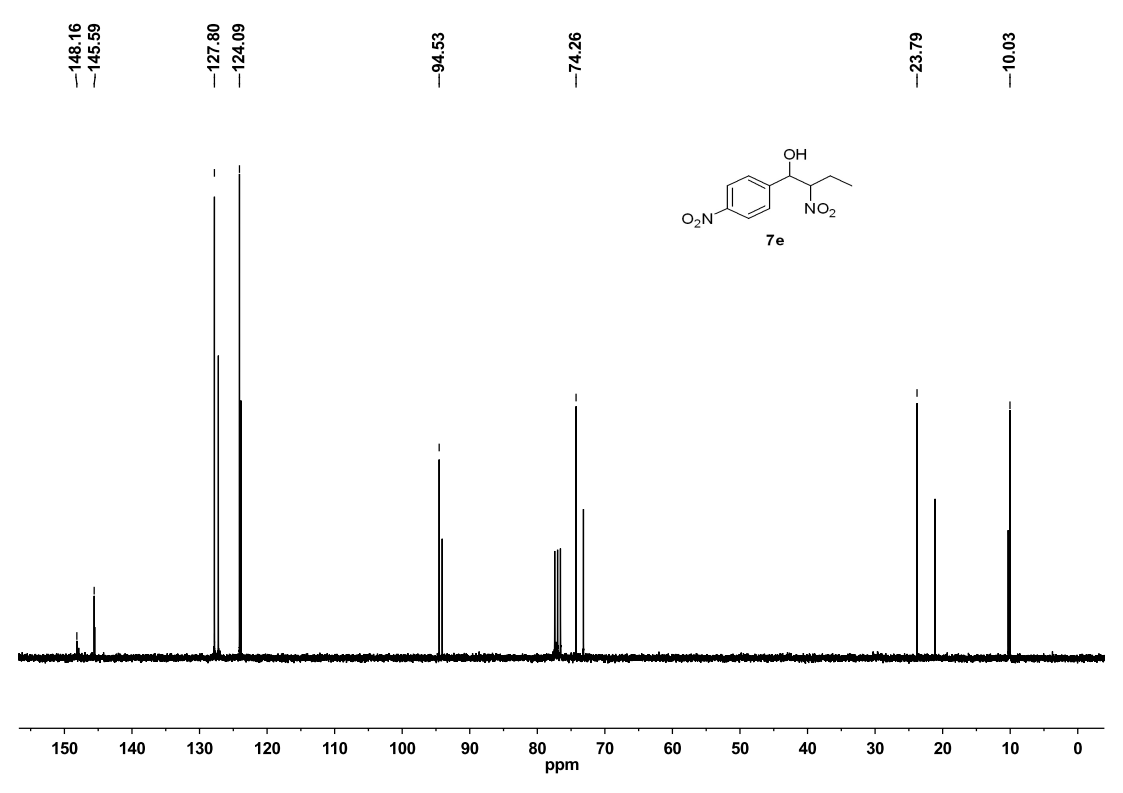


**4-(1-hydroxy-2-nitroethyl)benzonitrile (7f):** [[^10]^](#_ENREF_1)


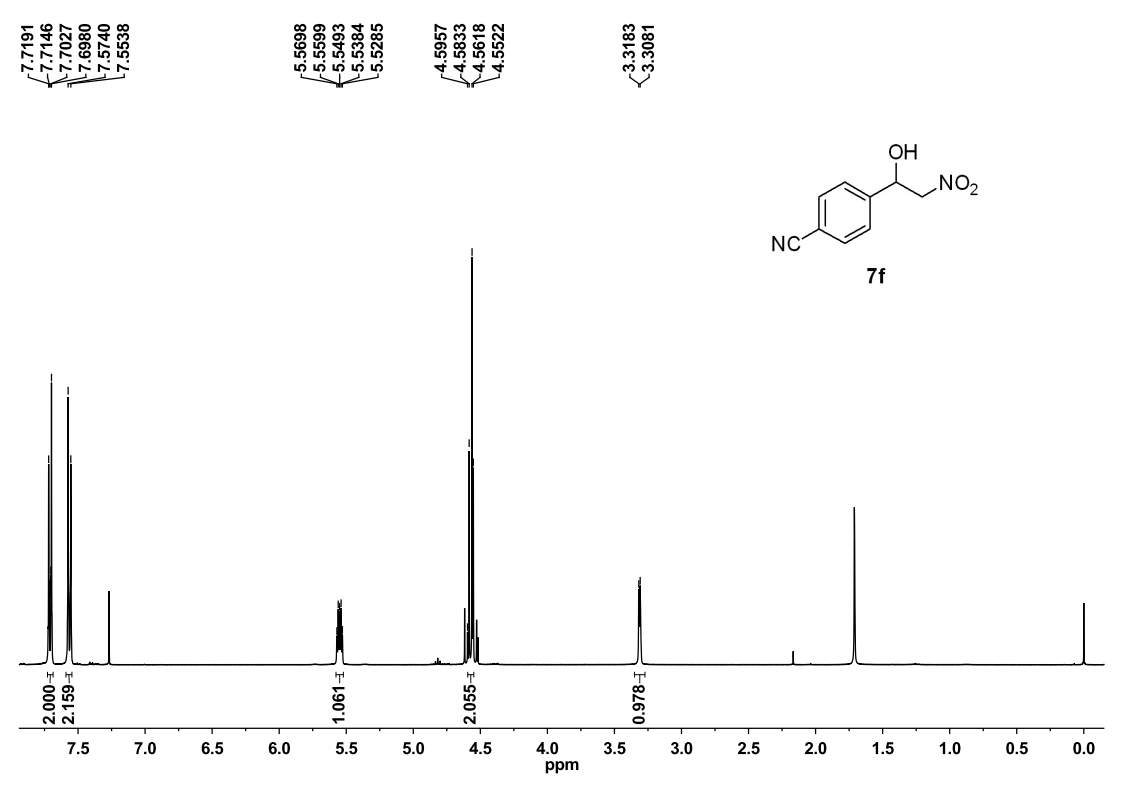


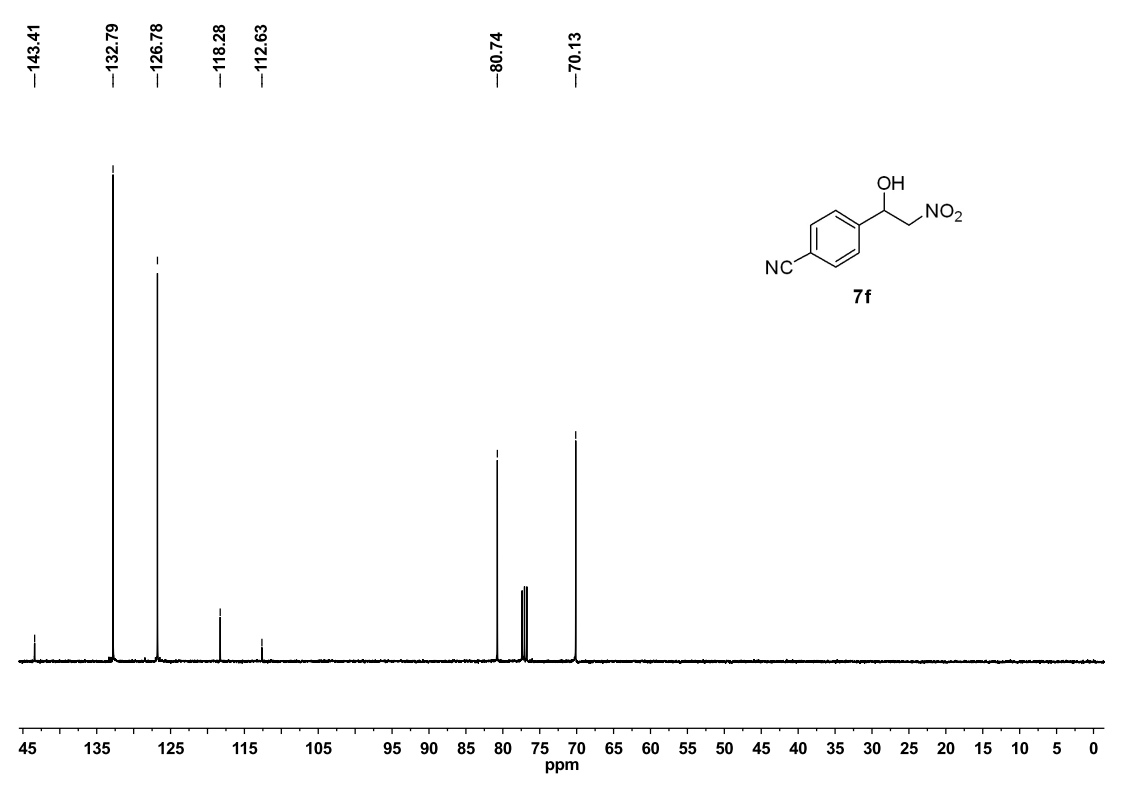


**Biginelli products**

**5-Ethoxycarbonyl-6-methyl-4-(4-methoxyphenyl)-3,4-dihydropyrimidin-2(1H)-one (10a):** ^[13]^


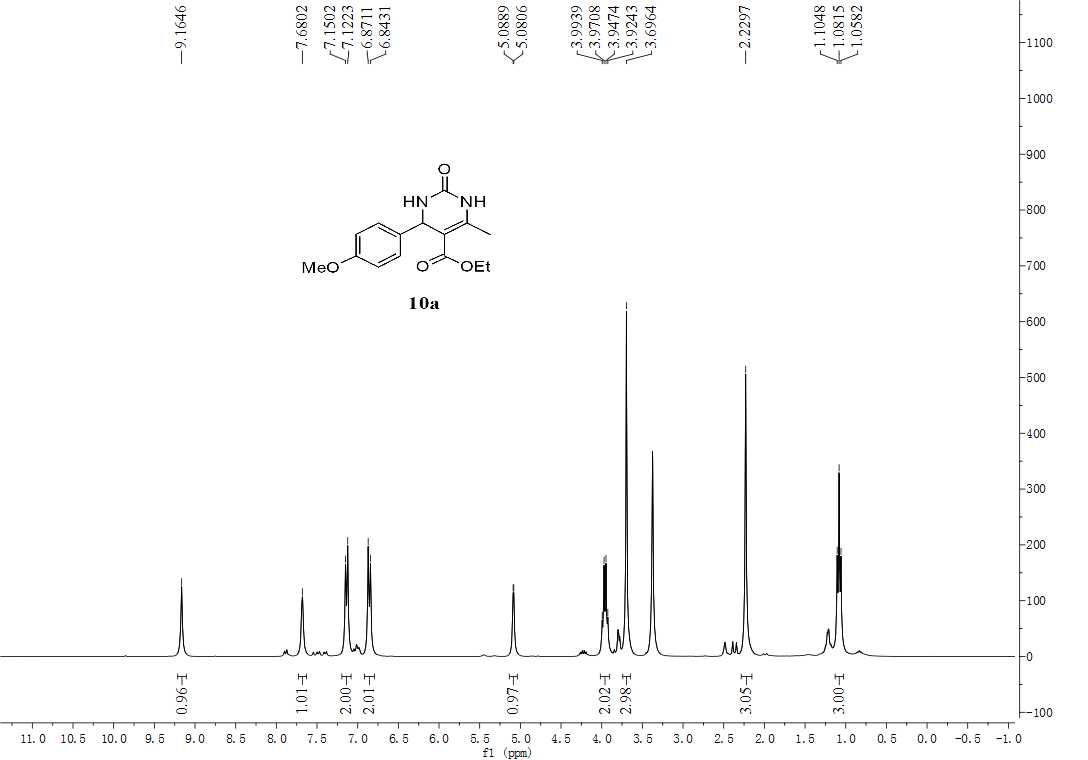


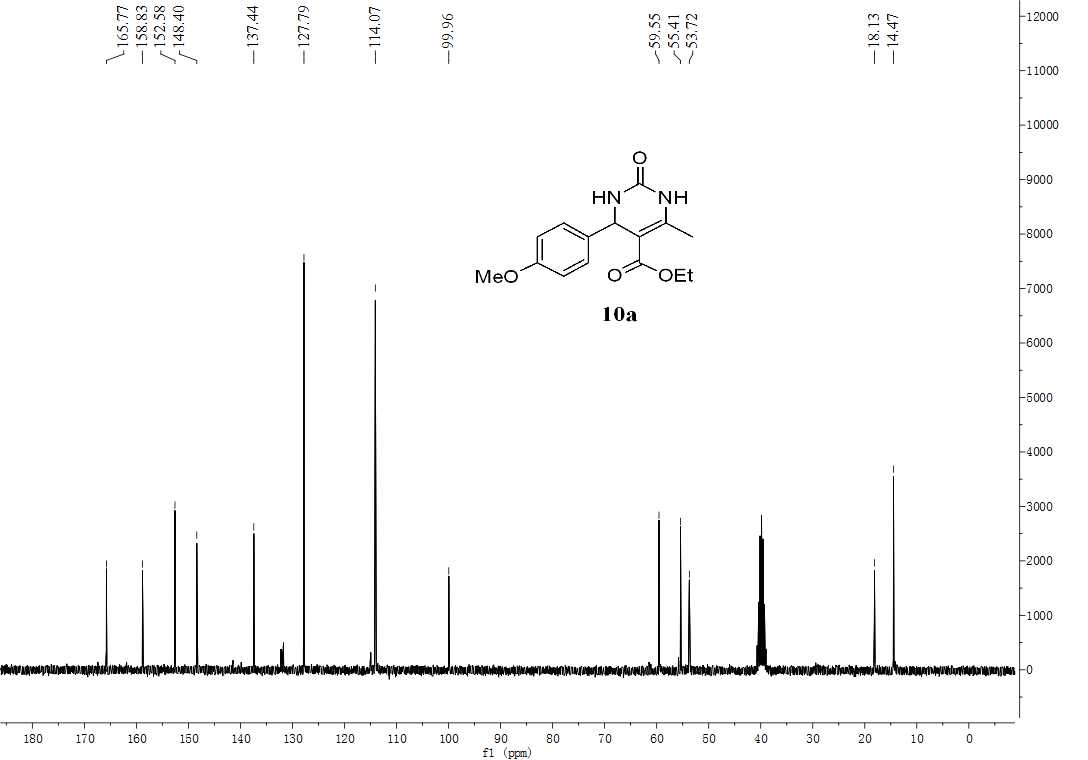


**(R)-5-Ethoxycarbonyl-6-methyl-4-(3-nitrophenyl)-3,4-dihydropyrimidin-2(1H)-one (10b):** ^[14, 15]^


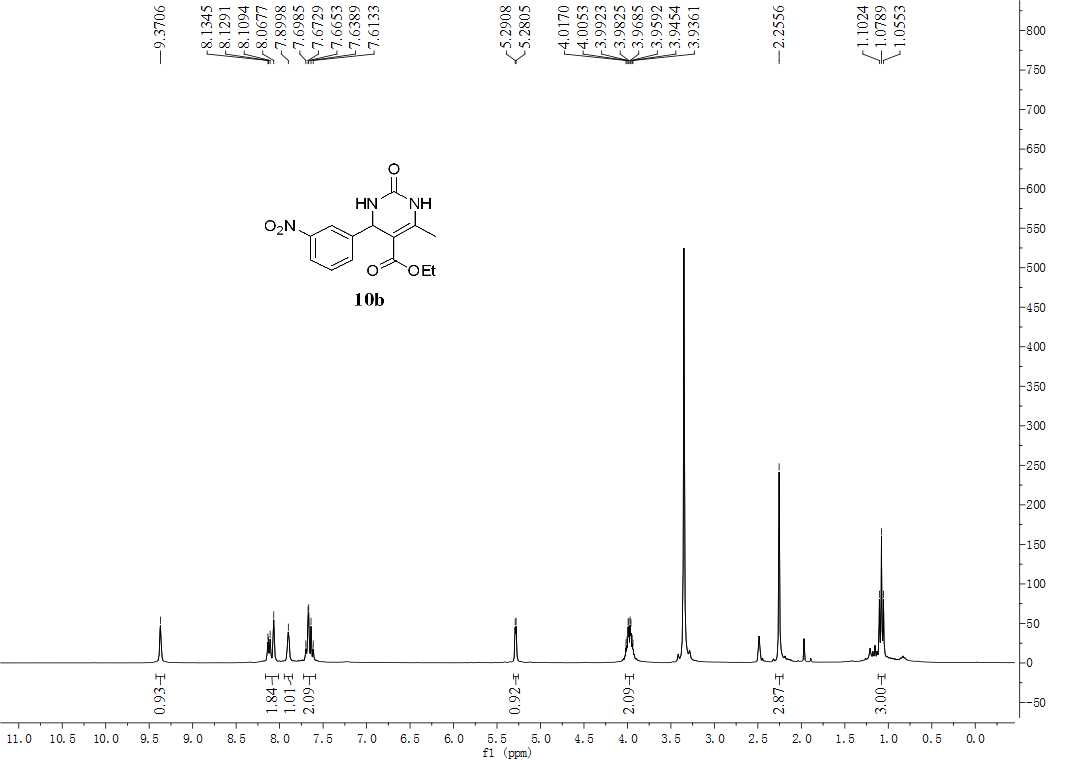


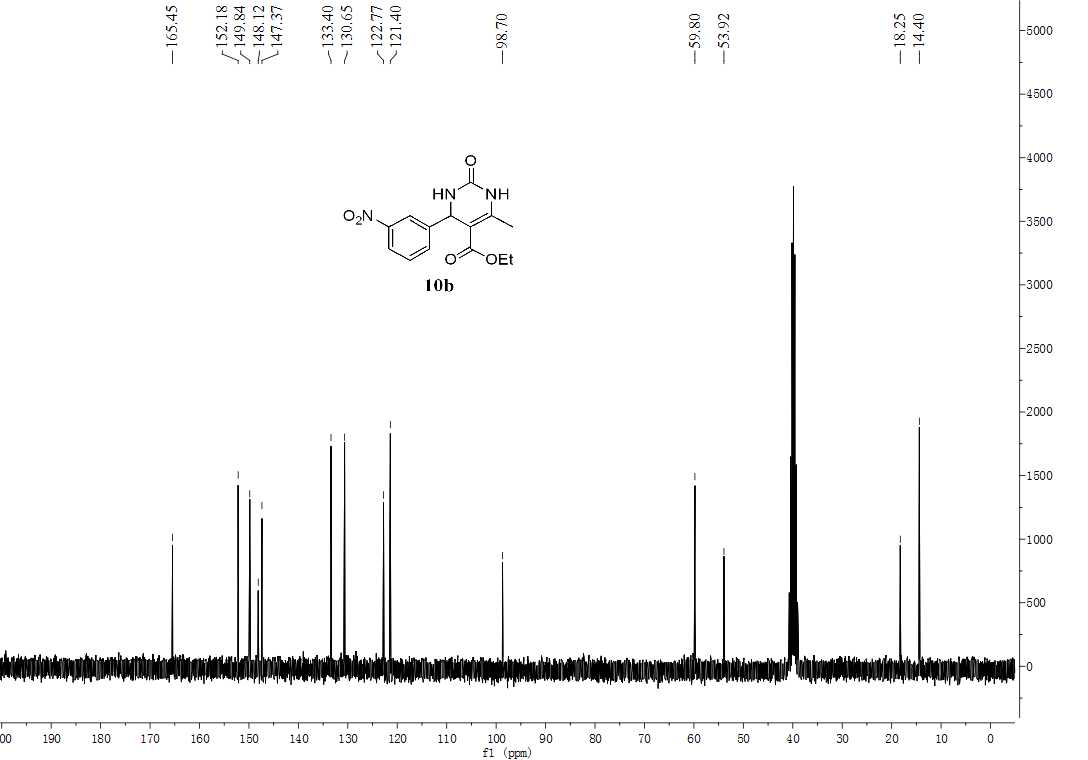


**10b (Racemic)**


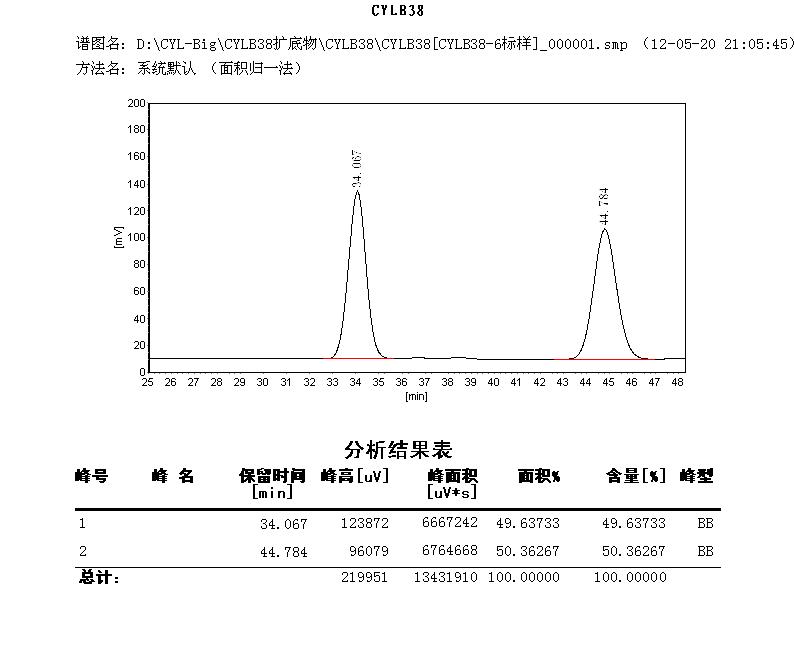
DEFAULT REPORT

| Peak # | Time [min] | Height [μv] | Area [μv.s] | Area [%] |
| --- | --- | --- | --- | --- |
| 1 | 34.067 | 123872 | 6667242 | 49.63733 |
| 2 | 44.784 | 96079 | 6764668 | 50.36267 |
| [Sum](http://www.nciku.cn/search/en/sum) |  | 219951 | 1341910 | 100.00000 |

**10b (Chiral)**


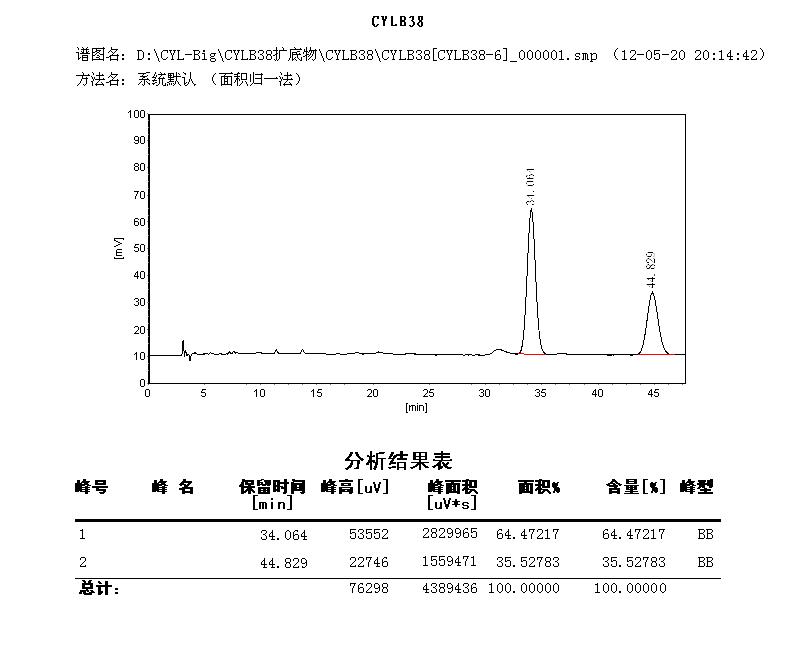
 DEFAULT REPORT

| Peak # | Time [min] | Height [μv] | Area [μv.s] | Area [%] |
| --- | --- | --- | --- | --- |
| 1 | 34.064 | 53552 | 2829965 | 64.47217 |
| 2 | 44.829 | 22746 | 1559471 | 35.52783 |
| [Sum](http://www.nciku.cn/search/en/sum) |  | 76298 | 4389436 | 100.00000 |

**5-Ethoxycarbonyl-6-methyl-4-(4-fluorophenyl)-3,4-dihydropyrimidin-2(1H)-one (10c):** ^[13]^


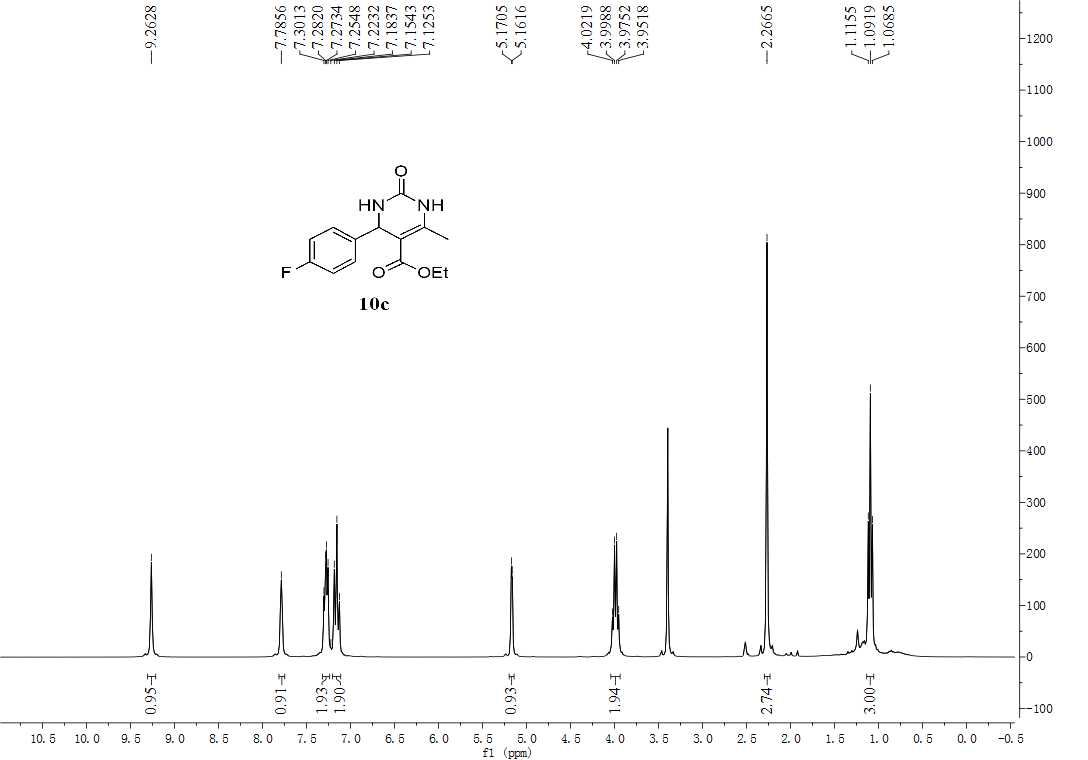


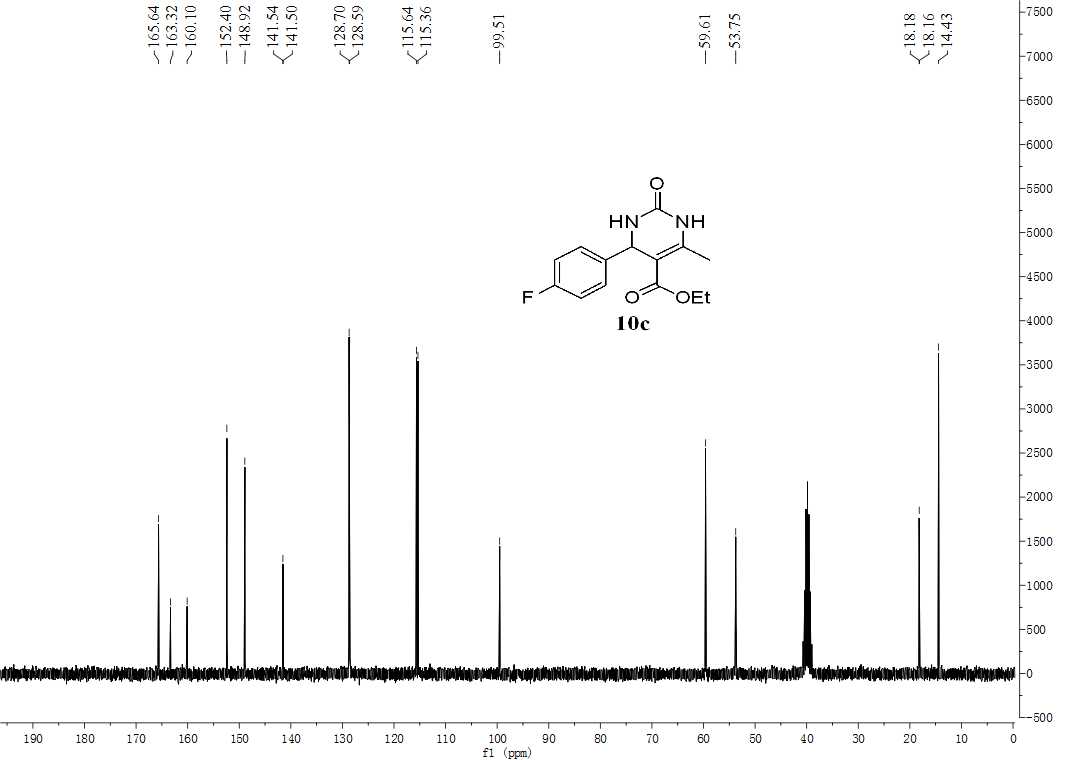


**(R)-5-Ethoxycarbonyl-6-methyl-4-(3-chlorophenyl)-3,4-dihydropyrimidin-2(1H)-one (10d):** ^[14, 16]^


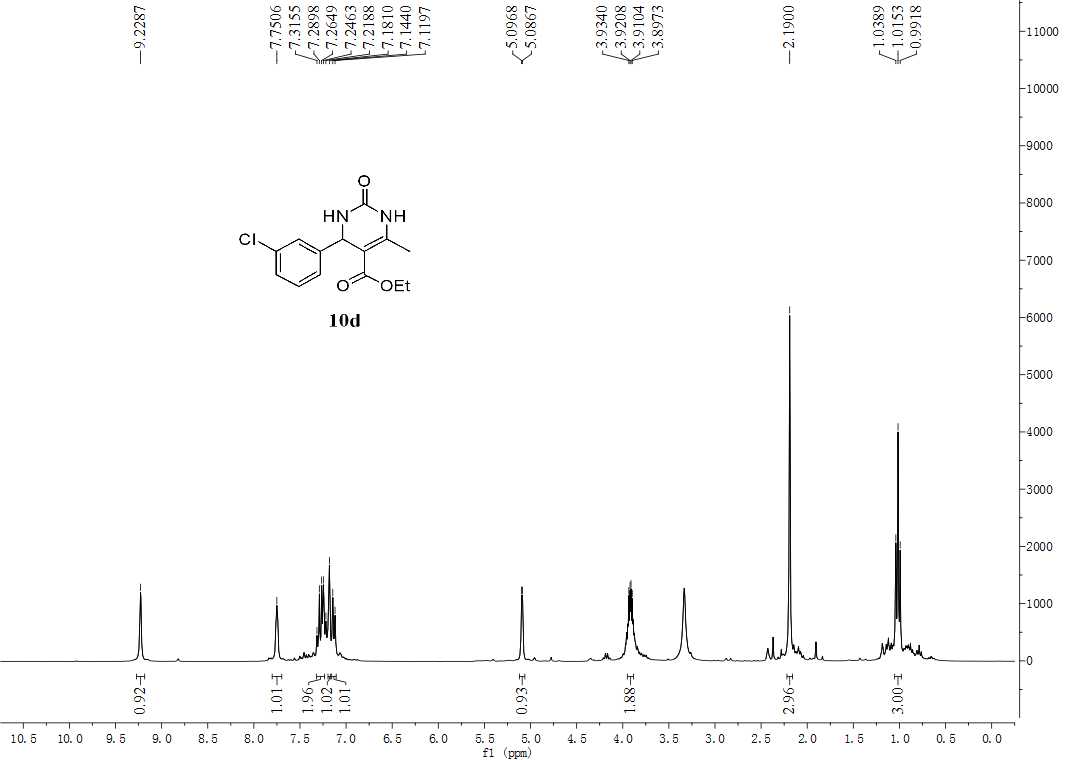


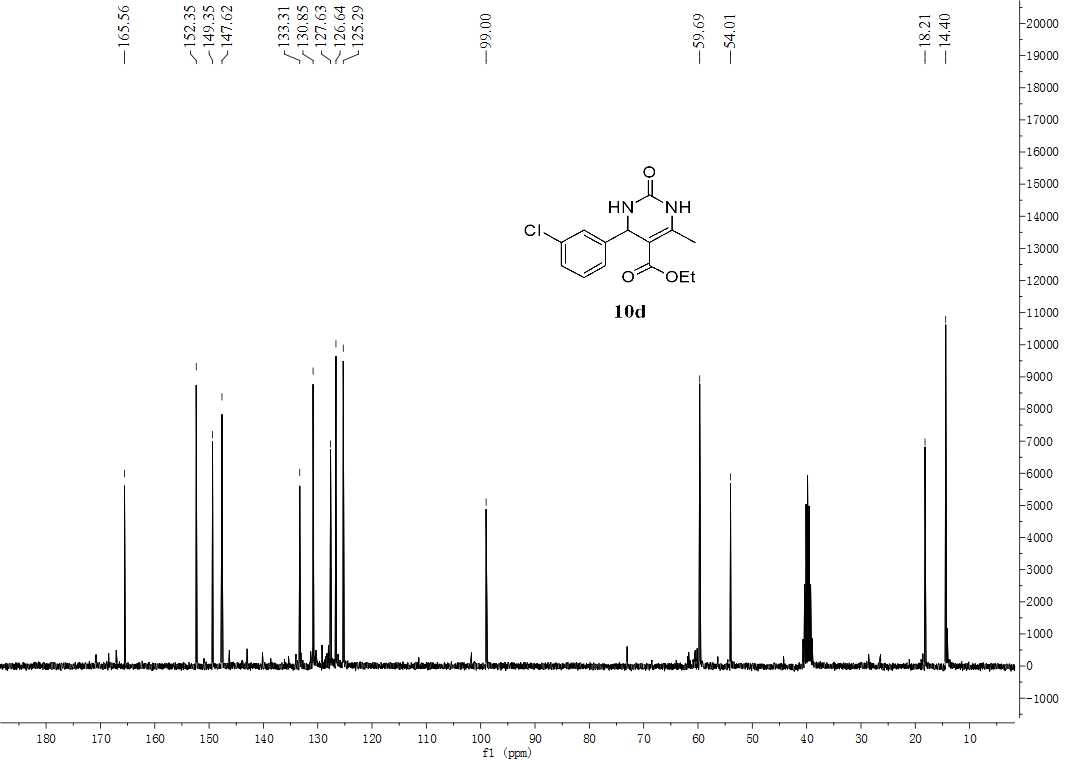


**10d** **(Racemic)**


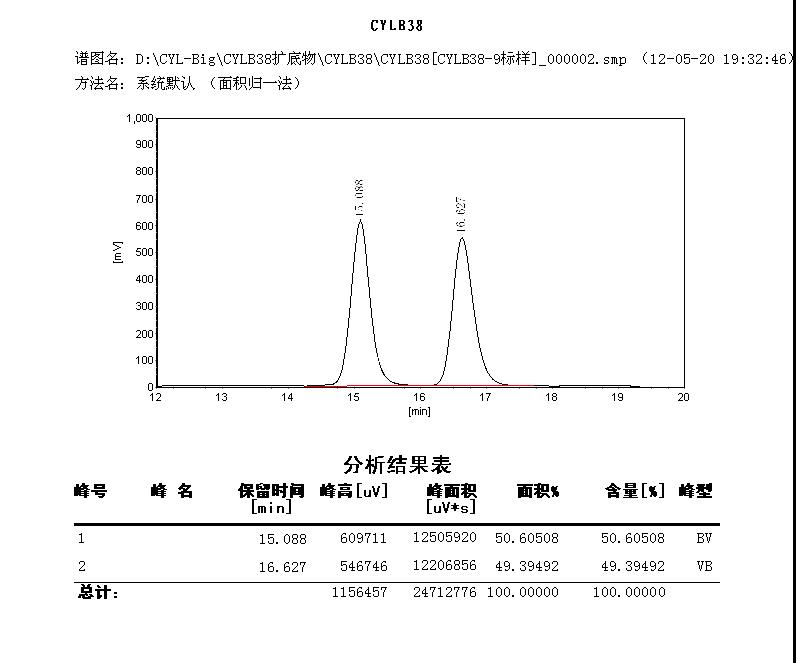
 DEFAULT REPORT

| Peak # | Time [min] | Height [μv] | Area [μv.s] | Area [%] |
| --- | --- | --- | --- | --- |
| 1 | 15.088 | 609711 | 12505920 | 50.60508 |
| 2 | 16.627 | 546746 | 12206856 | 49.39492 |
| [Sum](http://www.nciku.cn/search/en/sum) |  | 1156457 | 24712776 | 100.00000 |

**10d (Chiral)**


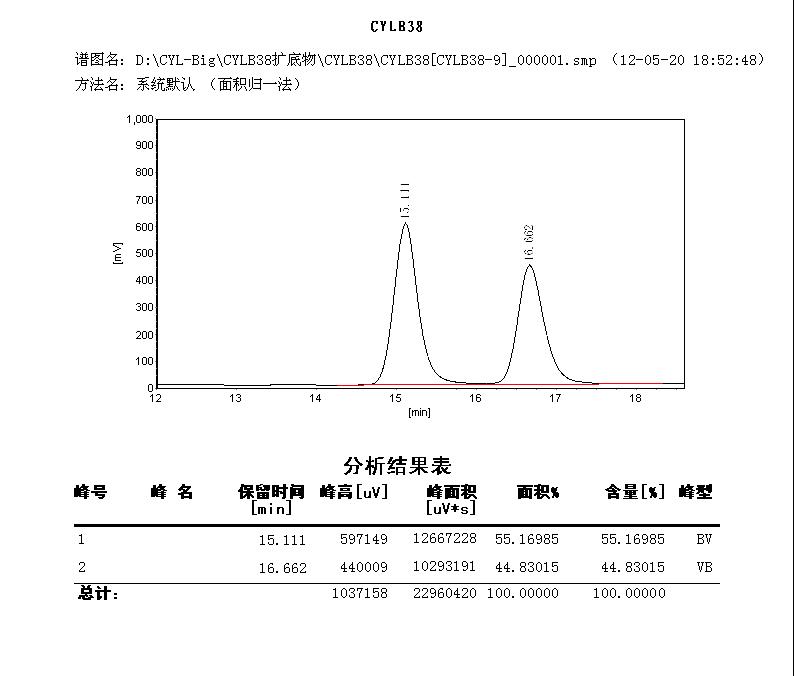
 DEFAULT REPORT

| Peak # | Time [min] | Height [μv] | Area [μv.s] | Area [%] |
| --- | --- | --- | --- | --- |
| 1 | 15.111 | 597149 | 12667228 | 55.16985 |
| 2 | 16.662 | 440009 | 10293191 | 44.83015 |
| [Sum](http://www.nciku.cn/search/en/sum) |  | 1037158 | 22960420 | 100.00000 |

**5-Methoxycarbonyl-6-methyl-4-(2-chlorophenyl)-3,4-dihydropyrimidin-2(1H)-one (10e):** [[^17^](#_ENREF_5)]


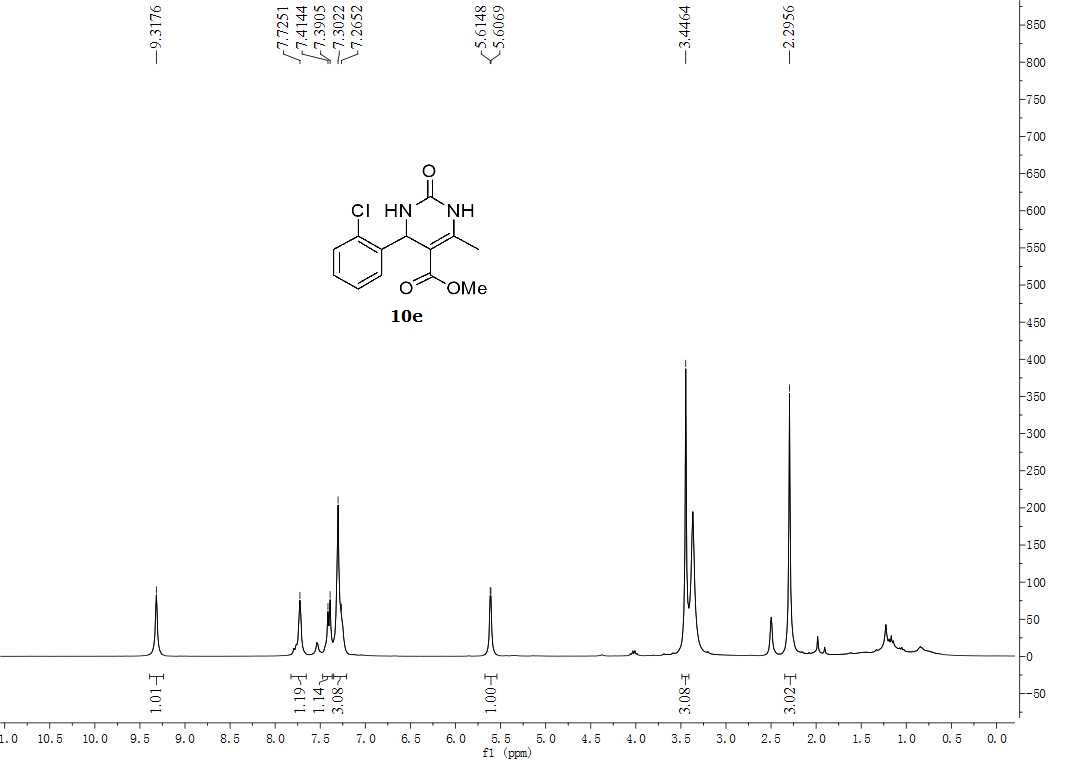


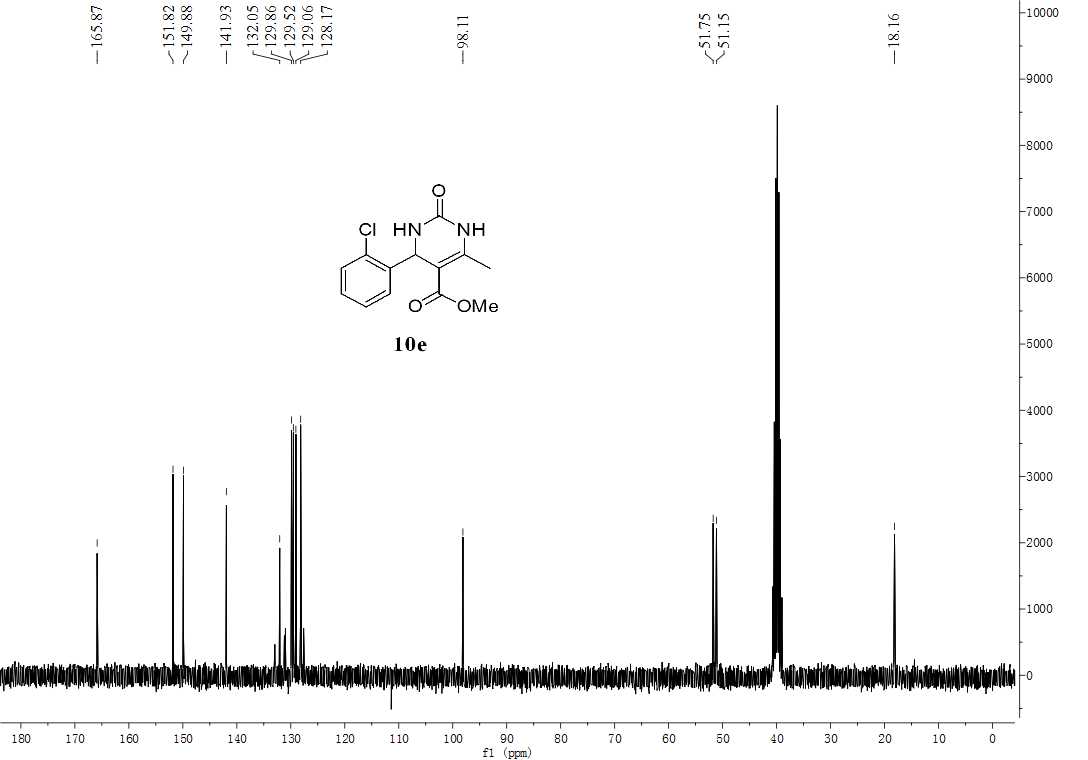


**10e** **(Racemic)**


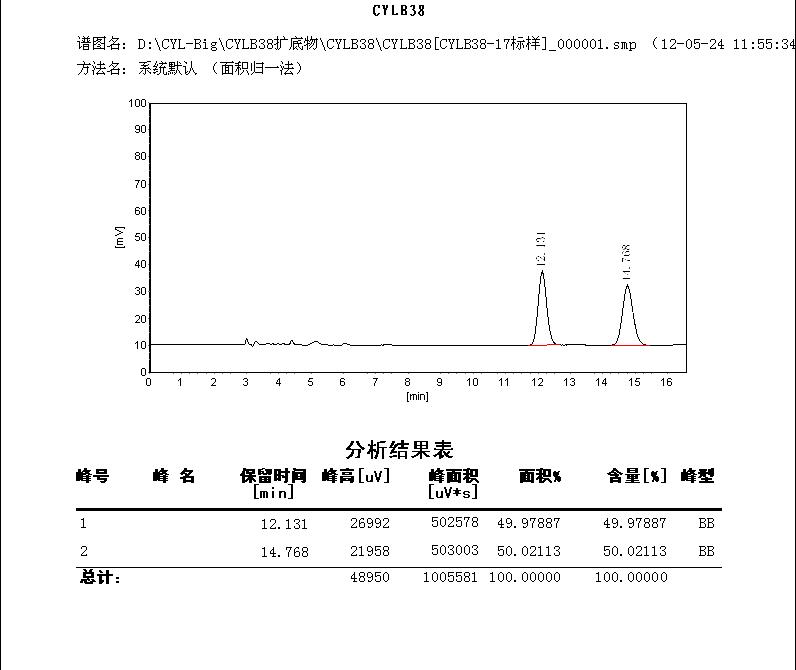
 DEFAULT REPORT

| Peak # | Time [min] | Height [μv] | Area [μv.s] | Area [%] |
| --- | --- | --- | --- | --- |
| 1 | 12.131 | 26992 | 502578 | 49.97887 |
| 2 | 14.768 | 21958 | 503003 | 50.02113 |
| [Sum](http://www.nciku.cn/search/en/sum) |  | 48950 | 1005581 | 100.00000 |

**10e (Chiral)**


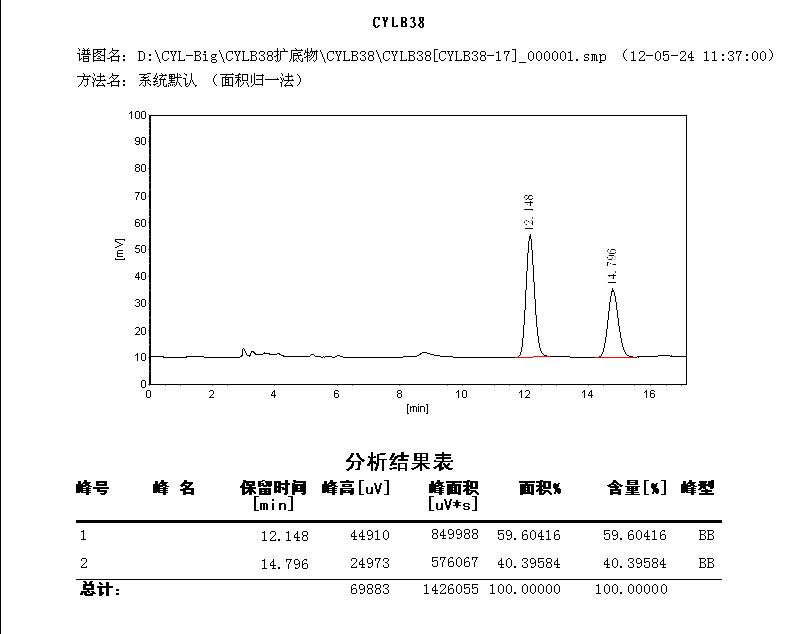
 DEFAULT REPORT

| Peak # | Time [min] | Height [μv] | Area [μv.s] | Area [%] |
| --- | --- | --- | --- | --- |
| 1 | 12.148 | 44910 | 849988 | 59.60416 |
| 2 | 14.796 | 24973 | 576067 | 40.39584 |
| [Sum](http://www.nciku.cn/search/en/sum) |  | 69883 | 1426055 | 100.00000 |

**5-Methoxycarbonyl-6-methyl-4-(3-nitrophenyl)-3,4-dihydropyrimidin-2(1H)-one (10f):** ^[17]^


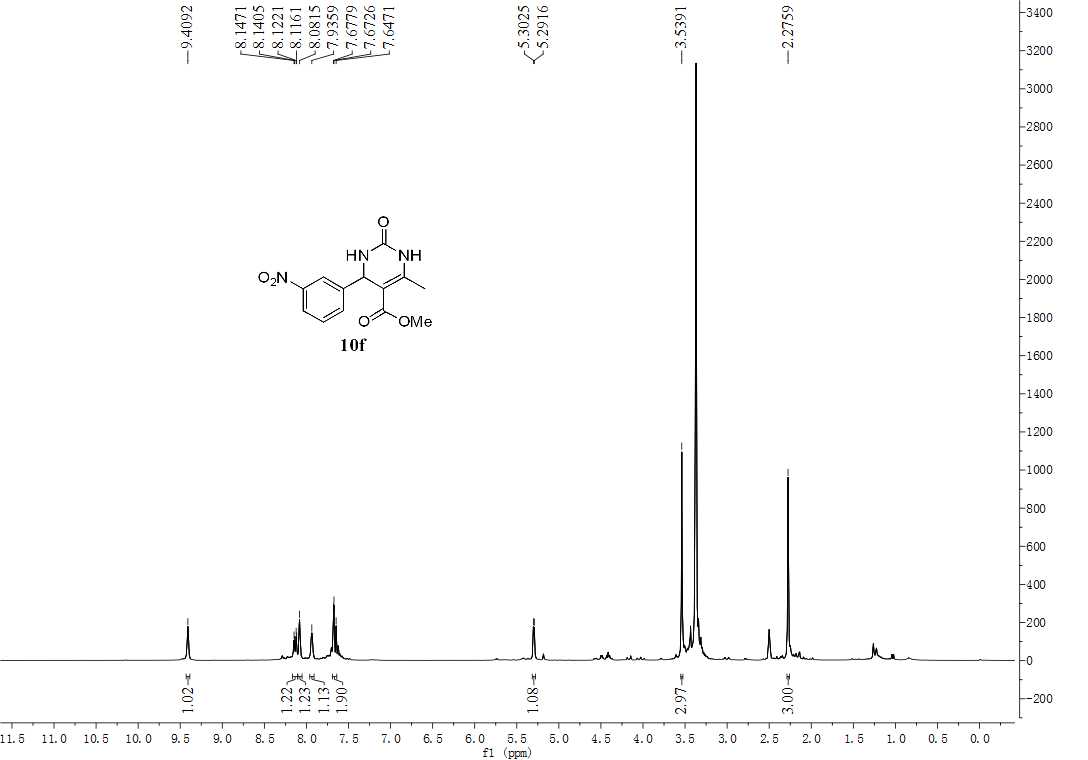


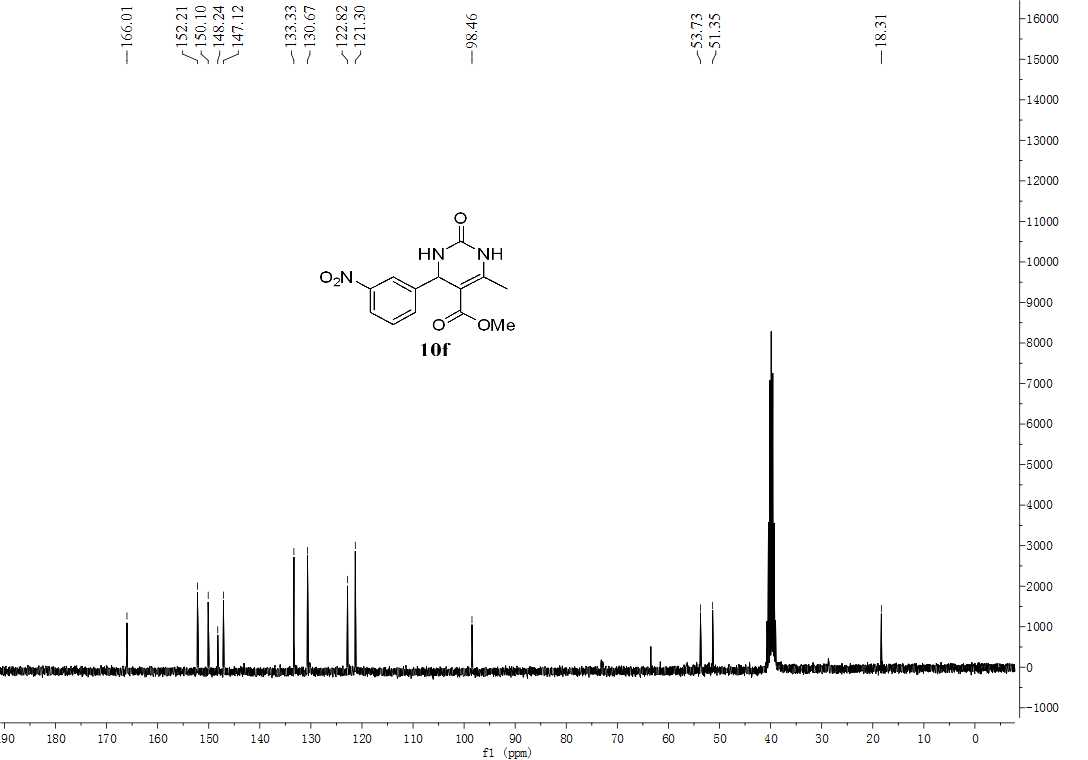


**10f** **(Racemic)**


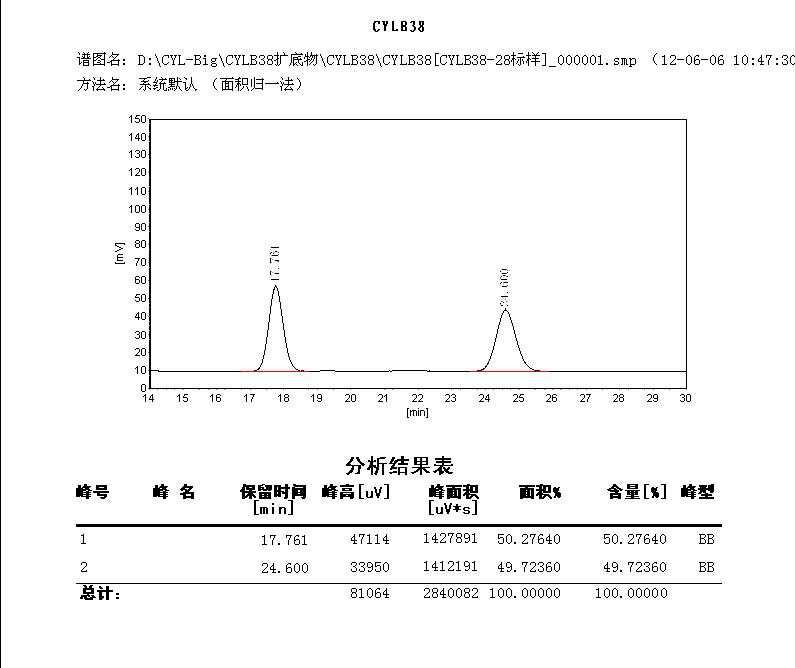
 DEFAULT REPORT

| Peak # | Time [min] | Height [μv] | Area [μv.s] | Area [%] |
| --- | --- | --- | --- | --- |
| 1 | 17.761 | 47114 | 1427891 | 50.27640 |
| 2 | 24.600 | 33950 | 1412191 | 49.72360 |
| [Sum](http://www.nciku.cn/search/en/sum) |  | 81064 | 2840082 | 100.00000 |

**10f (Chiral)**


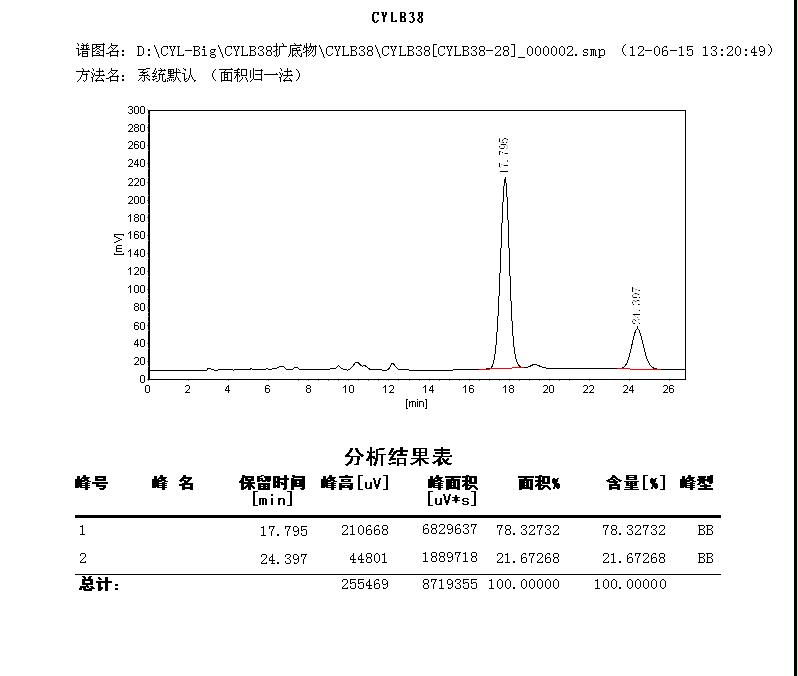


DEFAULT REPORT

| Peak # | Time [min] | Height [μv] | Area [μv.s] | Area [%] |
| --- | --- | --- | --- | --- |
| 1 | 17.795 | 210668 | 6829637 | 78.32732 |
| 2 | 24.397 | 44801 | 1889718 | 21.27268 |
| [Sum](http://www.nciku.cn/search/en/sum) |  | 255469 | 8719355 | 100.00000 |

**5-Ethoxycarbonyl-6-methyl-4-phenyl-3,4-dihydropyrimidin-2(1H)-one (10g):** [[^18^](#_ENREF_6)]


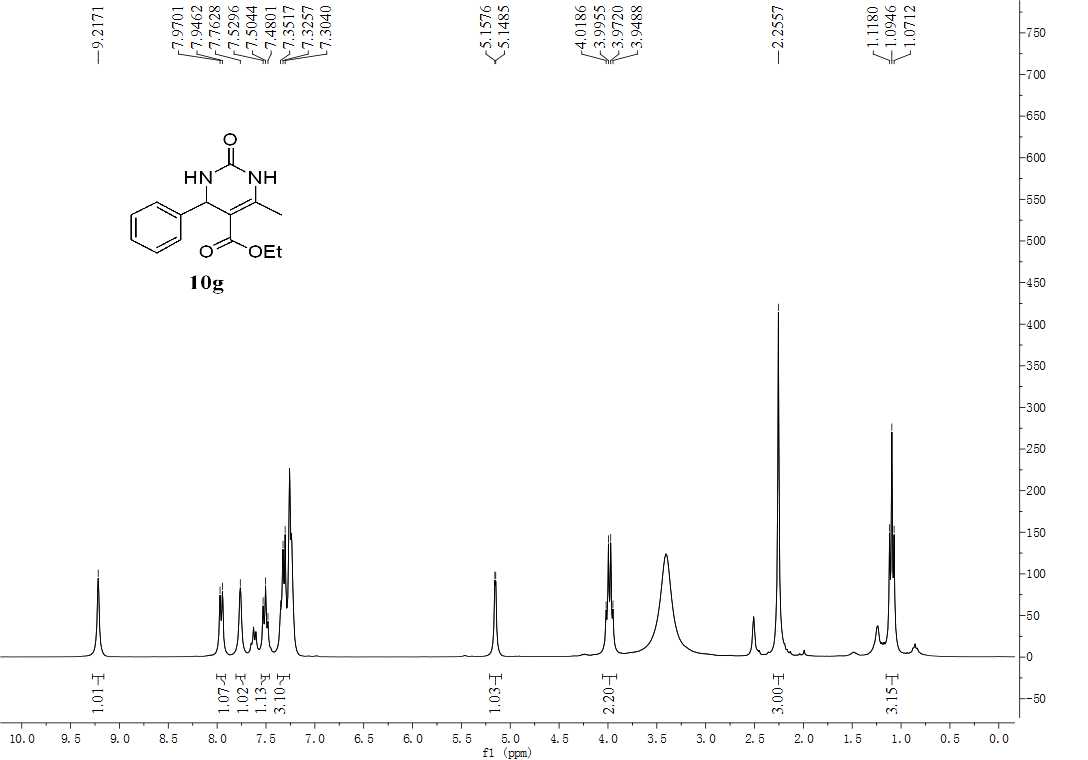


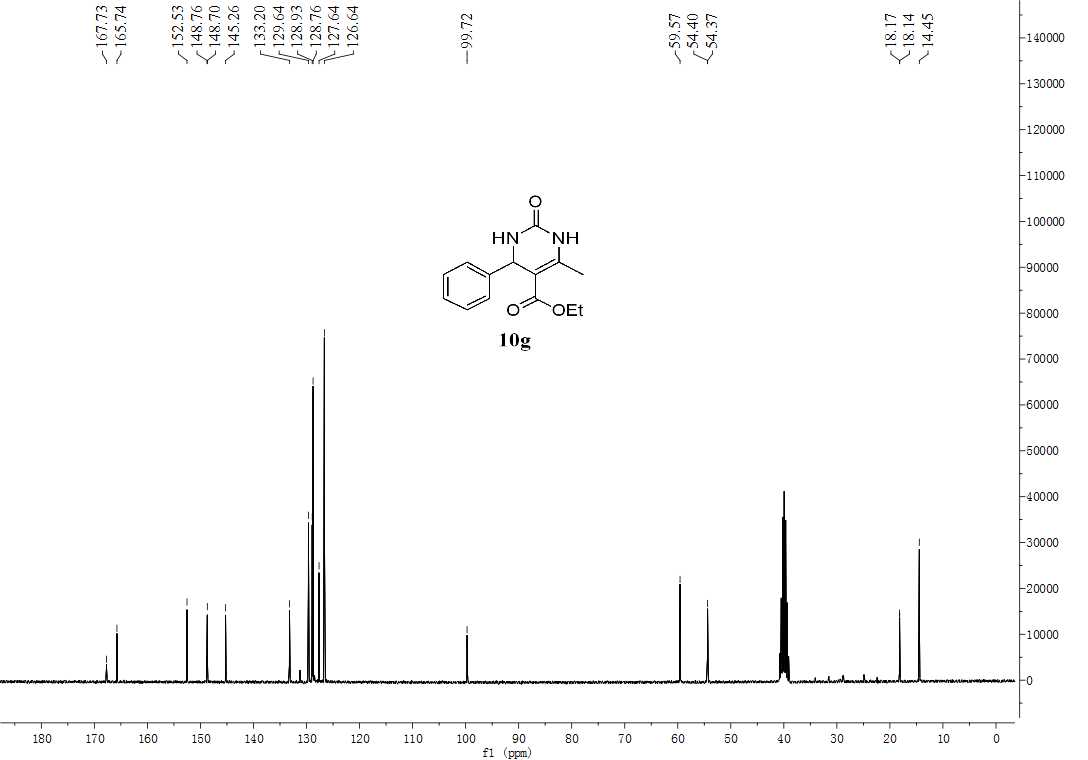


**Coumarin derivatives**

**3-benzoyl-6-nitro-2*H*-chromen-2-one (13a):** [[^19^](#_ENREF_6)]


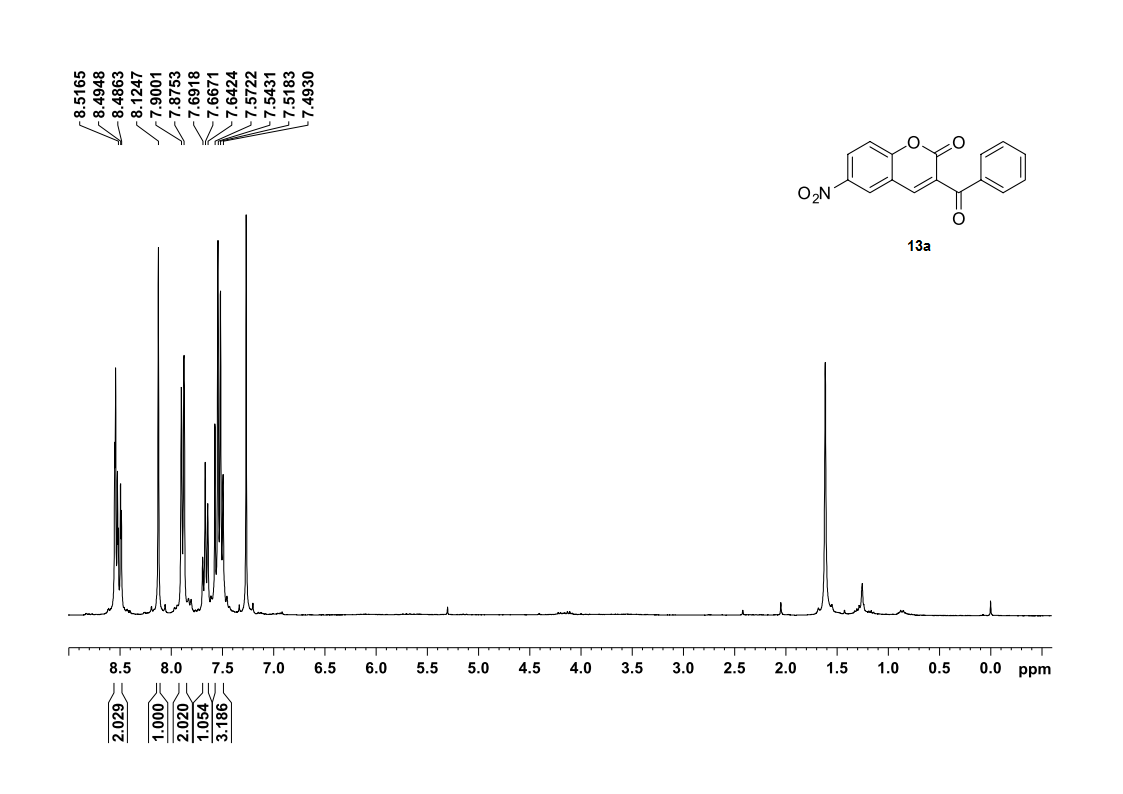


**
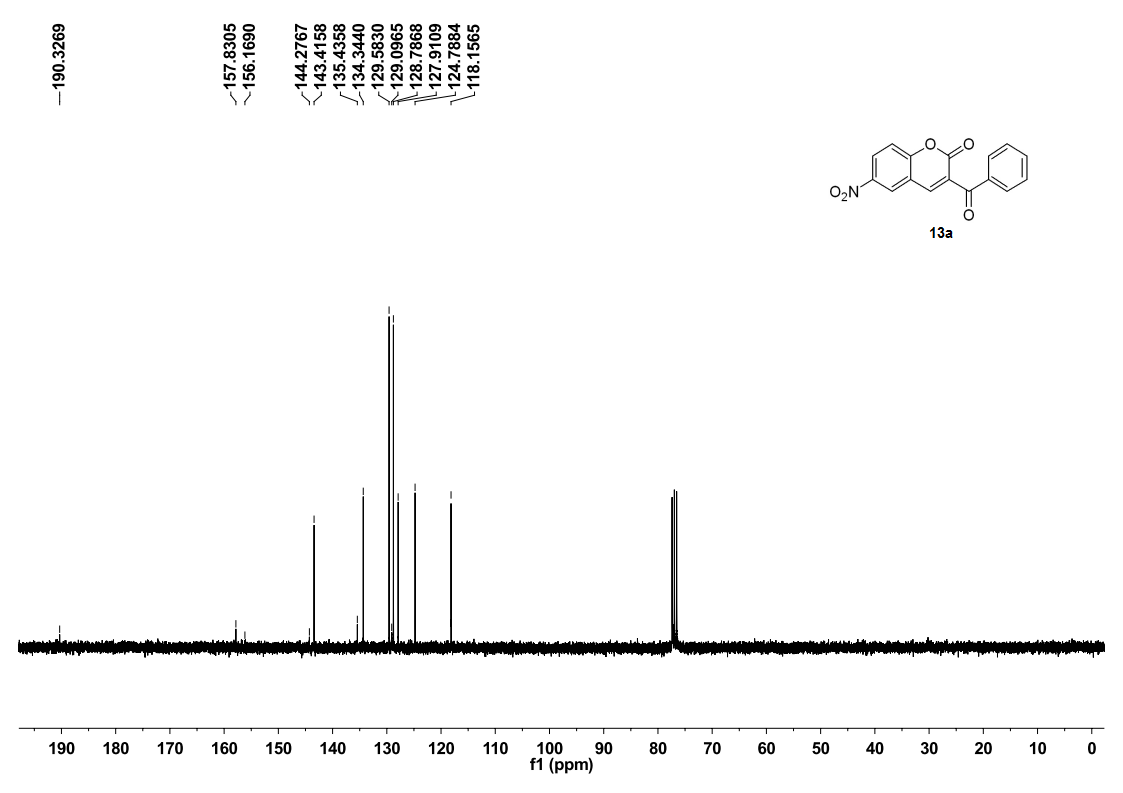
**

**3-Benzoyl-6-chloro-2*H*-chromen-2-one (13b):** [[^20^](#_ENREF_6)]


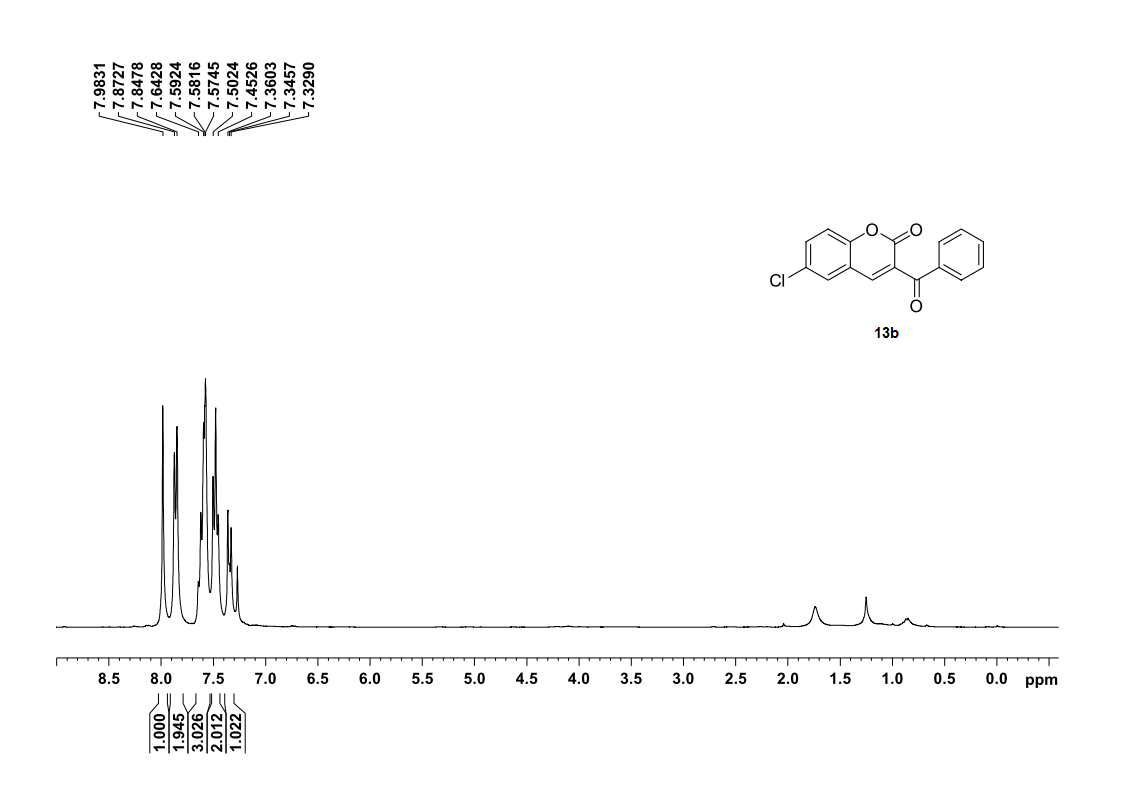


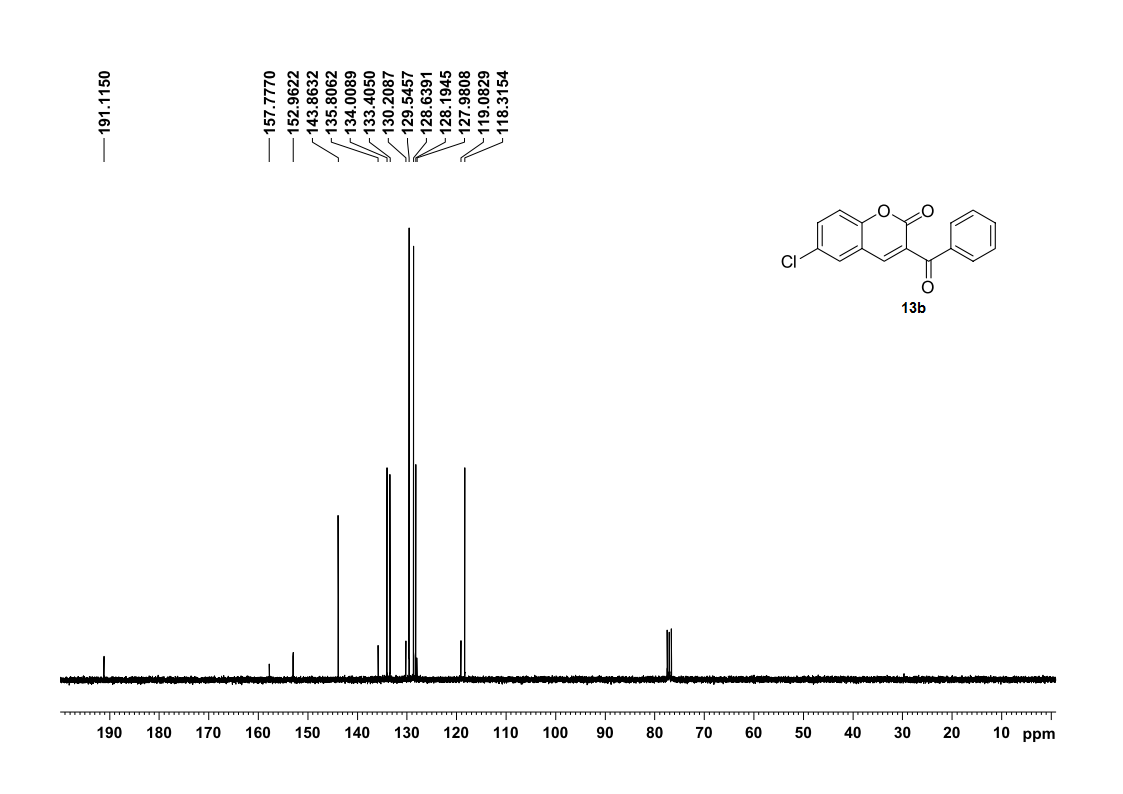


**3-Benzoyl-6-methoxy-2*H*-chromen-2-one (13c):** [[^20^](#_ENREF_6)]


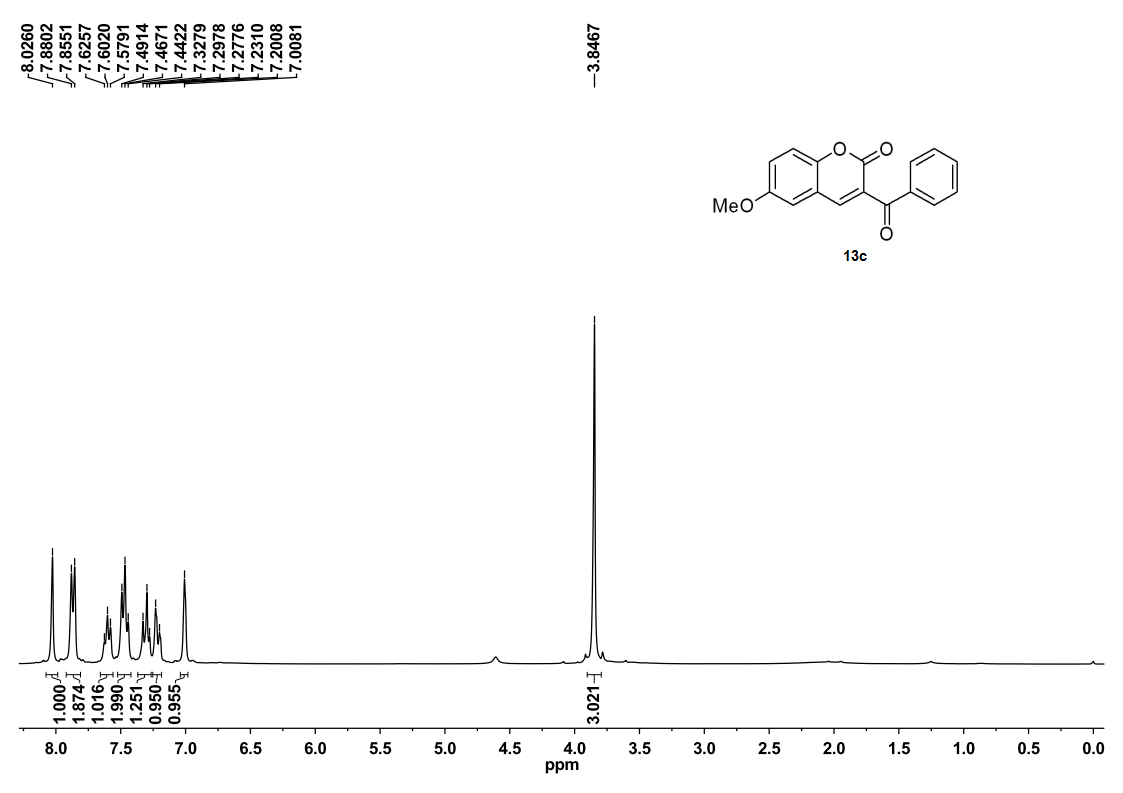


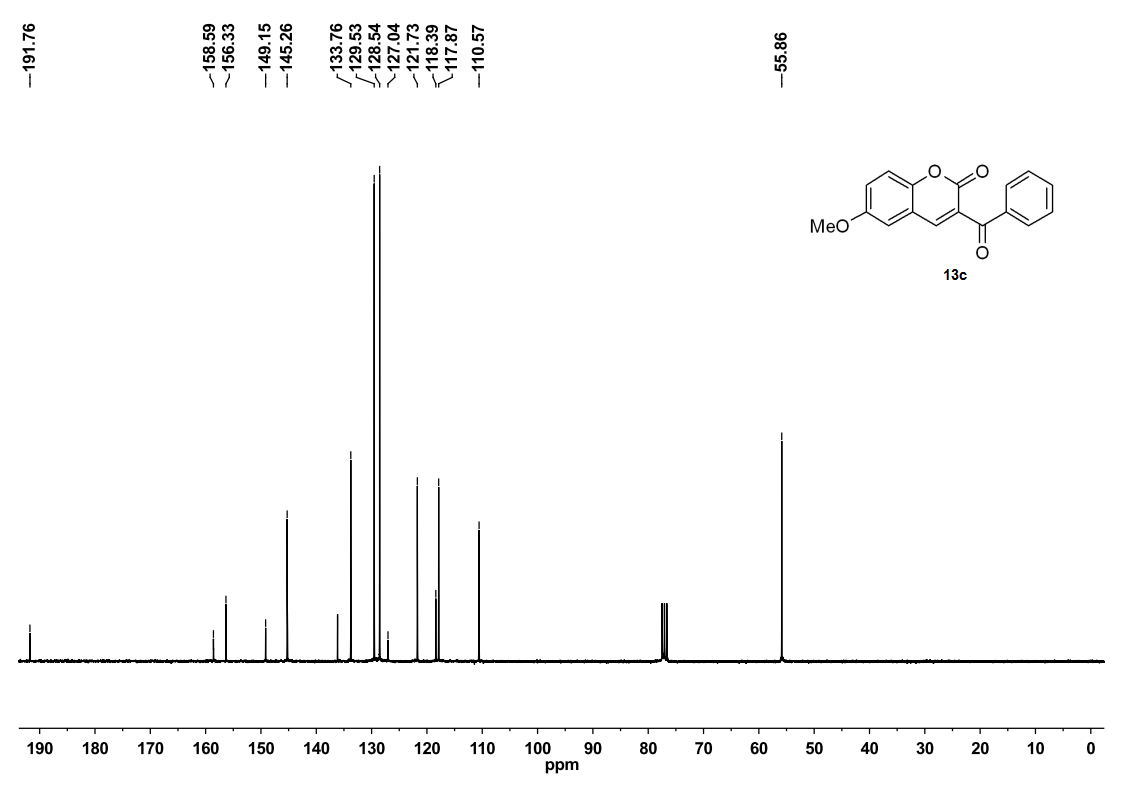


**2-benzoyl-3*H*-benzo[f]chromen-3-one (13d):** [[^21^](#_ENREF_6)]


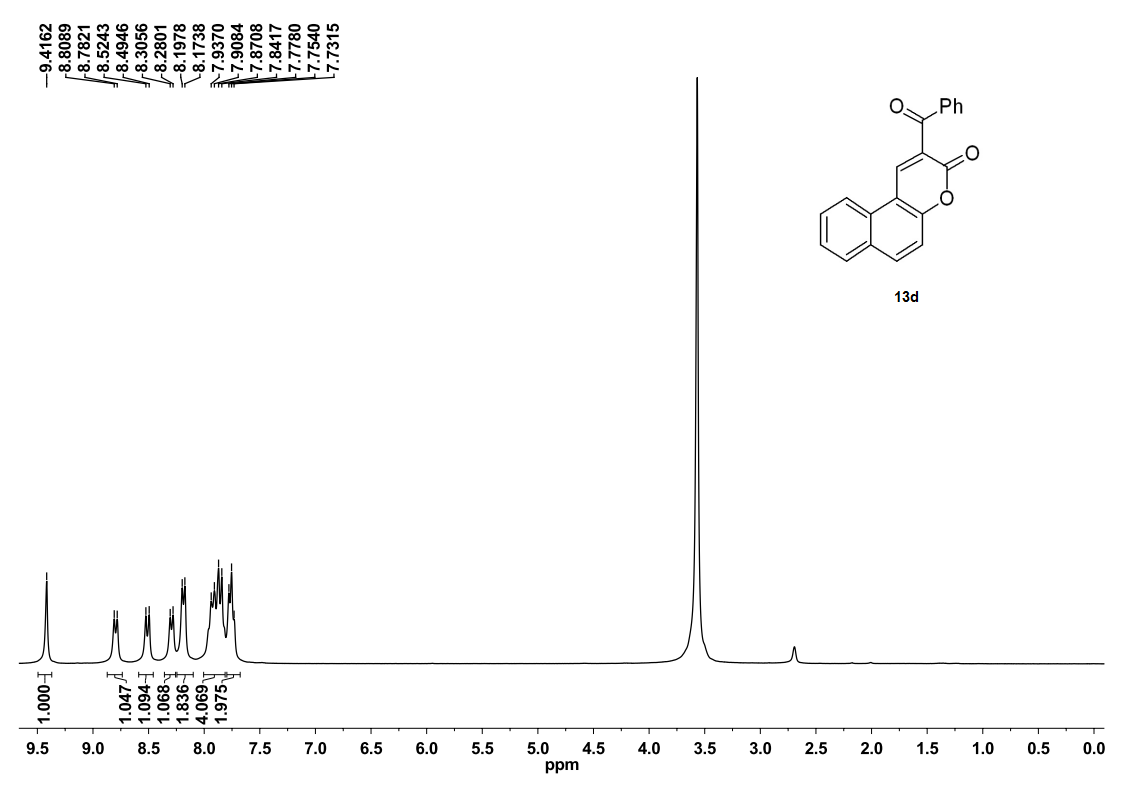


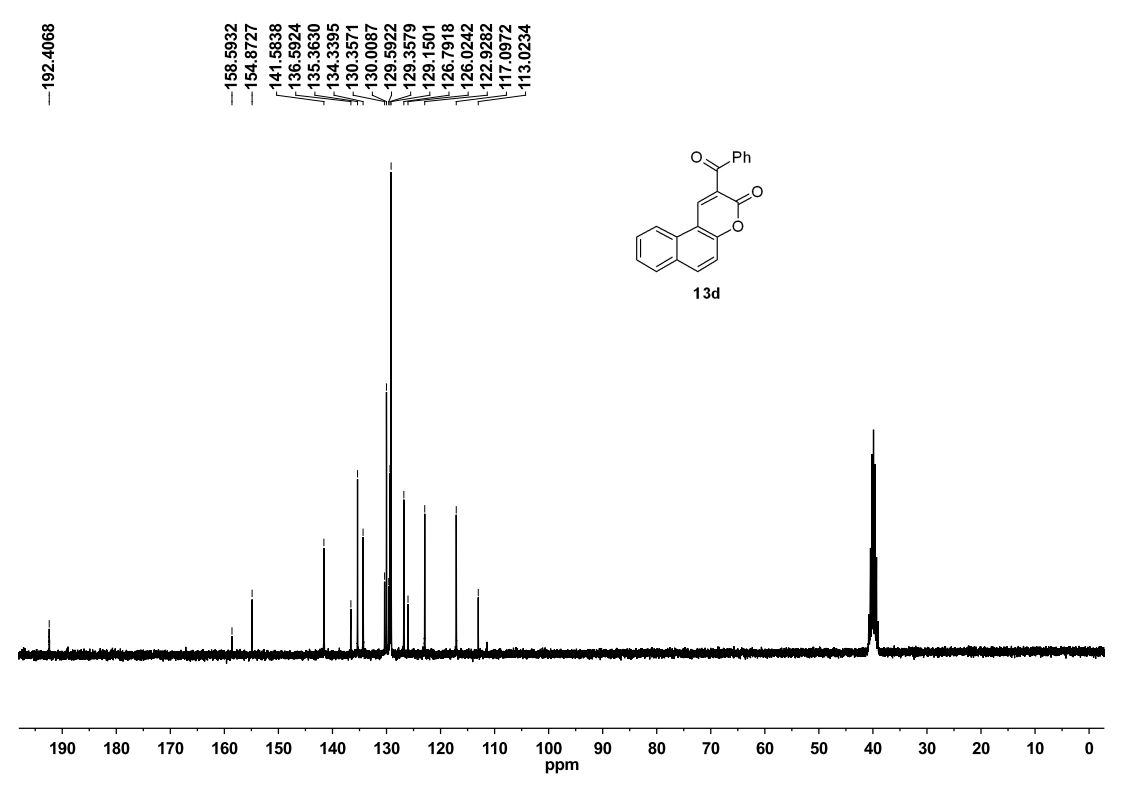


**3-Benzoyl-2*H*-chromen-2-one (13e):** [[^20^](#_ENREF_6)]


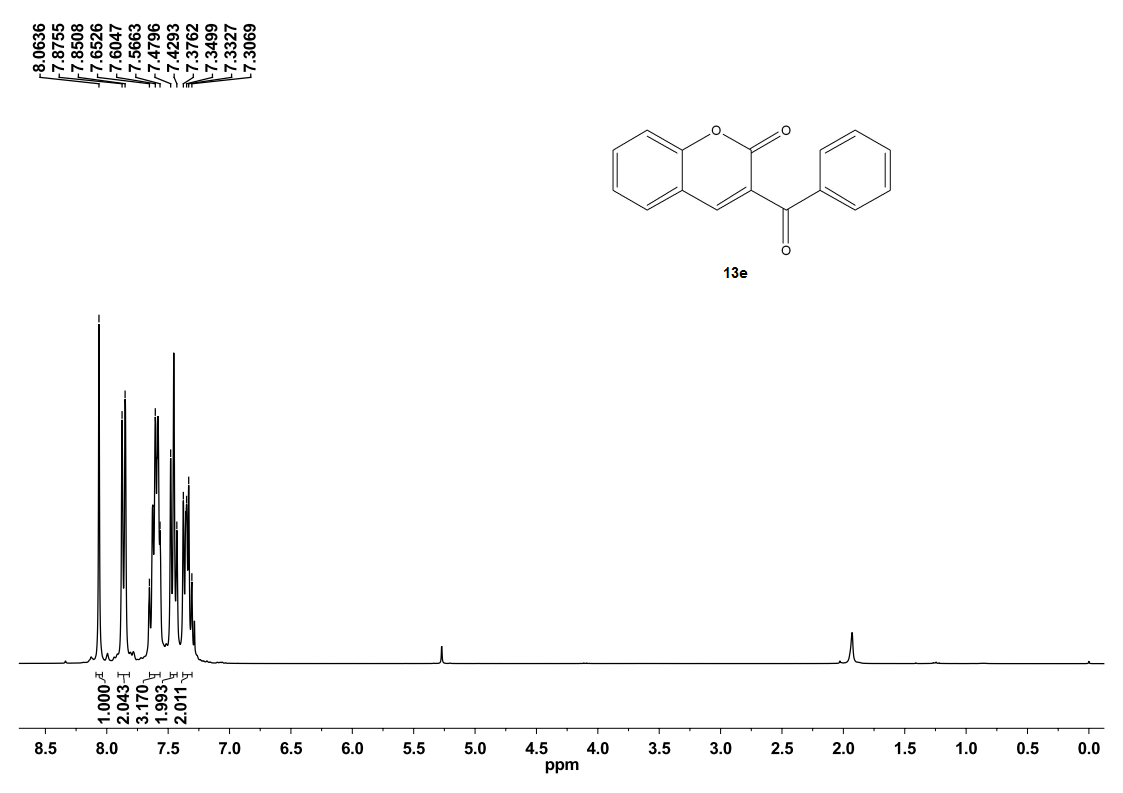


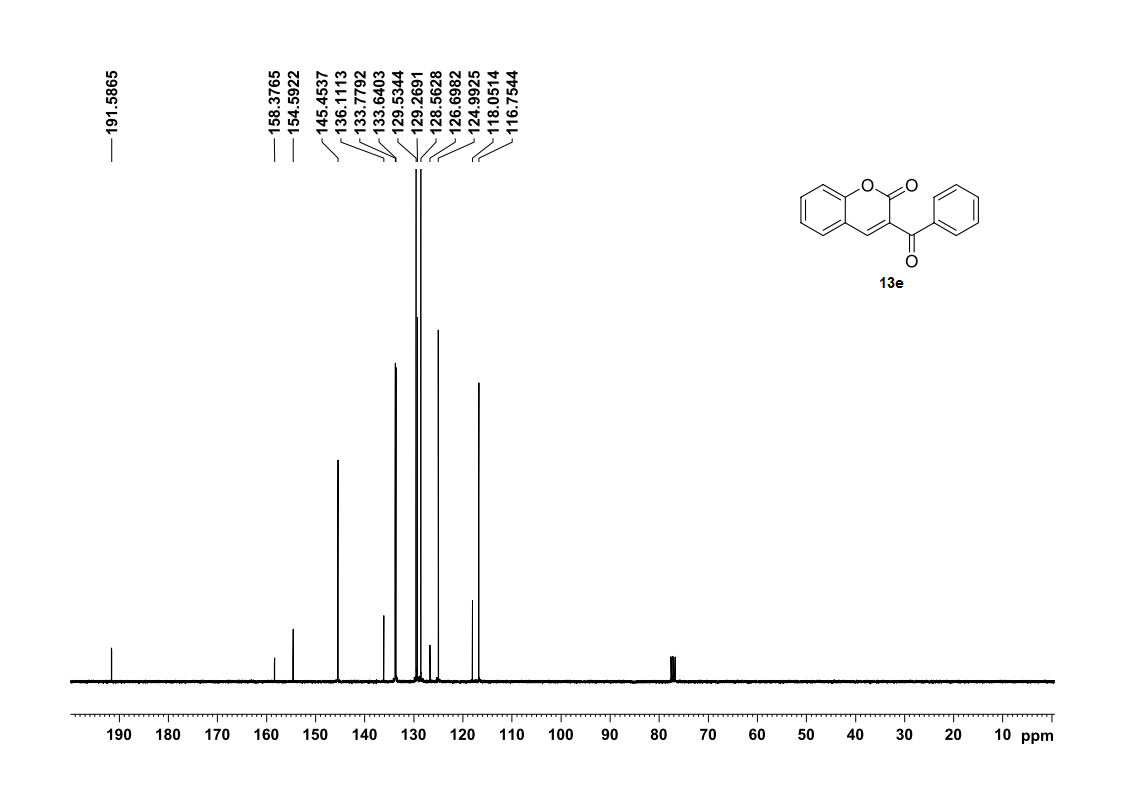


**Ethyl 2-oxo-2*H*-chromene-3-carboxylate (13f):** [[^20^](#_ENREF_6)]

**3-Acetyl-2*H*-chromen-2-one (13g):** [[^20^](#_ENREF_6)]

**Aza-Diels-Alder products**

**3-*endo*-(3-Fluorophenyl)-2-(4-methoxyphenyl)-2-azabicyclo-[2.2.2]octan-5-one (15a):** ^[22]^

**3-*exo*-(3-Fluorophenyl)-2-(4-methoxyphenyl)-2-azabicyclo-[2.2.2]octan-5-one (16a):** ^[22]^

**3-*endo*-(4-Fluorophenyl)-2-(4-methoxyphenyl)-2-azabicyclo-[2.2.2]octan-5-one (15b):** ^[22]^

**3-*exo*-(4-Fluorophenyl) -2-(4-methoxyphenyl)-2-azabicyclo-[2.2.2]octan-5-one (16b):** ^[22]^

**3-*endo*-(4-Fluorophenyl)-2-phenyl-2-azabicyclo-[2.2.2]octan-5-one (15c):** ^[22]^

**3-*exo*-(4-Fluorophenyl)-2-phenyl-2-azabicyclo-[2.2.2]octan-5-one** **(16c):**

**3-*endo*-(3-Chlorophenyl)-2-(4-methoxyphenyl)-2-azabicyclo-[2.2.2]octan-5-one (15d):** ^[23]^

**3-*exo*-(3-Chlorophenyl)-2-(4-methoxyphenyl)-2-azabicyclo-[2.2.2]octan-5-one (16d):** ^[23]^

**3-*endo*-(4-Chlorophenyl)-2-(4-methoxy phenyl)-2-azabicyclo-[2.2.2]octan-5-one (15e):** ^[23]^

**3-*exo*-(4-Chlorophenyl)-2-(4-methoxyphenyl)-2-azabicyclo-[2.2.2]octan-5-one (16e):** ^[23]^
